# Assessment of Molecular Subtypes in Thyrotoxic Periodic Paralysis and Graves Disease Among Chinese Han Adults: A Population-Based Genome-Wide Association Study

**DOI:** 10.1001/jamanetworkopen.2019.3348

**Published:** 2019-05-03

**Authors:** Shuang-Xia Zhao, Wei Liu, Jun Liang, Guan-Qi Gao, Xiao-Mei Zhang, Yu Yao, Hai-Ning Wang, Fei-Fei Yuan, Li-Qiong Xue, Yu-Ru Ma, Le-Le Zhang, Xiao-Ping Ye, Qian-Yue Zhang, Feng Sun, Rui-Jia Zhang, Shao-Ying Yang, Ming Zhan, Wen-Hua Du, Bing-Li Liu, Xia Chen, Zhi-Yi Song, Xue-Song Li, Ping Li, Ying Ru, Chun-Lin Zuo, Sheng-Xian Li, Bing Han, Hui Zhu, Jie Qiao, Miao Xuan, Bin Su, Fei Sun, Jun-Hua Ma, Jia-Lun Chen, Hao-Ming Tian, Sai-Juan Chen, Huai-Dong Song

**Affiliations:** 1 The Core Laboratory in Medical Center of Clinical Research, Department of Endocrinology, Shanghai Ninth People’s Hospital, State Key Laboratory of Medical Genomics, Shanghai Jiaotong University School of Medicine, Shanghai, China; 2 Department of Endocrinology, The Central Hospital of Xuzhou Affiliated to Xuzhou Medical College, Xuzhou, Jiangsu, China; 3 Department of Endocrinology, People’s Hospital of Linyi, Linyi, Shandong, China; 4 Department of Endocrinology, The First Hospital Affiliated to Bengbu Medical College, Bengbu, Anhui, China; 5 Department of Endocrinology and Metabolism, West China Hospital, Sichuan University, Chengdu, Sichuan, China; 6 Department of Endocrinology, Nanjing First Hospital, Nanjing Medical University, Nanjing, Jiangsu, China; 7 Department of Endocrinology, Shanghai Fourth People’s Hospital, Tongji University, Shanghai, China; 8 Department of Endocrinology and Metabolism, Shanghai General Hospital, Shanghai Jiao Tong University, Shanghai, China; 9 Department of Endocrinology and Metabolism, Minhang Hospital, Zhongshan Hospital, Fudan University, Shanghai, China; 10 Department of Endocrinology, Drum Tower Hospital Affiliated to Nanjing University Medical School, Nanjing, Jiangsu, China; 11 Department of Endocrinology, Anhui Provincial Hospital, Hefei, Anhui, China; 12 Department of Endocrinology, the First Affiliated Hospital of Anhui Medical University, Hefei, Anhui, China; 13 Department of Endocrinology, Renji Hospital Affiliated to Shanghai Jiaotong University School of Medicine, Shanghai, China; 14 Department of Endocrinology, Shanghai Tongji Hospital, Tongji University School of Medicine, Shanghai, China; 15 Department of Endocrinology and Metabolism, Shanghai Tenth People's Hospital, Tongji University School of Medicine, Shanghai, China; 16 Department of Endocrinology, Shanghai Pudong New Area Gongli Hospital, Shanghai, China

## Abstract

**Importance:**

Thyrotoxic periodic paralysis (TPP) is a potentially lethal complication of hyperthyroidism. However, only 1 specific susceptibility locus for TPP has been identified. Additional genetic determinants should be detected so that a prediction model can be constructed.

**Objective:**

To investigate the genetic architecture of TPP and distinguish TPP from Graves disease cohorts.

**Design, Setting, and Participants:**

This population-based case-control study used a 2-stage genome-wide association study to investigate the risk loci of TPP and weighted genetic risk score to construct a TPP prediction model with data from a Chinese Han population recruited in hospitals in China from March 2003 to December 2015. The analysis was conducted from November 2014 to August 2016.

**Main Outcomes and Measures:**

Loci specifically associated with TPP risk and those shared with Graves disease and prediction model of joint effects of TPP-specific loci.

**Results:**

A total of 537 patients with TPP (mean [SD] age, 35 [11] years; 458 male) 1519 patients with Graves disease and no history of TPP (mean [SD] age, 38 [13] years; 366 male), and 3249 healthy participants (mean [SD] age, 46 [10] years; 1648 male) were recruited from the Han population by hospitals throughout China. Two new TPP-specific susceptibility loci were identified: *DCHS2* on 4q31.3 (rs1352714: odds ratio [OR], 1.58; 95% CI, 1.35-1.85; *P* = 1.24 × 10^−8^) and *C11orf67* on 11q14.1 (rs2186564: OR, 1.50; 95% CI, 1.29-1.74; *P* = 2.80 × 10^−7^). One previously reported specific locus was confirmed on 17q24.3 near *KCNJ2* (rs312729: OR, 2.08; 95% CI, 1.83-2.38; *P* = 8.02 × 10^−29^). Meanwhile, 2 risk loci (MHC and Xq21.1) were shared by Graves disease and TPP. After 2 years of treatment, the ratio of persistent thyrotropin receptor antibody positivity was higher in patients with TPP than in patients with Graves disease and no history of TPP (OR, 3.82; 95% CI, 2.04-7.16; *P* = 7.05 × 10^−6^). The prediction model using a weighted genetic risk score and 11 candidate TPP-specific single-nucleotide polymorphisms had an area under the curve of 0.80.

**Conclusions and Relevance:**

These findings provide evidence that TPP is a novel molecular subtype of Graves disease. The newly identified loci, along with other previously reported loci, demonstrate the growing complexity of the heritable contribution to TPP pathogenesis. A complete genetic architecture will be helpful to understand the pathophysiology of TPP, and a useful prediction model could prevent the onset of TPP.

## Key Points

**Question:**

Is thyrotoxic periodic paralysis (TPP) a molecular subtype of Graves disease?

**Findings:**

In this case-control study in a Chinese Han population, 5 TPP susceptibility loci were identified, including 3 specific loci and 2 loci shared by Graves disease and TPP. The ratio of persistent thyrotropin receptor antibody positivity was higher in TPP than in Graves disease, and TPP could be predicted from Graves disease using TPP-specific loci.

**Meaning:**

A complete genetic architecture will be helpful to understand the pathophysiology of TPP, and a useful prediction model could prevent the onset of TPP, suggesting TPP as a molecular subtype of Graves disease.

## Teaser

This population-based case-control study uses a 2-stage genome-wide association study to evaluate the association of specific loci with thyrotoxic periodic paralysis and determine whether these loci are also associated with Graves disease in a Chinese Han population.

## Introduction

Thyrotoxic periodic paralysis (TPP) is a potentially life-threatening complication of hyperthyroidism characterized by symptoms that include muscle weakness or paralysis, acute serum hypokalemia, and thyrotoxicosis.^1^ The condition can occur in any ethnicity^2,3^ but predominantly affects Asian populations.^1,4^ Incidence of TPP in Chinese and Japanese patients with thyrotoxicosis is 1.8%^5^ and 1.9%,^6^ respectively. Despite a higher incidence of thyrotoxicosis in women (the female to male ratio is between 4:1 and 10:1), TPP predominantly affects men (the male to female ratio is between 22:1 and 76:1).^3,4^ In Chinese populations, TPP occurs in 13% of male and 0.17% of female patients with thyrotoxicosis.^3^ Based on the higher prevalence of TPP in Asian populations than in European populations,^1,4^ we hypothesize that Asian populations have a genetic predisposition to develop TPP.

The genetic pathogenesis of TPP remains unknown. Recently, a newly identified Kir channel, Kir2.6 (encoded by *KCNJ18*), has been reported to predispose patients with TPP to acute paralytic attacks.^7^ Mutations of *KCNJ18* in 10 of 30 patients (33%) with TPP from Brazil, the United States, and France have been reported.^7^ Kir2.6 loss-of-function mutations triggered a positive feed-forward cycle of hypokalemia leading to muscle inexcitability.^7^ However, Kir2.6 mutations were rarely identified in individuals with TPP from Asian populations.^7,8^ Although 3 genome-wide association studies (GWAS) with small sample sizes showed that chromosome 17q24.3 near *KCNJ2* was the susceptibility locus of TPP, the causal single-nucleotide polymorphism (SNP) in this region remains controversial.^9,10,11^

Based on the missing heritability data and small samples in the previous findings and TPP attacks triggered by any form of hyperthyroidism,^12^ we hypothesize that TPP could be a molecular subtype of hyperthyroidism with a different genetic susceptibility than that of Graves disease (GD). Therefore, the present 2-stage GWAS was carried out in a total of 537 patients with TPP, 1519 patients with GD and no history of TPP, and 3249 healthy participants from the Chinese Han population. In this study, we identified novel TPP risk loci, especially 2 specific TPP loci, and distinguished patients with TPP from those with GD based on TPP-specific susceptibility genes.

## Methods

### Characteristics of Study Participants

In collaboration with hospitals in China, samples were obtained with local institutional review board approval and documented written informed consent from a total of 2056 patients with GD (537 with TPP and 1519 without TPP) and 3249 healthy individuals recruited from Chinese Han populations from March 2003 to December 2015 (Table 1 and eAppendix in the Supplement). The analysis was conducted from November 2014 to August 2016. Reporting of this study followed the Strengthening the Reporting of Observational Studies in Epidemiology (STROBE) reporting guideline and the Strengthening the Reporting of Genetic Association Studies (STREGA) reporting guideline.

**Table 1.** Description of the Sample Sets in the Current Study

Genotyping Stage	Genotyped SNPs, No.	Disease Status	Participants, No.	Male, No./Female, No.	Age at Examination, Mean (SD), y
Discovery	2 752 055	GD with TPP	175	164/11	34 (11)
		Control	2160	601/1559	45 (9)
		GD without TPP	1519	366/1153	38 (13)
Replication	134	TPP	362	294/68	35 (11)
		Control	1089	1047/42	48 (12)
Combined	134	TPP	537	458/79	35 (11)
		Control	3249	1648/1601	46 (10)

Abbreviations: GD, Graves disease; SNP, single-nucleotide polymorphism; TPP, thyrotoxic periodic paralysis.

### GWAS Genotyping and Initial Quality Control

Samples from the TPP GWAS (158 patients with TPP and 803 controls) and GD GWAS (17 patients with TPP, 1519 patients with GD and no history of TPP, and 1516 controls) were genotyped using HumanOmniZhonghua-8 BeadChip and Human660-Quad BeadChip kits^13,14^ (Illumina, Inc), respectively. After robust quality control and imputation analyses (eAppendix and eFigure 1 in the Supplement), association analyses for 2 752 055 SNPs among 175 patients with TPP, 1404 patients with GD and no history of TPP, and 2160 healthy controls were conducted using SNPTEST version 2 software (Oxford University Innovation) (eFigure 2 in the Supplement).^15^

### Evaluation of Population Structure and Quantile-Quantile Plots

To evaluate the population structure in samples, principal component analysis (PCA) (eFigure 3A in the Supplement) and multidimensional scaling (eFigure 3B in the Supplement) analysis were performed by SmartPCA^16^ and PLINK,^17^ respectively. And the distribution of observed *P* values (on the –log_10_ scale) of given SNPs were plotted against the theoretical distribution of expected *P* values to construct quantile-quantile plots (eFigure 4 in the Supplement).

### Genotyping in the Replication Stage

For the replication study, 100 SNPs specifically associated with TPP in 71 chromosomal regions were selected and genotyped in the second cohort (362 patients with TPP, 1089 sex-matched controls) (eTable 1, eTable 2, and eFigure 5 in the Supplement) using TaqMan SNP Genotyping Assays (Applied Biosystems) on the EP1 platform (Fluidigm). To increase statistical power, we also genotyped 100 selected SNPs in the GWAS samples and 34 SNPs within 22 GD susceptibility chromosomal regions in the replication stage using the 7900HT Fast Real Time PCR System (Applied Biosystems) (eTable 3 in the Supplement).

### Association Analyses in Replication and Combined Populations

For autosomal SNPs, we used the Cochran-Armitage trend test in the replication stage and the Cochran-Mantel-Haenszel stratification analysis in combined samples.^17^ The difference among the studies was examined using the Breslow-Day test.^17^ Age- and sex-adjusted odds ratios (ORs) were obtained by logistic regression analysis using PLINK.^17^ Conditional logistic regression analysis was used to examine independent effects of individual SNPs using R statistics packages (R Project for Statistical Computing).

### Prediction Models

We constructed the prediction model using the weighted genetic risk score (wGRS)^18^ and 2 different markers. First, 3 independent TPP-specific SNPs in 3 chromosomal loci were used as the markers to construct the wGRS model. Then, a total of 11 independent SNPs in 8 chromosomal loci with replication stage *P* < .05, with combined analysis *P* < .0005 (Bonferroni-corrected significance in combined populations), and with TPP vs GD *P* < .05 were used as the markers to construct the wGRS model. The value of the weighted score was rescaled by dividing all values by the sum of the effect sizes and then multiplying by the total number of SNPs, thus obtaining the final weighted GRS.^18^ Next, logistic regression was used to calculate the OR and *P* values for each wGRS and sex as a covariant. Subsequently, we generated the receiver operating characteristic (ROC) curves and calculated the area under the curve (AUC) and the sensitivity and specificity of each model to determine how well the models discriminated between the patients with GD and a history of TPP and the patients with GD and no history of TPP. We then categorized the risk scores as 4 different groups according to the mean wGRS and SD *P* values, and ORs and 95% confidence intervals were evaluated using group 1 as the reference.

## Results

### Characteristics of the Samples in This Study

A total of 2056 patients with GD and 3249 healthy participants were recruited from the Chinese Han population through collaboration with the hospitals in China (Table 1). Among 2056 patients with GD, there were 537 patients with TPP and 1519 patients without TPP. Among the 537 patients with TPP, 458 patients were male and 79 were female (Table 1). There were 366 male patients with GD and 1153 female patients with GD who had no history of TPP. The mean (SD) ages of 537 patients with TPP and 3249 healthy participants were 35 (11) and 46 (10) years, respectively (Table 1).

Among the 537 patients with TPP, the age at onset ranged from 13 to 79 years (mean [SD], 32.2 [11.0]) (eTable 4 in the Supplement). All participants with TPP experienced episodes of paraplegia or quadriplegia. The duration of TPP onset ranged from several minutes to 2 days. Fifty-two percent of patients with TPP had inducing factors for paralytic episodes, such as strenuous exercise, emotional stress, and high carbohydrate load. However, 48% of patients did not exhibit precipitating factors of TPP. Before being diagnosed with TPP, 56% of patients had fewer than 3 episodes, 34% had 3 to 5 episodes, and 10% had more than 5 episodes. Serum potassium ranged from 0.5 to 2.25 mEq/L (mean [SD], 2.1 [0.5] mEq/L; reference range, 3.5-5.1 mEq/L) (to convert to millimoles per liter, multiply by 1.0) during the episodes of paraplegia or quadriplegia in patients with TPP, but levels of serum magnesium and phosphorus did not show significant abnormalities.

Unexpectedly, persistent thyrotropin receptor antibody (TRAb) positivity was significantly higher in patients with TPP than in patients with GD and no history of TPP by drug or radioiodine treatment for more than 1 or 2 years (88.8% vs 73.4%, respectively; OR, 2.87; 95% CI, 1.76-4.65; *P* = 8.94 × 10^−6^ after >1 year of treatment and 91% vs 72.5%, respectively; OR, 3.82; 95% CI, 2.04-7.16; *P* = 7.05 × 10^−6^ after >2 years of treatment) (Table 2). Thus, hyperthyroidism relapse was probably higher in patients with TPP than in patients with GD without TPP after discontinuation of antithyroid drug (ATD) treatment.

**Table 2.** The Difference of Consistent TRAb Positivity After Treatment Between Patients With GD With TPP and Patients With GD Without TPP

Course of Disease	GD With TPP			GD Without TPP			*P* Value	OR (95% CI)
	Total No.	TRAb, No. (%)		Total No.	TRAb, No. (%)			
		Negative	Positive		Negative	Positive		
>1 y	169	19 (11.2)	150 (88.8)	2779	740 (26.6)	2039 (73.4)	8.94 × 10^−6^	2.87 (1.76-4.65)
>2 y	122	11 (9.0)	111 (91.0)	2023	556 (27.5)	1467 (72.5)	7.05 × 10^−6^	3.82 (2.04-7.16)

Abbreviations: GD, Graves disease; OR, odds ratio; TPP, thyrotoxic periodic paralysis; TRAb, thyrotropin receptor antibody.

### Identification of TPP-Specific Susceptibility Loci

After the association analyses in the discovery stage (eTable 1 and eFigure 2 in the Supplement), we genotyped 100 TPP-specific SNPs (discovery stage for TPP vs control, *P* < 5 × 10^−4^ and for TPP vs GD, *P* < .05), as well as 34 SNPs in 22 known GD susceptibility regions of 362 TPP cases and 1089 controls (eTable 2, eTable 3, and eFigure 5 in the Supplement). In the combined population, we found 3 loci (rs1352714 at 4q31.3, rs4947296 at 6p21.3, and rs312691 at 17q24.3) were unequivocally associated with TPP, with a genome-wide significance threshold of *P* = 5.0 × 10^−8^, and 2 loci (rs5912838 at Xq21.1 and rs2186564 at 11q14.1) reached the Bonferroni-corrected significance rather than genome-wide significance (Table 3). In 3 of 5 TPP susceptibility loci, SNPs showed significant difference between 533 patients with TPP and 1404 patients with GD and no history of TPP in our combined cohorts, suggesting they were TPP-specific risk loci (Table 3; eTable 2 in the Supplement). Using a liability threshold model, 6.8% of TPP heritability was associated with 5 TPP risk loci and 3.1% with 3 specific TPP susceptibility loci.

**Table 3.** Specific Susceptibility Loci and GD Risk Loci Associated With TPP by 2-Stage GWAS

Chr and SNP	BP	Annotated Genes	Alleles	TPP												Heterogeneity (TPP vs GD)			
				GWAS (171 vs 2160)				Replication (362 vs 1089)				Combined (533 vs 3249)							
				F_TPP	F_Cons	*P* Value	OR (95% CI)	F_TPP	F_Cons	*P* Value	OR (95% CI)	F_TPP	F_Cons	*P* Value	OR (95% CI)	F_TPP	F_GD	*P* Value	OR (95% CI)
4q31.3 rs1352714^a^	155243604	*DCHS2*	T/C	0.22	0.14	3.69 × 10^−5^	1.74 (1.33-2.27)	0.19	0.14	5.03 × 10^−5^	1.51 (1.24-1.84)	0.20	0.14	1.24 × 10^−8^	1.58 (1.35-1.85)	0.20	0.15	7.01 × 10^−6^	1.46 (1.24-1.72)
6p21.3 rs4947296^b^	31058178	*C6orf15*	C/T	0.27	0.15	1.99 × 10^−8^	2.07 (1.61-2.66)	0.25	0.14	1.38 × 10^−13^	2.05 (1.70-2.46)	0.26	0.14	3.08 × 10^−22^	2.06 (1.77-2.39)	0.26	0.21	.001	1.28 (1.11-1.48)
6p21.3 rs1521^b^	31350704	*MICA/HLA-B*	T/C	0.90	0.83	.001	1.80 (1.27-2.57)	0.92	0.79	4.36 × 10^−16^	3.04 (2.30-4.00)	0.91	0.81	3.63 × 10^−18^	2.51 (2.02-3.13)	0.91	0.88	.001	1.45 (1.16-1.81)
6p21.3 rs6457617^b^	32663851	*HLA-DQB1 *and* HLA-DQA2*	T/C	0.64	0.49	1.55 × 10^−7^	1.84 (1.47-2.31)	0.58	0.45	4.35 × 10^−11^	1.69 (1.44-1.97)	0.60	0.47	2.37 × 10^−17^	1.74 (1.53-1.97)	0.60	0.47	5.35 × 10^−5^	0.76 (0.67-0.87)
6p21.3 rs2281388^b^	33060118	*HLA-DPB1 *and* COL11A2*	A/G	0.48	0.33	3.80 × 10^−8^	1.82 (1.46-2.27)	0.46	0.33	4.00 × 10^−12^	1.71 (1.47-1.99)	0.46	0.33	2.46 × 10^−18^	1.74 (1.54-1.98)	0.46	0.44	.11	1.09 (0.96-1.24)
11q14.1 rs2186564^a^	77583266	*C11orf67*	A/G	0.25	0.16	2.40 × 10^−5^	1.75 (1.35-2.27)	0.22	0.17	.0008	1.37 (1.14-1.65)	0.23	0.17	2.80 × 10^−7^	1.50 (1.29-1.74)	0.23	0.18	.0005	1.32 (1.13-1.54)
17q24.3 rs623011^a^	68259446	*KCNJ2* and *CTD-2378E21.1*	A/G	0.63	0.47	1.19 × 10^−8^	1.92 (1.53-2.41)	0.63	0.46	5.33 × 10^−15^	2.00 (1.69-2.38)	0.63	0.46	1.33 × 10^−22^	1.96 (1.72-2.24)	0.63	0.46	4.95 × 10^−20^	1.96 (1.70-2.26)
17q24.3 rs17714860^a^	68272354	*KCNJ2* and *CTD-2378E21.1*	G/A	0.88	0.79	8.65 × 10^−5^	1.91 (1.37-2.66)	0.87	0.80	6.27 × 10^−5^	1.63 (1.28-2.07)	0.87	0.79	2.31 × 10^−8^	1.74 (1.44-2.10)	0.87	0.80	1.25 × 10^−7^	1.71 (1.40-2.08)
17q24.3 rs411079^a^	68291371	*KCNJ2* and *CTD-2378E21.1*	C/A	0.76	0.67	.0005	1.56 (1.21-2.01)	0.77	0.67	2.11 × 10^−7^	1.71 (1.40-2.09)	0.77	0.67	1.26 × 10^−10^	1.64 (1.40-1.91)	0.77	0.67	5.24 × 10^−11^	1.69 (1.44-1.99)
17q24.3 rs312729^a^	68306837	*KCNJ2 *and* CTD-2378E21.1*	A/G	0.64	0.47	2.54 × 10^−10^	2.05 (1.63-2.57)	0.65	0.47	8.75 × 10^−20^	2.11 (1.79-2.48)	0.65	0.47	8.02 × 10^−29^	2.08 (1.83-2.38)	0.65	0.47	3.12 × 10^−27^	2.10 (1.84-2.40)
17q24.3 rs312691^a^	68326338	*CTD-2378E21.1*	C/T	0.64	0.46	1.49 × 10^−10^	2.06 (1.64-2.59)	0.63	0.46	1.31 × 10^−14^	1.99 (1.67-2.37)	0.63	0.46	6.08 × 10^−24^	2.02 (1.77-2.31)	0.63	0.45	7.02 × 10^−24^	2.08 (1.81-2.39)
17q24.3 rs12451295^a^	68376823	*CTD-2378E21.1 *and* SOX9*	C/T	0.63	0.50	3.36 × 10^−6^	1.70 (1.36-2.13)	0.6	0.48	1.32 × 10^−8^	1.63 (1.38-1.94)	0.61	0.49	2.41 × 10^−13^	1.61 (1.41-1.84)	0.61	0.48	3.91 × 10^−12^	1.64 (1.43-1.89)
17q24.3 rs16975792^a^	68433725	*CTD-2378E21.1 *and* SOX9*	G/A	0.67	0.57	.0004	1.51 (1.20-1.91)	0.66	0.57	2.42 × 10^−5^	1.46 (1.22-1.73)	0.66	0.57	4.04 × 10^−8^	1.46 (1.27-1.67)	0.66	0.56	2.81 × 10^−8^	1.50 (1.30-1.73)
Xq21.1 rs5912838^b^	78497118	*ITM2A*	A/C	0.68	0.58	.002	1.50 (1.10-2.07)	0.70	0.58	1.31 × 10^−5^	1.68 (1.36-2.07)	0.69	0.58	5.91 × 10^−8^	1.62 (1.36-1.93)	0.69	0.64	.44	1.25 (1.05-1.49)

Abbreviations: BP, base position; Chr, chromosome; Cons, controls; F, minor allele frequency; GD, Graves disease; GWAS, genome-wide association study; OR, odds ratio; SNP, single-nucleotide polymorphism; TPP, thyrotoxic periodic paralysis.

^a^ For the specific SNPs associated with TPP, the heterogeneity analysis were performed in the total of 533 patients with TPP and 1404 patients with GD.

^b^ For the 5 SNPs associated with GD in the previous study, the heterogeneity analysis was performed in the total of 533 patients with TPP and 5160 patients with GD.

**Figure 1.**
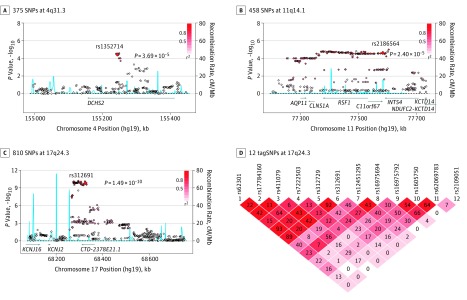
Regional Plots of Thyrotoxic Periodic Paralysis Association at 4q31.3, 11q14.1, and 17q24.3 A-C, The thyrotoxic periodic paralysis association of 375 single-nucleotide polymorphisms (SNPs) at 4q31.3 (A), 458 SNPs at 11q14.1 (B), and 810 SNPs at 17q24.3 (C) in the genome-wide association studies samples. The color of each genotyped SNP spot reflects its *r*^2^, with the top SNP within each association locus shown as a large red diamond and smaller values changing from red to white. Genetic recombination rates, estimated using the 1000 Genomes pilot 1 Han Chinese in Beijing and Japanese in Tokyo data sets, are shown in blue. Physical positions are based on NCBI build 37. D, Linkage disequilibrium block analyses for the 12 tagSNPs at 17q24.3, selecting for genotyping in the replication cohorts.

The SNP rs1352714 at 4q31.3 was a new TPP-specific risk locus that reached genome-wide significance in this study (OR, 1.58; 95% CI, 1.35-1.85; combined *P* = 1.24 × 10^−8^) (Table 3). During the discovery stage, rs1352714, located in exon 13 of *DCHS2* and resulting in an Asn897Ser amino acid substitution, had one of the highest association signals at 4q31.3 (OR, 1.74; 95% CI, 1.33-2.27; discovery stage *P* = 3.69 × 10^−5^) (Figure 1A; eTable 5 in the Supplement). No other SNP at 4q31.3 was independently associated with TPP after accounting for rs1352714 (eFigure 6A in the Supplement). The association was confirmed in replication cohorts (OR, 1.51; 95% CI, 1.24-1.84; replication stage *P* = 5.03 × 10^−5^) (Table 3; eFigure 6B in the Supplement). The frequency of T risk allele rs1352714 in Chinese Han populations (15%) was higher than in European populations (3%) (eTable 2 in the Supplement). Exon resequencing revealed that the frequency of *DCHS2* mutations in patients with TPP (5 of 34) was higher than in control participants (4 of 102) (OR, 4.51; 95% CI, 1.06-16.77; *P* = .03). The frequency of T risk allele rs1352714 was higher in patients with TPP than in those with GD and no history of TPP (OR, 1.46; 95% CI, 1.24-1.72; *P* = 7.01 × 10^−6^) (Table 3), suggesting rs1352714 is specifically associated with TPP.

The association of SNP rs2186564 at 11q14.1 with TPP was confirmed in our replication cohort but did not meet the significance criterion for genome-wide association in combined populations (OR, 1.75; 95% CI, 1.35-2.27; discovery stage *P* = 2.40 × 10^−5^ vs OR, 1.37; 95% CI, 1.14-1.65; replication stage *P* = .0008 vs OR, 1.50; 95% CI, 1.29-1.74; combined *P* = 2.80 × 10^−7^) (Table 3; eTable 6 in the Supplement). The rs2186564 risk allele frequency was much higher in patients with TPP than in those with GD and no history of TPP (OR, 1.32; 95% CI, 1.13-1.54; *P* = .0005) (Table 3), indicating rs2186564 was a novel, specific TPP susceptibility locus. rs2186564 is located in exon 4 of *C11orf67*, causing a Val92Met amino acid substitution (Figure 1B). Interestingly, a cluster of SNPs highly associated with rs2186564 in an approximately 577-kb linkage disequilibrium region at 11q14.1 was correlated with expression of *C11orf67* and *INTS4* in skeletal muscles, CD4^+^ T cells, and naive monocytes (eFigure 7 in the Supplement).

Among 3 specific TPP risk loci, the most significant associations were SNPs rs312691, rs312729, and rs623011 on 17q24.3 (Figure 1C and Table 3). This finding confirmed previous reports that this region is associated with TPP.^9,10,11^ Based on GWAS data, 12 tagSNPs, including rs312691, were selected and genotyped in a replication cohort for refining the association study (Figure 1D; eTable 7 in the Supplement). We found rs312691, rs7222503, or their highly associated SNPs were independent variants at 17q24.3 (OR, 2.02; 95% CI, 1.77-2.31; combined *P* = 6.08 × 10^−24^ for rs312691 and OR, 1.79; 95% CI, 1.45-2.20; combined *P* = 6.17 × 10^−8^ for rs7222503) (Table 3; eTable 8 and eFigure 8 in the Supplement). The risk SNP rs312691 was significant when comparing 533 patients with TPP and 1404 patients with GD and no history of TPP (OR, 2.08; 95% CI, 1.81-2.39; *P* = 7.02 × 10^−24^) (Table 3). Notably, the risk allele C frequency of rs312691 was higher in Chinese Han populations (46%) than in European populations (28%) (eTable 2 in the Supplement). By searching cis-eQTL data of skeletal muscle samples from the GTEx database, we found that a cluster of SNPs highly associated with rs312691 was correlated with *KCNJ2* expression (eFigure 9A in the Supplement). Risk allele C of rs312691 significantly downregulated *KCNJ2* messenger RNA levels in 451 skeletal muscle samples from the GTEx database, as assessed by cis-eQTL analysis (β = −0.23; *P* = 1.19 × 10^−5^) (eAppendix and eFigure 9B in the Supplement).

### TPP Susceptibility Genes Shared With Patients With GD and No History of TPP

In the previous genetic studies of GD, a total of 22 GD risk loci were identified.^13,14,19,20^ Given that the onset of TPP was contingent on the occurrence of thyrotoxicosis, we chose and genotyped 34 SNPs in these 22 well-known GD susceptibility regions in 533 patients in the TPP cohort (eTable 3 in the Supplement). Remarkably, 10 of 34 SNPs in 7 GD susceptibility chromosomal loci showed association with TPP in discovery stage and were confirmed in our replication cohort. The association of 4 SNPs at *HLA* region reached the genome-wide association significance level (*P* < 5.0 × 10^−8^) and 1 SNP at Xq21.1 met the Bonferroni-corrected significance rather than the genome-wide significance level in the combined population (Table 3; eTable 3 in the Supplement). Among the 2 genetic susceptibility chromosome loci shared between patients with TPP and patients with GD without TPP, the most statistically significant association was detected at rs4947296 close to *C6orf15* (OR, 2.06; 95% CI, 1.77-2.39; combined *P* = 3.08 × 10^−22^), rs1521 near *HLA-B* (OR, 2.51; 95% CI, 2.02-3.13; *P* = 3.63 × 10^−18^), rs6457617 near to *HLA-DQB1* (OR, 1.74; 95% CI, 1.53-1.97; *P* = 2.37 × 10^−17^), and rs2281388 near *HLA-DPB1* in the *MHC* region (OR, 1.74; 95% CI, 1.54-1.98; *P* = 2.46 × 10^−18^) (Table 3; eTable 3 in the Supplement). rs5912838 at chromosome X met the Bonferroni-corrected significance rather than the genome-wide significance level in the combined population (OR, 1.62; 95% CI, 1.36-1.93; combined *P* = 5.91 × 10^−8^) (Table 3; eTable 3 in the Supplement).

### Prediction Models Used to Discriminate Between TPP and GD

**Figure 2.**
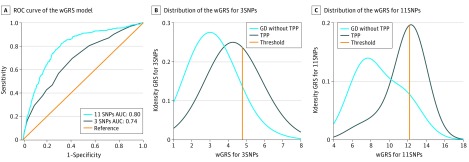
The Weighted Genetic Risk Score (wGRS) Prediction Models of Thyrotoxic Periodic Paralysis (TPP) in Participants With Graves Disease (GD) To predict the potential ratio of TPP seizures in patients with GD, we used the wGRS model to construct the prediction model from 3 and 11 independent single-nucleotide polymorphisms (SNPs) as the markers. The receiver operator characteristic (ROC) curves and the area under the curves (AUCs) of wGRS are shown. A, In 1649 patients with GD and no history of TPP and 491 patients with TPP, the ROC curves of 3 SNPs and 11 SNPs as the markers are shown in navy and blue, respectively, while the reference line is shown in orange. The AUCs of the wGRS model of 3 and 11 SNPs are 0.74 and 0.80, respectively. B and C, The distribution of the wGRS containing 3 SNPs (B) and 11 SNPs (C) in patients with TPP (navy) and with GD having no history of TPP (blue). In wGRS models containing 3 SNPs and 11 SNPs, individuals are classified as having high risk if they had a risk score greater than 4.78 and 12.20, respectively, indicated by the orange solid line. Kdensity indicates kernel density estimation.

The prediction wGRS model of all participants with GD containing 3 independent TPP-specific risk SNPs and 11 candidate TPP-specific SNPs had AUCs of 0.74 and 0.80, respectively (Figure 2A). Sensitivity and specificity of the prediction models were also evaluated, and the wGRS model containing 11 SNPs had higher sensitivity (82%) and less specificity (68%), causing a proportion of individuals with GD without TPP to be misclassified as having TPP (eTable 9 in the Supplement). The OR of each wGRS group increased in parallel with the level of TPP risk. As for the wGRS model containing 3 SNPs, individuals (31% of cases and 8% of controls) in group 3 and 4 with the most risk alleles had approximately 7.55 to 9.46 times greater risk of developing TPP than those in group 1 (Figure 2B; eTable 9 in the Supplement). Meanwhile, in the wGRS model containing 11 SNPs, individuals (46% of cases and 11.5% of controls) in group 3 and 4 with the most risk alleles had approximately 16.27 to 18.76 times greater risk of developing TPP than those in group 1 (Figure 2C; eTable 9 in the Supplement).

## Discussion

Using a 2-stage GWAS in 533 patients with TPP, 1404 patients with GD and no history of TPP, and 3249 healthy individuals, we identified 5 TPP risk loci, including 3 TPP-specific loci and 2 loci shared with GD. Three chromosomal regions (*KCNJ2-CTD-2378E21.1* on 17q24.3, *DCHS2* on 4q31.3, and *HLA*) were unequivocally associated with TPP using a GWAS threshold of *P* = 5 × 10^−8^, and 2 loci (*C11orf67* on 11q14.1 and *ITM2A* on Xq21.1) met the Bonferroni-corrected significance rather than the GWAS criterion. Two of 3 specific TPP susceptibility loci were identified for the first time, to our knowledge, to be associated with TPP in the Chinese Han population. Identification of 3 TPP-specific susceptibility loci in this study provided evidence that TPP is a new GD molecular subtype with specific risk genes. The frequency of risk allele SNPs in 3 TPP-specific susceptibility loci in Chinese Han populations was much higher than in European populations, which at least partially explains TPP predominance in Asian populations. The prediction model using wGRS and 11 candidate TPP-specific SNPs had an AUC of 0.80.

Patients with TPP have episodes of paralysis only while they have thyrotoxicosis. Although any form of hyperthyroidism can cause TPP, most TPP occurs with GD. Patients with TPP are typically first diagnosed with periodic paralysis,^21,22^ and signs and symptoms of thyrotoxicosis in TPP may be subtle. In several previous studies,^22,23,24^ only 10% to 30% of the patients showed symptomatic hyperthyroidism at presentation of episodes of TPP. No palpable goiter is found clinically in most patients.^24,25^ Hyperthyroidism in patients with TPP is often mild at TPP outset and, thus, risk of hyperthyroidism relapse should be lower after discontinuing ATD treatment. We found that the percentage of persistent TRAb positivity was significantly higher in patients with TPP than in those with GD and no history of TPP after drug or radioiodine treatment for more than 1 or 2 years. Because positive TRAb before discontinuing ATD is the best predictor of GD relapse,^26,27^ these findings indicate that TPP should not be treated by ATD. Indeed, 1 recent study^28^ found that all 8 patients with TPP developed thyrotoxic relapse after ATD withdrawal, even if they were treated with ATD for 37.5 (range, 22-247) months. Moreover, patients with TPP who received lower sodium iodide I 131 levels had an unsatisfactory overall remission rate of 28.6%.^28,29^ Therefore, our data and other findings suggest that TPP, with specific symptoms and response to treatments caused by pathways that differ from that underlying GD, should not be treated by ATD, and a median or high dose of sodium iodide I 131 is necessary to rapidly control thyrotoxicosis.

Association of an SNP cluster at 17q24.3 with TPP has been established in previous studies^9,10,11^ and confirmed here. rs312732 (risk allele A), a proxy of rs312691 (*r*^2^ = 0.94) located in exon 3 of long intergenic noncoding RNA *CTD-2378E21.1*, correlated with *KCNJ2* expression in C2C12 skeletal muscle cells.^11^ By searching cis-eQTL data of skeletal muscle samples from the GTEx database, we found a cluster of SNPs highly associated with rs312691 that correlated with *KCNJ2* expression. Risk allele C of rs312691 downregulated *KCNJ2* expression compared with reference allele T. *KCNJ2* mutations could lead to Andersen-Tawil syndrome, a disease characterized by periodic paralysis, cardiac arrhythmias, and dysmorphic features.^30^ Moreover, thyrotoxicosis dramatically promoted periodic paralysis in a patient with Andersen-Tawil syndrome with a de novo c.G899C mutation in *KCNJ2*.^31^

The second highest signal specifically associated with TPP was rs1352714, located in exon 13 of *DCHS2* on 4q31.3, resulting in an Asn897Ser amino acid substitution. DCHS2, an atypical calcium-dependent cell-adhesion protein, regulates planar cell polarity, tissue size, and cell adhesion. DCHS2 plays a role in controlling cartilage differentiation and polarity during craniofacial development^32^ and is associated with neurofibrillary tangle progression, a neuropathological hallmark of Alzheimer disease due to intraneuronal aggregates of highly phosphorylated microtubule-associated protein tau.^33^ Many SNPs in the *DCHS2* region have been identified by GWAS to be associated with the age at onset of Alzheimer disease^34^ or with human facial feature variants such as nose columella inclination.^35^ However, how *DCHS2* variants are involved in TPP pathogenesis remains unknown.

Thyrotoxic periodic paralysis is a potentially lethal complication of hyperthyroidism and resolves when thyroid hormone levels are normalized. Therefore, predicting and identifying TPP in patients with hyperthyroidism is critical to prevent TPP episodes. However, no method to differentially diagnose TPP in hyperthyroidism patients without TPP has been identified before patients have muscle paralysis and hypokalemia episodes. With 3 independent SNPs in 3 specific TPP risk loci and 11 candidate TPP-specific SNPs as predictive markers, we established the prediction model to differentiate patients with TPP from those with GD. Notably, the prediction model containing 11 candidate TPP-specific SNPs with AUCs of 0.80 was usable to differentiate patients with TPP from those with GD. However, the genetic risk score analysis is probably not ready for clinical implementation if treatment decisions are to be based on the predictions.

### Limitations

There are 4 main limitations in this study. First, the sample size of patients with TPP is not large enough to detect more TPP risk loci. Second, we did not perform the follow-up study for the patients with TPP recruited in our study. Third, we do not have a verification model for the prediction model. Fourth, we did not test the patients with GD in resequencing substudy (eTable 10 in the Supplement). Further extensive studies and larger samples will be required before definitive conclusions can be drawn.

## Conclusions

In this study, we identified 4 novel TPP risk loci and confirmed 1 previous reported TPP locus. Of these, 3 loci were TPP-specific and 2 loci were shared with GD. The newly identified loci, along with other previously reported loci, demonstrate the growing complexity of the heritable contribution to TPP pathogenesis. A complete genetic architecture will be helpful to understand the pathophysiology of TPP.

The percentage of persistent TRAb positivity was higher in patients with TPP after 1 or 2 years of treatment than in patients with GD without TPP. Based on these specific TPP susceptibility loci as the predictive markers, we established the prediction model to differentiate patients with TPP from those with GD. These findings may help identify patients with TPP and could provide these patients with better preventive and predictive treatments.

Our findings provide the first evidence, to our knowledge, that TPP is a molecular subtype of GD, with specific symptoms and response to treatments, caused by pathways that differ from those underlying other types of GD.

## Notes

Supplement. **eAppendix.** Supplementary Material **eReferences** **eTable 1.** A Total of 100 SNPs Selected for the Replication Stage Study **eTable 2.** Association Results With TPP in the Initial Genome-Wide Scan, the Replication Stage Analysis and the Combined Populations for the 100 SNPs **eTable 3.** Association Results With TPP in the Initial Genome-Wide Scan and the Second-Stage Analysis for the 34 GD Risk SNPs **eTable 4.** Clinical and Laboratory Features of 537 TPP Patients **eTable 5.** TPP Association Results for the Imputed and Typed SNPs in the 4q31.3 Region in the Initial Genome-Wide Scan **eTable 6.** TPP Association Results for the Imputed and Typed SNPs in the 11q14.1 Region in the Initial Genome-Wide Scan **eTable 7.** TPP Association Results for the Imputed and Typed SNPs in the 17q24.3 Region in the Initial Genome-Wide Scan **eTable 8.** Logistic Regression Analysis for SNPs in the Two Regions Associated With TPP in the Combined Population **eTable 9.** The Association of Each Prediction Model With TPP in Total GD Participants **eTable 10.** Primers of Three TPP Candidate Genes and for Real-Time Reverse Transcription PCR **eFigure 1.** The Flowchart for Quality Filtering in the Two-Stage Study for Genome-Wide Association Study **eFigure 2.** Summary of Association Results From the Genome-Wide Scan **eFigure 3.** Plots of Principal Component Analysis (PCA) and Multidimensional Scaling (MDS) Analysis in Our Cohorts and the HapMap Samples **eFigure 4.** Quantile-Quantile Plot for Detection of Population Stratification **eFigure 5.** The Flowchart for SNPs Selection of the Second Stage Study **eFigure 6.** The Logistic Regression Results and Linkage Disequilibrium Block Analysis at 4q31.3 **eFigure 7.** The Cis-eQTL Analyses Results at 11q14.1 **eFigure 8.** The Logistic Regression Results at 17q24.3 **eFigure 9.** Cis-eQTL Analysis and Transcript Abundances of KCNJ2 at 17q24.3
