# Supplementary material for: Assessment of Molecular Subtypes in Thyrotoxic Periodic Paralysis and Graves Disease Among Chinese Han Adults: A Population-Based Genome-Wide Association Study
Source: JAMA Netw Open. 2019 May 3;2(5):e193348. doi: 10.1001/jamanetworkopen.2019.3348 (PMC6503496; doi:10.1001/jamanetworkopen.2019.3348)
Supplement: Supplement. — eAppendix. Supplementary Material eReferences eTable 1. A Total of 100 SNPs Selected for the Replication Stage Study eTable 2. Association Results With TPP in the Initial Genome-Wide Scan, the Replication Stage Analysis and the Combined Populations for the 100 SNPs eTable 3. Association Results With TPP in the Initial Genome-Wide Scan and the Second-Stage Analysis for the 34 GD Risk SNPs eTable 4. Clinical and Laboratory Features of 537 TPP Patients eTable 5. TPP Association Results for the Imputed and Typed SNPs in the 4q31.3 Region in the Initial Genome-Wide Scan eTable 6. TPP Association Results for the Imputed and Typed SNPs in the 11q14.1 Region in the Initial Genome-Wide Scan eTable 7. TPP Association Results for the Imputed and Typed SNPs in the 17q24.3 Region in the Initial Genome-Wide Scan eTable 8. Logistic Regression Analysis for SNPs in the Two Regions Associated With TPP in the Combined Population eTable 9. The Association of Each Prediction Model With TPP in Total GD Participants eTable 10. Primers of Three TPP Candidate Genes and for Real-Time Reverse Transcription PCR eFigure 1. The Flowchart for Quality Filtering in the Two-Stage Study for Genome-Wide Association Study eFigure 2. Summary of Association Results From the Genome-Wide Scan eFigure 3. Plots of Principal Component Analysis (PCA) and Multidimensional Scaling (MDS) Analysis in Our Cohorts and the HapMap Samples eFigure 4. Quantile-Quantile Plot for Detection of Population Stratification eFigure 5. The Flowchart for SNPs Selection of the Second Stage Study eFigure 6. The Logistic Regression Results and Linkage Disequilibrium Block Analysis at 4q31.3 eFigure 7. The Cis-eQTL Analyses Results at 11q14.1 eFigure 8. The Logistic Regression Results at 17q24.3 eFigure 9. Cis-eQTL Analysis and Transcript Abundances of KCNJ2 at 17q24.3 [file jamanetwopen-2-e193348-s001.pdf]

Supplementary Online Content

Zhao S-X, Liu W, Liang, J, et al; China Consortium for the Genetics of Autoimmune Thyroid Disease. Assessment of molecular subtypes in thyrotoxic periodic paralysis and Graves disease among Chinese Han adults: a population-based genome-wide association study. *JAMA Netw Open*. 2019;2(5):e193348. doi:10.1001/jamanetworkopen.2019.3348

eAppendix. Supplementary Material

eReferences

eTable 1. A Total of 100 SNPs Selected for the Replication Stage Study

eTable 2. Association Results With TPP in the Initial Genome-Wide Scan, the Replication Stage Analysis and the Combined Populations for the 100 SNPs

eTable 3. Association Results With TPP in the Initial Genome-Wide Scan and the Second-Stage Analysis for the 34 GD Risk SNPs

eTable 4. Clinical and Laboratory Features of 537 TPP Patients

eTable 5. TPP Association Results for the Imputed and Typed SNPs in the 4q31.3 Region in the Initial Genome-Wide Scan

eTable 6. TPP Association Results for the Imputed and Typed SNPs in the 11q14.1 Region in the Initial Genome-Wide Scan

eTable 7. TPP Association Results for the Imputed and Typed SNPs in the 17q24.3 Region in the Initial Genome-Wide Scan

eTable 8. Logistic Regression Analysis for SNPs in the Two Regions Associated With TPP in the Combined Population

eTable 9. The Association of Each Prediction Model With TPP in Total GD Participants

eTable 10. Primers of Three TPP Candidate Genes and for Real-Time Reverse Transcription PCR

eFigure 1. The Flowchart for Quality Filtering in the Two-Stage Study for Genome-Wide Association Study

eFigure 2. Summary of Association Results From the Genome-Wide Scan

eFigure 3. Plots of Principal Component Analysis (PCA) and Multidimensional Scaling (MDS) Analysis in Our Cohorts and the HapMap Samples

eFigure 4. Quantile-Quantile Plot for Detection of Population Stratification

eFigure 5. The Flowchart for SNPs Selection of the Second Stage Study

eFigure 6. The Logistic Regression Results and Linkage Disequilibrium Block Analysis at 4q31.3

eFigure 7. The Cis-eQTL Analyses Results at 11q14.1

eFigure 8. The Logistic Regression Results at 17q24.3

eFigure 9. Cis-eQTL Analysis and Transcript Abundances of KCNJ2 at 17q24.3

This supplementary material has been provided by the authors to give readers additional information about their work.

© 2019 Zhao S-X et al. *JAMA Network Open.*

eAppendix. Supplementary Material

The characters of study participants

A total of 537 patients with TPP, 1,519 patients with GD having no history of TPP, and 3,249 healthy participants were recruited from Chinese Han population through collaboration with the hospitals in China (Table 1). All samples were obtained under local institutional review board approval and with documented informed consent. All the cases were sporadic. We collected 5-ml blood samples from each participant for DNA preparation and biochemical measurements.

TPP was diagnosed in patients with episodic flaccid paralysis, serum potassium concentration less than 3.5 mmol/liter (normal concentration, 3.5–5.0 mmol/liter) during at least one of the attacks, and thyrotoxicosis. Diagnosis with GD was based on documented clinical and biochemical evidence of hyperthyroidism, diffuse goiter, and the presence of at least one of the following clinical characters: positive thyroid-stimulating hormone receptor antibody (TRAb) tests, exophthalmos, or pretibial myxedema.[1-5] All individuals classified as having TPP or GD were interviewed and examined by the experienced clinicians.

Control participants were over 35 years old. Given that TPP, GD, and other autoimmune thyroid disease have preponderance in the young female population, this age criteria could reduce the number of controls who might develop GD later. For excluding clinical or subclinical autoimmune thyroid disease, the levels of sensitive TSH and TPOAb in control participants were measured using chemiluminescence immunoassay in our laboratory.[1,2]

© 2019 Zhao S-X et al. *JAMA Network Open.*

GWAS genotyping and initial quality control

DNA samples from 158 TPP cases and 803 controls were genotyped using Illumina HumanOmniZhonghua-8 BeadChips (called TPP GWAS section). And DNA samples from 17 GD cases with TPP, 1,519 GD cases without TPP, and 1,516 controls were genotyped using Illumina Human660-Quad BeadChips as described previously (called GD GWAS section).[1,2] Quality filtering was separately performed on SNPs and samples before analysis to ensure robust association tests for the two above-mentioned sections (eFigure 1 in the Supplement). Cryptic relationships between genotyped individuals were measured utilizing pairwise identity-by-descent estimation by PLINK software.[6]

Of the 900,015 markers assayed in the TPP GWAS section, 4,410 that were from the Y or mitochondrial chromosomes or were copy number variant related and Illumina controls were excluded. Next, 16,984 markers with genotype call rates below 98%, or with a minor allele frequency (MAF) less than 0.01, or with significant deviation from Hardy-Weinberg equilibrium in the controls ($P < 10^{-6}$) or in cases ($P < 10^{-10}$) were discarded, leaving 883,031 SNPs for subsequent analysis. After removing 106 samples with low call rates (< 98%, n =18), gender inconsistencies (n =3), and cryptic relatedness (n =81), 855 samples were available for further association analysis (eFigure 1 in the Supplement). Meanwhile, out of the 655,214 markers assayed in the GD GWAS section, 3,185 that were from the Y or mitochondrial chromosomes or were copy number variant related and Illumina controls were removed. Next, 107,867 markers with a genotype call rate below 98%, or with MAF less than 0.01, or with significant deviation from Hardy-Weinberg equilibrium in the controls ($P < 10^{-6}$), were discarded, leaving 547,347 SNPs for the following analysis. After removing 172 samples with low call rates (< 98%, n =18), gender inconsistencies (n =3), and cryptic relatedness (n =81), 2,880 samples were available for further association analysis (eFigure 1 in the Supplement).

© 2019 Zhao S-X et al. *JAMA Network Open.*

Evaluation of population structure and Quantile-quantile plots

To evaluate the population structure in samples, principal component analysis (PCA) and multidimensional scaling (MDS) analysis using a subset of pruned and unlinked markers were performed by SmartPCA (one of the program modules in the EIGENSOFT software package)[7] and PLINK[6], respectively. For the PCA, our samples and the HapMap samples were plotted using the first two eigenvectors produced by smartPCA (eFigure 2A in the Supplement). For MDS analysis, dimensions were calculated based on the IBD pairwise distance among all individuals in both our cohort and the HapMap participants using PLINK.[6] The first two dimensions of the result were plotted (eFigure 2B in the Supplement). SmartPCA was also used to identify potential genetic outliers. The top ten principal components were computed using HapMap populations followed by projection of current cohort participants onto those principal components, and then run the outlier removal (σ threshold =6 with five iterations). Finally, all participants for the association analysis were restricted to Chinese Han Populations ancestry.

The distribution of observed P values (on the –log10 scale) of given SNPs were plotted against the theoretical distribution of expected P values to construct quantile-quantile plots. To account for relatedness and stratification within our TPP cases and healthy control sample sets, we calculated the genomic control inflation factor (λ) by dividing median $\chi 2$ statistics by 0.4563 based on chip markers. The calculations and the plots were done using PLINK and R statistics packages (eFigure 3 in the Supplement).

Imputation and statistical analysis in the discovery stage

The genotype imputation for the GD GWAS section has been carried out as described previously.[1,2] And genotype imputation for the TPP GWAS section was also performed using IMPUTE2 software[8] in the 158 TPP cases and 803 controls. The 1000G phase 1 integrated variant set (Mar 2012) were also used as a reference. For the imputed SNPs, we first removed the indels and SNPs with a relatively low confidence (estimated probability ≤ 0.9). Then the total of 33,676,913 SNPs with genotype call rates below 98%, or with MAF less than 0.01, or with significant deviation from Hardy-Weinberg equilibrium in the controls ($P < 10^{-6}$) or in cases ($P < 10^{-10}$), were excluded.

© 2019 Zhao S-X et al. *JAMA Network Open.*

Finally, the association analysis for 2,752,055 SNPs among the 171 GD cases with TPP, 1,404 GD cases without TPP, and 2,160 healthy controls were carried out using the SNPTEST v2 software (Frequentist Association Tests with score method).[9]

For the 2,692,307 autosomal SNPs in 171 GD cases with TPP and 2,160 controls, or 1,404 GD cases without TPP, and 2,160 healthy controls, we also carried out the association analysis using the Cochran-Armitage trend test in PLINK,[6] respectively. Whereas, we analyzed 59,748 SNPs located in X chromosome using the logistic regression in PLINK according to the following principles: for alleles A and a, males were coded A to 0 and a to 2, and females were coded AA to 0, Aa to 1, and aa to 2, and additionally sex (male =0, female =1) was also automatically included as a covariate.[6]

Genome-wide P-value and regional plots were generated using R software (LocusZoom) or SNAP, version 2.2 software.[10,11]

SNPs selection for the second stage study

Among 2,752,055 SNPs in the discovery stage, there are 1,731 autosomal SNPs with discovery stage $P < 0.0001$ and the P value between patients with TPP and patients with GD having no history of TPP less than 0.05. Firstly, we defined the chromosomal regions based on HapMap recombination rates (the recombination rate more than 30 cM/Mb) and found 1,731 SNPs with discovery stage $P < 0.0001$ located in 163 chromosomal regions. Next, we performed the forward logistic regression analysis using SNPs with the discovery stage $P < 0.01$ to identify the potential causal SNPs using R package. Then, we performed the sample size analysis and further selected 104 SNPs which can meet the genome-wide association significance level ($P \leq 5 \times 10^{-8}$) within the samples size of TPP cases less than 1,000. Also, we exclude 3 SNPs without significant difference for the MAF between 171 GD cases with TPP and 1,404 GD cases without TPP. Given the association of 17q24.3 locus with TPP has been reported in the previous paper, a total of 12 tagSNPs at 17q24.3 (including rs312691) were also selected and genotyped in the second stage study (eTable 2 in the Supplement). Unfortunately, the probe of rs79449227 at

© 2019 Zhao S-X et al. *JAMA Network Open.*

chromosome 6 was not synthesized successfully. Finally, a total of 100 TPP possible candidate SNPs which located within 71 chromosomal regions were selected to be genotyped in the second cohort (362 Patients with TPP and 1,089 sex-matched controls), utilizing TaqMan® SNP Genotyping Assays on the Fluidigm EP1 platform for the second stage study (eTable 3 and eFigure 9 in the Supplement). Also, we genotyped the 100 selected TPP candidate SNPs in the GWAS samples to increase the statistical power.

Meanwhile, we further chose and genotyped 34 SNPs, which were associated with the susceptibility of GD in Chinese Han population and located in 22 chromosomal regions, in the replication stage using the Applied Biosystems 7900HT Fast Real Time PCR System (eTable 4 in the Supplement).

Association analyses in replication and combined populations

For autosomal SNPs, we used the Cochran-Armitage trend test in the replication stage and the Cochran-Mantel-Haenszel stratification analysis in combined samples.[6] The difference among the studies was examined using the Breslow-Day test.[6] Conditional logistic regression analysis was used to examine independent effects of individual SNPs using R statistics packages.

Power calculations

Power calculations were performed using QUANTO (version 1.2.4). Based on our GWAS data, we found that the minimum of 125 (rs9292371) and the maximum of 964 (rs2109051) samples with TPP would be needed with a disease prevalence of 0.01%, and for the significant difference of these 100 selected SNPs at a genome-wide significance level for each variant with risk allele frequencies ranging from 0.02 to 0.91. However, when the 100 selected TPP candidate SNPs were genotyped in approximately 362 Patients with GD and 1,089 controls, we further calculated the total sample sizes needed in the following study according to the discovery and the second replication cohorts. The data showed that the sample pairs needed for the SNPs with replication $P \leq 0.05$ and

© 2019 Zhao S-X et al. *JAMA Network Open.*

combined $P \leq 0.0005$ (the Bonferroni-corrected significance level) ranged from 196 to 1,531 (eTable 2 in the Supplement). Among the 34 GD risk SNPs, we also calculated the sample sizes needed according to the combined populations (eTable 3 in the Supplement).

Cis-eQTL analysis

As for the cis-eQTL analysis, we inspected the following eQTL databases. One was the cell type-specific eQTLs relevant to immunity and inflammation in paired samples of primary monocytes and B cells, purified by positive selection directly from 283 healthy individuals.[12] The second database was the Genotype-Tissue Expression (GTEx) project (https://gtexportal.org/home/) providing valuable insights into the mechanisms of gene regulation by studying human gene expression and regulation in multiple tissues from health individuals. The third database was developed by Fairfax et al. and contained primary CD14+ human monocytes from 432 European volunteers to the inflamatory proxies interferon-γ (IFN-γ) or differing durations (2 or 24 hours) of lipopolysaccharide (LPS) to assess the effect of innate immune stimuli on eQTLs.[13] The fourth database was from Lee et al. and derived dendritic cells from peripheral blood monocytes of healthy individuals (295 Caucasians, 122 African Americans, 117 East Asians) and stimulated them with LPS, influenza virus, or the cytokine IFN-γ to assess natural genetic variants that underlie variation in the host innate immune response to infection.[14] The fifth cis-eQTL database was from Rai T et al. and derived purified CD4+ T cells and monocytes, representing adaptive and innate immunity, in a multi-ethnic cohort of 461 healthy individuals including 112 African Americans, 213 European Americans, and 82 East Asian Americans.[15]

Real-Time PCR

Blood samples (10 ml) were collected from 258 unrelated healthy Chinese Han volunteers and for gene expression analysis in peripheral blood mononuclear cells (PBMCs). Samples with more blood volume (100 ml) were

© 2019 Zhao S-X et al. *JAMA Network Open.*

donated by 25 individuals from the same group for gene expression assay in distinct subpopulations of PBMCs. The CD4+, CD8+, CD14+ and CD19+ subsets of PBMCs were isolated using MACS Column kits (Miltenyi Biotec) according to the manufacturer's instructions. The purity of each cell subpopulation was determined by an LSR II Flow Cytometer (BD Biosciences), and the cell subpopulations with a purity of over 90% were used for real time RT-PCR.16 Real time quantitative reverse transcription PCRs (RT-PCRs) for a series of genes or transcript units were performed in duplicate using the SYBR Green and ABI 7900HT Fast Real-Time PCR System. Expression levels in all samples were normalized to the relative expression level of GAPDH. Primers used for the amplifications were shown in Table S8. We performed statistical analysis of expression data using ANOVA and an unpaired Student's t-test (the two-tail P-value was indicated on the figures). The genotypes of the SNPs were determined using the ABI 7900HT Fast Real-Time PCR System.

Sequencing of two TPP susceptibility genes and gene level association analysis

To further evaluated the association of two TPP susceptibility genes (*DCHS2* and *KCNJ2*) and a novel long intergenic noncoding RNA (lincRNA, CTD-2378E21.1) with TPP, we first amplified and sequenced the exonic regions of three TPP genes in 34 patients with TPP and 102 healthy controls using PCR and Sanger sequencing and the primers were provided in the online eTable 10 in the Supplement. Next, we compared the frequency difference of rare nonsynonymous variants (MAF ≤ 1%), based on 1000 Genomes East Asian (EAS), ESP6500, ExAc EAS, dbSNP 139 and our inhouse database et al, in the three genes using Fisher's method to combine burden test.

The prediction models

We constructed the prediction model using the weighted genetic risk score (wGRS) [16] and two different markers. Firstly, three independent TPP specific SNPs (rs1352714, rs2186564, and rs312691) in three chromosomal loci

© 2019 Zhao S-X et al. *JAMA Network Open.*

were used as the markers to construct the wGRS model. Then, a total of 11 independent SNPs in eight chromosomal loci with $P_{replication} < 0.05$, with $P_{combined} < 0.0005$ (Bonferroni-corrected significance in combined populations), and with $P_{TPPvsGD} < 0.05$, were used as the markers to construct the wGRS model. As for the wGRS model, a weighted score was calculated by multiplying each participant's allele score (0, 1, 2) by the SNP's relative effect size (the natural log of OR in our study) obtained from our association study. Then, the value of the weighted score was rescaled by dividing all values by the sum of the effect sizes and then multiplying by the total number of SNPs, thus obtaining the final weighted GRS.[16] Next, logistic regression was used to calculate the OR and P values for each wGRS and gender as a covariant. Subsequently, we generated the receiver operator characteristic (ROC) curves and calculated the area under the curve (AUC), the sensitivity and specificity of each model to determine how well the models discriminates between TPP cases and Patients with GD without TPP. Finally, we categorized the risk scores as four different groups according to the mean wGRS and S.D.: group one, wGRS less than the mean; group two, wGRS between the mean and the mean + 1 S.D.; group three, wGRS from the mean + 1 S.D. to the mean + 2 S.D.; group four, wGRS greater than the mean + 2 S.D. P values, OR and 95% confidence intervals were evaluated using group 1 as the reference (eTable 9 in the Supplement).

Web resources

Software and Web resources utilized in the study are provided below:

PLINK v1.07,http://pngu.mgh.harvard.edu/~purcell/plink/

R statistical environment version 2.10.0, http://www.r-project.org/

LocusZoom version 1.1, http://locuszoom.sph.umich.edu/locuszoom/

SNAP version 2.2, http://www.broadinstitute.org/mpg/snap/ldsearch.php/

IMPUTE2, http://mathgen.stats.ox.ac.uk/impute/impute_v2.html/

© 2019 Zhao S-X et al. *JAMA Network Open.*

SNPTEST v2 software, https://mathgen.stats.ox.ac.uk/genetics_software/snptest/snptest.html/

GWAS database, http://jjwanglab.org/gwasdb

QUANTO version 1.2.4, http://biostats.usc.edu/Quanto.html

GTEx Portal V6P, https://gtexportal.org/home/

eReferences

1. Chu X, Pan CM, Zhao SX, et al. A genome-wide association study identifies two new risk loci for Graves' disease. *Nat Genet* 2011;43(9):897-901.

2. Zhao SX, Xue LQ, Liu W, et al. Robust evidence for five new Graves' disease risk loci from a staged genome-wide association analysis. *Hum Mol Genet* 2013;22(16):3347-3362.

3. Zhao SX, Pan CM, Cao HM, et al. Association of the CTLA4 gene with Graves' disease in the Chinese Han population. *PLoS One* 2010;5(3):e9821.

4. Zhao SX, Liu W, Zhan M, et al. A refined study of FCRL genes from a genome-wide association study for Graves' disease. *PLoS One* 2013;8(3):e57758.

5. Song HD, Liang J, Shi JY, et al. Functional SNPs in the SCGB3A2 promoter are associated with susceptibility to Graves' disease. *Hum Mol Genet* 2009;18(6):1156-1170.

6. Purcell S, Neale B, Todd-Brown K, et al. PLINK: a tool set for whole-genome association and population-based linkage analyses. *Am J Hum Genet* 2007;81(3):559-575.

7. Price AL, Patterson NJ, Plenge RM, Weinblatt ME, Shadick NA, Reich D. Principal components analysis corrects for stratification in genome-wide association studies. *Nat Genet* 2006;38(8):904-909.

8. Howie BN, Donnelly P, Marchini J. A flexible and accurate genotype imputation method for the next generation of genome-wide association studies. *PLoS Genet* 2009;5(6):e1000529.

9. Marchini J, Howie B, Myers S, McVean G, Donnelly P. A new multipoint method for genome-wide association studies by imputation of genotypes. *Nat Genet* 2007;39(7):906-913.

10. Pruim RJ, Welch RP, Sanna S, et al. LocusZoom: regional visualization of genome-wide association scan results. *Bioinformatics* 2010;26(18):2336-2337.

11. Johnson AD, Handsaker RE, Pulit SL, Nizzari MM, O'Donnell CJ, de Bakker PI. SNAP: a web-based tool for identification

© 2019 Zhao S-X et al. *JAMA Network Open.*

and annotation of proxy SNPs using HapMap. *Bioinformatics* 2008;24(24):2938-2939.

12. Westra HJ, Peters MJ, Esko T, et al. Systematic identification of trans eQTLs as putative drivers of known disease associations. *Nat Genet* 2013;45(10):1238-1243.

13. Fairfax BP, Humburg P, Makino S, et al. Innate immune activity conditions the effect of regulatory variants upon monocyte gene expression. *Science* 2014;343(6175):1246949.

14. Lee MN, Ye C, Villani AC, et al. Common genetic variants modulate pathogen-sensing responses in human dendritic cells. *Science* 2014;343(6175):1246980.

15. Raj T, Rothamel K, Mostafavi S, et al. Polarization of the effects of autoimmune and neurodegenerative risk alleles in leukocytes. *Science* 2014;344(6183):519-523.

16. Qi Q, Chu AY, Kang JH, et al. Sugar-sweetened beverages and genetic risk of obesity. *N Engl J Med* 2012;367(15):1387-1396.

© 2019 Zhao S-X et al. *JAMA Network Open.*

eTable 1. A total of 100 SNPs selected for the replication stage study								
Region No.	Chr.	Independent SNP	BP	TPPvsControl (175 vs 2,160)			TPPvsGD (175 vs 1,404)	
				P-value	OR	Sample Size	*P*-value	OR
1	1	rs6667605	2502780	2.18×10^{-5}	1.63	352	0.0035	1.41
3	1	rs16831558	44502163	2.41×10^{-5}	1.62	282	0.0010	1.46
3	1	rs1007795	44517409	6.12×10^{-5}	1.60	426	0.0014	1.47
4	1	rs1146530	96492489	2.76×10^{-5}	1.99	369	0.0011	1.76
4	1	rs1144303	96496298	4.01×10^{-5}	1.79	429	0.0005	1.67
5	1	rs539799	120208472	0.0025	1.57	373	0.0115	1.47
8	1	rs4271175	182446188	3.36×10^{-5}	1.91	236	0.0014	1.68
9	1	rs7529077	205561372	5.86×10^{-5}	2.87	165	0.0009	2.47
10	1	rs12038605	234036134	0.0015	1.70	674	0.0592	1.39
11	2	rs13400534	1411883	0.0001	1.60	457	0.0075	1.41
13	2	rs10176331	129435535	0.0029	1.41	387	0.0818	1.23
14	2	rs6751710	168975305	3.75×10^{-5}	1.64	440	0.0002	1.56
16	3	rs6806226	3660691	0.0005	1.65	616	0.0189	1.42
17	3	rs16835753	124349120	0.0025	1.40	594	0.0138	1.32
19	4	rs10026043	21407094	0.0010	2.33	834	0.0043	2.09
21	4	rs10022828	40855358	2.19×10^{-6}	3.04	163	0.0015	2.24
22	4	rs28692179	84763256	0.0002	1.53	377	0.0002	1.54
23	4	rs12511622	124615184	0.0036	1.39	571	0.0098	1.35
25	4	rs1352714	155243604	3.69×10^{-5}	1.74	320	8.38×10^{-5}	1.74
25	4	rs17031489	155273743	0.0058	1.37	925	0.0071	1.37
25	4	rs7681004	155332330	0.0014	1.88	808	0.0020	1.85
26	4	rs9312559	175650116	1.57×10^{-5}	1.86	429	0.0004	1.68
27	5	rs6860373	30022683	8.13×10^{-5}	2.57	167	0.0077	2.03
27	5	rs6450743	30111525	4.72×10^{-5}	2.89	158	0.0073	2.23
27	5	rs9292371	30116542	2.56×10^{-6}	3.31	125	0.0144	2.16
28	5	rs271245	53187303	0.0003	1.53	413	0.0014	1.45
28	5	rs697098	53324930	0.0001	1.54	401	0.0003	1.49
29	5	rs7721254	101551842	9.24×10^{-5}	1.97	213	0.0010	1.81
30	5	rs6881055	113141831	5.96×10^{-5}	1.72	215	0.0015	1.61
31	5	rs59538022	142916502	0.0004	1.52	301	0.0026	1.45
33	5	rs2070843	149824012	0.0002	1.70	457	0.0013	1.58
34	5	rs10039049	150517899	0.0005	1.48	556	0.0239	1.30
35	6	rs12529334	18510379	0.0005	2.60	487	0.0049	2.17
37	6	rs61748643	38816505	8.00×10^{-5}	2.88	191	0.0074	2.08
38	6	rs9471082	39320702	0.0018	1.92	625	0.0111	1.69
39	6	rs952880	73871994	0.0043	1.43	427	0.0198	1.34
41	6	rs9485478	101687421	0.0038	1.74	770	0.0387	1.53
41	6	rs77500196	101707830	0.0017	1.86	634	0.0405	1.54
42	6	rs487697	117637300	0.0002	1.54	349	0.0001	1.59
43	6	rs1321813	117828291	0.0011	1.60	670	0.0718	1.32
44	6	rs2356183	118770264	0.0001	1.55	482	0.0034	1.40
44	6	rs12194555	118819212	0.0001	1.55	482	0.0021	1.42
44	6	rs541442	118884098	0.0002	1.51	417	0.0019	1.42
44	6	rs6569026	118985767	0.0005	1.48	581	0.0004	1.52
45	6	rs79449227	169311554	0.0001	2.15	223	0.0136	1.75
47	7	rs2740761	55255986	0.0001	2.00	232	0.0125	1.59
49	8	rs76087603	94044796	4.34×10^{-7}	2.88	130	3.58×10^{-5}	2.49
50	8	rs16876379	108532275	4.38×10^{-7}	2.19	193	9.40×10^{-5}	1.89
51	8	rs62513232	133530721	0.0002	2.17	606	0.0018	1.93
51	8	rs10099843	133534460	0.0004	2.04	244	0.0032	1.84
53	9	rs4741926	4232824	2.82×10^{-5}	1.63	337	0.0002	1.56
53	9	rs10756531	14152436	0.0003	1.54	526	0.0003	1.57

© 2019 Zhao S-X et al. *JAMA Network Open.*

55	9	rs60798663	137755953	7.95×10^{-7}	2.53	134	3.25×10^{-6}	2.46
55	9	rs4841940	137757212	0.0002	2.01	175	0.0006	1.90
56	10	rs10906701	14619045	9.47×10^{-5}	1.57	277	0.0024	1.43
57	11	rs2056892	2773471	8.16×10^{-6}	1.69	440	0.0003	1.57
58	11	rs10500802	14637391	6.49×10^{-6}	1.68	225	3.55×10^{-5}	1.62
59	11	rs7927729	39962707	0.0005	1.94	533	0.0085	1.66
60	11	rs2186564	77583266	2.40×10^{-5}	1.75	327	0.0040	1.48
61	11	rs2450135	77927995	0.0001	1.55	351	0.0061	1.38
63	11	rs10892996	123157134	0.0003	1.51	408	0.0018	1.43
65	12	rs1629029	43490897	1.46×10^{-7}	1.92	223	3.58×10^{-5}	1.70
66	12	rs2641530	52384811	0.0009	1.45	371	0.0167	1.33
67	12	rs10459263	66538259	0.0002	1.54	439	0.0005	1.49
68	12	rs11110393	100881008	0.0004	1.58	575	0.0006	1.57
68	12	rs12317930	100905216	0.0002	1.62	302	0.0002	1.65
69	13	rs12430111	24408790	1.67×10^{-5}	1.63	359	0.0014	1.45
70	13	rs1336679	69684265	0.0010	1.45	646	0.0224	1.31
70	13	rs7338279	69733326	0.0015	1.54	648	0.0040	1.49
71	13	rs9601390	81034504	0.0035	1.54	296	0.0385	1.37
72	13	rs8000037	93966179	0.0009	1.46	382	0.0168	1.32
73	13	rs966724	103347428	8.49×10^{-5}	1.66	329	0.0071	1.43
74	13	rs9555548	109703988	0.0003	2.16	705	0.0008	2.08
75	14	rs8013963	42431239	0.0002	1.93	625	0.0020	1.79
77	14	rs917607	87563049	2.09×10^{-6}	3.49	135	3.30×10^{-5}	3.22
77	14	rs72683475	87586015	0.0004	2.54	195	0.0034	2.24
79	14	rs78647847	102205333	0.0029	1.78	678	0.0492	1.47
79	14	rs1746586	102327818	0.0047	1.50	815	0.0120	1.43
80	15	rs4886647	74110101	0.0002	1.51	447	0.0152	1.33
81	15	rs4779128	79919955	0.0004	1.65	583	0.0089	1.45
84	16	rs12597065	71741624	3.40×10^{-5}	1.65	423	0.0017	1.47
85	16	rs7194974	82373078	0.0009	1.56	527	0.0027	1.51
86	17	rs4790838	1800087	6.70×10^{-5}	1.55	314	0.0014	1.44
87	17	rs2013260	65796533	0.0026	1.46	420	0.0346	1.31
88	17	rs623011	68259446	1.19×10^{-8}	1.92	213	4.48×10^{-7}	1.83
88	17	rs17714860	68272354	8.65×10^{-5}	1.91	427	0.0014	1.74
88	17	rs411079	68291371	0.0005	1.56	572	0.0004	1.59
88	17	rs7222503	68299785	0.0007	1.81	573	0.0079	1.61
88	17	rs312729	68306837	2.54×10^{-10}	2.05	178	2.10×10^{-8}	1.94
88	17	rs312691	68326338	1.49×10^{-10}	2.06	174	4.97×10^{-9}	1.99
88	17	rs12451295	68376823	3.36×10^{-6}	1.70	323	4.06×10^{-6}	1.72
88	17	rs16975694	68380657	0.0012	1.53	651	0.0007	1.58
88	17	rs16975792	68433725	0.0004	1.51	561	0.0004	1.53
88	17	rs1605750	68471073	0.0022	1.49	733	0.0046	1.46
88	17	rs62069783	68492256	0.0016	1.45	710	0.0030	1.43
88	17	rs2109051	68588806	0.0107	1.74	964	0.0057	1.85
89	17	rs2713969	70773785	0.0006	1.47	379	0.0094	1.35
90	18	rs4595868	21693892	6.53×10^{-5}	1.65	264	0.0015	1.51
91	18	rs7235371	33674808	7.17×10^{-5}	1.57	404	0.0005	1.50
93	20	rs2423166	6526125	0.0004	1.65	289	0.0101	1.48
94	20	rs4239695	41981566	0.0017	1.45	367	0.0255	1.32
96	20	rs1570530	55155404	0.0002	2.05	211	0.0295	1.57
97	21	rs1993668	22848277	0.0006	1.90	661	0.0010	1.89
99	22	rs133501	48912283	0.0003	1.52	520	0.0004	1.51

CHR, chromosome; SNP, single nucleotide polymorphism; BP, base position; TPP, thyrotoxic hypokalemic periodic paralysis; OR, odds ratio.

© 2019 Zhao S-X et al. *JAMA Network Open.*

eTable 2. Association results with TPP in the initial genome-wide scan, the replication stage analysis and the combined populations for the 100 SNPs.

Region No.	Chr.	SNP	BP	*Annotated Genes*	Alleles	TPPvsControl Discovery (171 vs 2,160)			
						F_TPP	F_Cons	*P*-value	OR (95% CI)
1	1	rs6667605	2502780	*TNFRSF14/C1orf93*	C/T	0.63	0.51	2.18×10^{-5}	1.63 (1.30-2.04)
3	1	rs16831558	44502163	*SLC6A9/KLF17*	T/C	0.45	0.33	2.41×10^{-5}	1.62 (1.30-2.02)
3	1	rs1007795	44517409	*SLC6A9/KLF17*	G/A	0.66	0.55	6.12×10^{-5}	1.60 (1.27-2.02)
4	1	rs1146530	96492489	*PTBP2/RWDD3*	C/A	0.88	0.78	2.76×10^{-5}	1.99 (1.43-2.78)
4	1	rs1144303	96496298	*PTBP2/RWDD3*	T/C	0.82	0.71	4.01×10^{-5}	1.79 (1.35-2.38)
5	1	rs539799	120208472	*ZNF697/PHGDH*	C/T	0.16	0.11	0.0025	1.57 (1.16-2.13)
8	1	rs4271175	182446188	*RGSL1*	T/G	0.15	0.08	3.36×10^{-5}	1.91 (1.40-2.62)
9	1	rs7529077	205561372	*MFSD4*	T/C	0.05	0.02	5.86×10^{-5}	2.87 (1.68-4.92)
11	2	rs13400534	1411883	*TPO/SNTG2*	T/C	0.72	0.62	0.0001	1.60 (1.26-2.04)
14	2	rs6751710	168975305	*STK39*	G/A	0.68	0.57	3.75×10^{-5}	1.64 (1.30-2.07)
16	3	rs6806226	3660691	*LRRN1/CRBN*	A/G	0.19	0.12	0.0005	1.65 (1.24-2.20)
17	3	rs16835753	124349120	*KALRN*	G/A	0.48	0.40	0.0025	1.40 (1.12-1.74)
19	4	rs10026043	21407094	*KCNIP4*	T/C	0.95	0.90	0.0010	2.33 (1.40-3.89)
21	4	rs10022828	40855358	*APBB2*	A/G	0.06	0.02	2.19×10^{-6}	3.04 (1.86-4.95)
22	4	rs28692179	84763256	AGPAT9/NKX6-1	T/C	0.41	0.32	0.0002	1.53 (1.22-1.91)
23	4	rs12511622	124615184	*SPRY1/ANKRD50*	C/T	0.41	0.34	0.0036	1.39 (1.12-1.74)
25	4	rs1352714	155243604	*DCHS2*	T/C	0.22	0.14	3.69×10^{-5}	1.74 (1.33-2.27)
25	4	rs17031489	155273743	*DCHS2*	G/A	0.63	0.56	0.0058	1.37 (1.09-1.72)
25	4	rs7681004	155332330	*DCHS2*	G/T	0.09	0.05	0.0014	1.88 (1.27-2.79)
26	4	rs9312559	175650116	*GLRA3*	A/G	0.82	0.71	1.57×10^{-5}	1.86 (1.40-2.46)
27	5	rs6860373	30022683	*CDH6/CDH9*	T/C	0.06	0.02	8.13×10^{-5}	2.57 (1.59-4.16)
27	5	rs6450743	30111525	*CDH6/CDH9*	C/T	0.05	0.02	4.72×10^{-5}	2.89 (1.71-4.88)
27	5	rs9292371	30116542	*CDH6/CDH9*	A/G	0.05	0.02	2.56×10^{-6}	3.31 (1.98-5.55)
28	5	rs271245	53187303	*ARL15*	C/A	0.65	0.55	0.0003	1.53 (1.22-1.93)
28	5	rs697098	53324930	*ARL15*	C/T	0.52	0.42	0.0001	1.54 (1.23-1.91)
29	5	rs7721254	101551842	*SLCO4C1/ST8SIA4*	T/C	0.13	0.07	9.24×10^{-5}	1.97 (1.40-2.77)
30	5	rs6881055	113141831	*YTHDC2/KCNN2*	G/A	0.23	0.15	5.96×10^{-5}	1.72 (1.32-2.23)
31	5	rs59538022	142916502	*NR3C1/HMHB1*	G/A	0.37	0.28	0.0004	1.52 (1.21-1.91)
33	5	rs2070843	149824012	*RPS14*	C/A	0.81	0.72	0.0002	1.70 (1.29-2.25)
34	5	rs10039049	150517899	*ANXA6*	T/C	0.54	0.44	0.0005	1.48 (1.19-1.84)
35	6	rs12529334	18510379	*RNF144B/ID4*	A/G	0.96	0.90	0.0005	2.60 (1.51-4.48)
37	6	rs61748643	38816505	*DNAH8*	T/C	0.05	0.02	8.00×10^{-5}	2.88 (1.68-4.93)
38	6	rs9471082	39320702	*KIF6*	C/T	0.93	0.87	0.0018	1.92 (1.26-2.91)

© 2019 Zhao S-X et al. *JAMA Network Open.*

39	6	rs952880	73871994	*KCNQ5*	C/A	0.29	0.23	0.0043	1.43 (1.12-1.82)
41	6	rs9485478	101687421	*GRIK2/ASCC3*	C/A	0.91	0.85	0.0038	1.74 (1.19-2.54)
41	6	rs77500196	101707830	*GRIK2/ASCC3*	T/G	0.92	0.86	0.0017	1.86 (1.25-2.76)
42	6	rs487697	117637300	*ROS1*	C/T	0.35	0.26	0.0002	1.54 (1.22-1.94)
44	6	rs2356183	118770264	*C6orf204/SLC35F1*	G/A	0.41	0.31	0.0001	1.55 (1.24-1.93)
44	6	rs12194555	118819212	*C6orf204*	A/C	0.41	0.31	0.0001	1.55 (1.24-1.93)
44	6	rs541442	118884098	*C6orf204*	T/G	0.50	0.40	0.0002	1.51 (1.21-1.88)
44	6	rs6569026	118985767	*C6orf204*	C/T	0.46	0.36	0.0005	1.48 (1.19-1.85)
47	7	rs2740761	55255986	*EGFR*	T/C	0.12	0.06	0.0001	2.00 (1.40-2.84)
49	8	rs76087603	94044796	*C8orf83/LOC389676*	T/G	0.08	0.03	4.34×10^{-7}	2.88 (1.88-4.40)
50	8	rs16876379	108532275	*ANGPT1/RSPO2*	A/C	0.15	0.08	4.38×10^{-7}	2.19 (1.60-3.00)
51	8	rs62513232	133530721	*KCNQ3/LRRC6*	T/C	0.08	0.04	0.0002	2.17 (1.44-3.26)
51	8	rs10099843	133534460	*KCNQ3/LRRC6*	A/G	0.09	0.04	0.0004	2.04 (1.37-3.05)
53	9	rs4741926	4232824	*GLIS3*	A/C	0.37	0.26	2.82×10^{-5}	1.63 (1.30-2.06)
53	9	rs10756531	14152436	*NFIB*	C/A	0.69	0.59	0.0003	1.54 (1.22-1.95)
55	9	rs60798663	137755953	*FCN2/COL5A1*	A/G	0.10	0.04	7.95×10^{-7}	2.53 (1.73-3.70)
55	9	rs4841940	137757212	*FCN2/COL5A1*	T/C	0.10	0.05	0.0002	2.01 (1.39-2.91)
56	10	rs10906701	14619045	*FAM107B*	C/T	0.36	0.27	9.47×10^{-5}	1.57 (1.25-1.98)
57	11	rs2056892	2773471	*KCNQ1*	C/A	0.67	0.55	8.16×10^{-6}	1.69 (1.34-2.14)
58	11	rs10500802	14637391	*PSMA1*	T/G	0.39	0.27	6.49×10^{-6}	1.68 (1.34-2.10)
59	11	rs7927729	39962707	*LRRC4C/C11orf74*	G/A	0.91	0.84	0.0005	1.94 (1.32-2.85)
60	11	rs2186564	77583266	*C11orf67*	A/G	0.25	0.16	2.40×10^{-5}	1.75 (1.35-2.27)
61	11	rs2450135	77927995	*GAB2*	A/G	0.44	0.34	0.0001	1.55 (1.24-1.93)
63	11	rs10892996	123157134	*CLMP/GRAMD1B*	C/T	0.54	0.44	0.0003	1.51 (1.21-1.88)
65	12	rs1629029	43490897	*ADAMTS20/PRICKLE1*	C/T	0.30	0.18	1.46×10^{-7}	1.92 (1.51-2.45)
66	12	rs2641530	52384811	*ACVR1B*	C/T	0.47	0.38	0.0009	1.45 (1.16-1.80)
67	12	rs10459263	66538259	*TMBIM4*	G/A	0.62	0.51	0.0002	1.54 (1.23-1.93)
68	12	rs11110393	100881008	*NR1H4*	C/T	0.27	0.19	0.0004	1.58 (1.23-2.03)
68	12	rs12317930	100905216	*NR1H4*	G/T	0.25	0.17	0.0002	1.62 (1.25-2.09)
69	13	rs12430111	24408790	*MIPEP*	G/A	0.43	0.31	1.67×10^{-5}	1.63 (1.30-2.04)
70	13	rs1336679	69684265	*KLHL1/PCDH9*	T/C	0.60	0.51	0.0010	1.45 (1.16-1.82)
70	13	rs7338279	69733326	*KLHL1/PCDH9*	T/C	0.79	0.70	0.0015	1.54 (1.18-2.01)
71	13	rs9601390	81034504	*SPRY2/SLITRK1*	A/G	0.17	0.12	0.0035	1.54 (1.15-2.07)
72	13	rs8000037	93966179	*GPC6*	A/C	0.57	0.47	0.0009	1.46 (1.17-1.82)
73	13	rs966724	103347428	*METTL21C/CCDC168*	T/G	0.26	0.17	8.49×10^{-5}	1.66 (1.29-2.14)
74	13	rs9555548	109703988	*MYO16*	C/T	0.93	0.86	0.0003	2.16 (1.41-3.30)

© 2019 Zhao S-X et al. *JAMA Network Open.*

75	14	rs8013963	42431239	*LRFN5/FSCB*	A/G	0.11	0.06	0.0002	1.93 (1.35-2.77)
77	14	rs917607	87563049	*GALC/FLRT2*	T/G	0.05	0.01	2.09×10^{-6}	3.49 (2.02-6.04)
77	14	rs72683475	87586015	*GALC/FLRT2*	A/G	0.05	0.02	0.0004	2.54 (1.49-4.32)
79	14	rs78647847	102205333	*PPP2R5C/DIO3*	A/G	0.91	0.85	0.0029	1.78 (1.21-2.61)
79	14	rs1746586	102327818	*PPP2R5C*	T/C	0.81	0.74	0.0047	1.50 (1.13-1.98)
80	15	rs4886647	74110101	*TBC1D21/C15orf59*	T/C	0.58	0.48	0.0002	1.51 (1.21-1.89)
81	15	rs4779128	79919955	*KIAA1024/MTHFS*	A/C	0.20	0.13	0.0004	1.65 (1.25-2.17)
84	16	rs12597065	71741624	*PHLPP2*	C/T	0.32	0.22	3.40×10^{-5}	1.65 (1.30-2.10)
85	16	rs7194974	82373078	*MPHOSPH6/CDH13*	C/T	0.79	0.71	0.0009	1.56 (1.19-2.03)
86	17	rs4790838	1800087	*RPA1*	G/A	0.46	0.35	6.70×10^{-5}	1.55 (1.25-1.94)
87	17	rs2013260	65796533	*BPTF/NOL11*	C/T	0.73	0.65	0.0026	1.46 (1.14-1.86)
88	17	rs623011	68259446	*KCNJ2/CTD-2378E21.1*	A/G	0.63	0.47	1.19×10^{-8}	1.92 (1.53-2.41)
88	17	rs17714860	68272354	*KCNJ2/CTD-2378E21.1*	G/A	0.88	0.79	8.65×10^{-5}	1.91 (1.37-2.66)
88	17	rs411079	68291371	*KCNJ2/CTD-2378E21.1*	C/A	0.76	0.67	0.0005	1.56 (1.21-2.01)
88	17	rs7222503	68299785	*KCNJ2/CTD-2378E21.1*	G/T	0.89	0.82	0.0007	1.81 (1.27-2.56)
88	17	rs312729	68306837	*KCNJ2/CTD-2378E21.1*	A/G	0.64	0.47	2.54×10^{-10}	2.05 (1.63-2.57)
88	17	rs312691	68326338	*CTD-2378E21.1*	C/T	0.64	0.46	1.49×10^{-10}	2.06 (1.64-2.59)
88	17	rs12451295	68376823	*CTD-2378E21.1/SOX9*	C/T	0.63	0.50	3.36×10^{-6}	1.70 (1.36-2.13)
88	17	rs16975694	68380657	*CTD-2378E21.1/SOX9*	T/C	0.78	0.70	0.0012	1.53 (1.18-1.99)
88	17	rs16975792	68433725	*CTD-2378E21.1/SOX9*	G/A	0.67	0.57	0.0004	1.51 (1.20-1.91)
88	17	rs1605750	68471073	*CTD-2378E21.1/SOX9*	A/G	0.77	0.69	0.0022	1.49 (1.15-1.93)
88	17	rs62069783	68492256	*CTD-2378E21.1/SOX9*	C/T	0.69	0.60	0.0016	1.45 (1.15-1.84)
88	17	rs2109051	68588806	*CTD-2378E21.1/SOX9*	A/C	0.93	0.89	0.0107	1.74 (1.13-2.69)
89	17	rs2713969	70773785	*SLC39A11*	C/T	0.46	0.37	0.0006	1.47 (1.18-1.83)
90	18	rs4595868	21693892	*TTC39C*	T/C	0.27	0.18	6.53×10^{-5}	1.65 (1.29-2.12)
91	18	rs7235371	33674808	*SLC39A6/RPRD1A*	A/G	0.53	0.42	7.17×10^{-5}	1.57 (1.26-1.96)
93	20	rs2423166	6526125	*BMP2/FERMT1*	T/C	0.19	0.13	0.0004	1.65 (1.25-2.19)
94	20	rs4239695	41981566	*SRSF6/PTPRT*	A/G	0.35	0.27	0.0017	1.45 (1.15-1.83)
96	20	rs1570530	55155404	*C20orf107/TFAP2C*	G/A	0.09	0.05	0.0002	2.05 (1.39-3.02)
97	21	rs1993668	22848277	*NCAM2*	A/G	0.10	0.06	0.0006	1.90 (1.31-2.74)
99	22	rs133501	48912283	*FAM19A5*	C/T	0.64	0.54	0.0003	1.52 (1.21-1.91)

© 2019 Zhao S-X et al. *JAMA Network Open.*

**Continued eTable 2**										
SNP	TPPvsControl Replication (362 vs 1,089)				TPPvsControl Combined (533 vs 3,249)					
	F_TPP	F_Cons	*P*-value	OR (95% CI)	F_TPP	F_Cons	*P*-value	OR (95% CI)	Breslow-Day Test P-value	Sample size
rs6667605	0.54	0.53	0.8128	1.02 (0.86-1.21)	0.57	0.52	0.0053	1.22 (1.07-1.39)	0.0012	2364
rs16831558	0.36	0.33	0.1547	1.14 (0.95-1.36)	0.39	0.33	0.0002	1.27 (1.11-1.46)	0.0145	1731
rs1007795	0.57	0.57	0.9902	1.00 (0.84-1.18)	0.60	0.56	0.0139	1.19 (1.04-1.36)	0.0012	3174
rs1146530	0.81	0.80	0.4638	1.08 (0.87-1.34)	0.83	0.79	0.0017	1.33 (1.12-1.57)	0.0022	1909
rs1144303	0.75	0.72	0.0945	1.17 (0.97-1.42)	0.77	0.72	0.0001	1.34 (1.15-1.56)	0.0144	1461
rs539799	0.10	0.13	0.0576	0.77 (0.58-1.01)	0.12	0.12	0.8166	1.05 (0.86-1.29)	0.0004	89821
rs4271175	0.11	0.10	0.5294	1.09 (0.83-1.44)	0.12	0.09	0.0034	1.41 (1.15-1.72)	0.0078	2017
rs7529077	0.02	0.02	0.5984	0.86 (0.48-1.53)	0.03	0.02	0.0571	1.53 (1.03-2.26)	0.0018	5063
rs13400534	0.67	0.63	0.0490	1.20 (1.00-1.44)	0.69	0.62	8.40×10^{-5}	1.35 (1.17-1.55)	0.0607	1167
rs6751710	0.61	0.61	0.9922	1.00 (0.84-1.19)	0.63	0.58	0.0106	1.24 (1.08-1.41)	0.0008	2126
rs6806226	0.13	0.12	0.3566	1.12 (0.87-1.44)	0.15	0.12	0.0044	1.27 (1.06-1.53)	0.0441	3215
rs16835753	0.40	0.43	0.1509	0.88 (0.74-1.05)	0.42	0.41	0.5007	1.07 (0.94-1.22)	0.0012	20717
rs10026043	0.91	0.90	0.8287	1.03 (0.77-1.38)	0.92	0.90	0.0263	1.31 (1.03-1.66)	0.0055	4034
rs10022828	0.03	0.03	0.5790	1.14 (0.71-1.82)	0.04	0.02	0.0022	1.83 (1.31-2.55)	0.0035	1535
rs28692179	0.32	0.32	0.9256	0.99 (0.83-1.19)	0.35	0.32	0.0275	1.16 (1.01-1.32)	0.0033	4675
rs12511622	0.31	0.38	0.0006	0.73 (0.61-0.87)	0.34	0.35	0.3337	0.96 (0.84-1.10)	8.53×10^{-6}	66505
rs1352714	0.19	0.14	5.03×10^{-5}	1.51 (1.24-1.84)	0.20	0.14	1.24×10^{-8}	1.58 (1.35-1.85)	0.4052	759
rs17031489	0.59	0.56	0.1775	1.13 (0.95-1.34)	0.61	0.56	0.0062	1.21 (1.06-1.38)	0.1782	2649
rs7681004	0.08	0.05	0.0079	1.57 (1.12-2.20)	0.08	0.05	5.56×10^{-5}	1.68 (1.31-2.15)	0.4973	1361
rs9312559	0.69	0.73	0.0298	0.82 (0.68-0.98)	0.73	0.71	0.3235	1.08 (0.93-1.25)	1.36×10^{-6}	19768
rs6860373	0.03	0.02	0.5568	1.18 (0.69-2.01)	0.04	0.02	0.0027	1.60 (1.12-2.27)	0.0309	2739
rs6450743	0.02	0.02	0.3234	1.35 (0.75-2.45)	0.03	0.02	0.0006	1.81 (1.23-2.66)	0.0562	2355
rs9292371	0.03	0.04	0.1432	0.66 (0.40-1.12)	0.03	0.02	0.2643	1.47 (1.02-2.12)	4.68×10^{-6}	4279
rs271245	0.52	0.57	0.0339	0.83 (0.70-0.98)	0.56	0.55	0.5832	1.04 (0.91-1.18)	2.32×10^{-5}	60836
rs697098	0.43	0.46	0.1951	0.89 (0.75-1.06)	0.46	0.43	0.1880	1.13 (1.00-1.29)	0.0001	6232
rs7721254	0.07	0.08	0.5261	0.90 (0.65-1.24)	0.09	0.07	0.0650	1.27 (1.01-1.60)	0.0009	5478
rs6881055	0.16	0.18	0.4664	0.92 (0.73-1.15)	0.19	0.16	0.0653	1.21 (1.02-1.43)	0.0004	4377
rs59538022	0.25	0.30	0.0131	0.78 (0.65-0.95)	0.29	0.29	0.8546	1.02 (0.89-1.18)	1.11×10^{-5}	284645
rs2070843	0.72	0.74	0.2954	0.90 (0.75-1.09)	0.75	0.72	0.1386	1.13 (0.97-1.31)	0.0002	8136
rs10039049	0.46	0.45	0.6139	1.05 (0.88-1.24)	0.49	0.44	0.0110	1.19 (1.05-1.36)	0.0140	3064
rs12529334	0.92	0.92	0.6406	1.08 (0.79-1.48)	0.94	0.91	0.0082	1.49 (1.15-1.94)	0.0050	2181
rs61748643	0.03	0.02	0.0246	1.77 (1.06-2.94)	0.04	0.02	3.15×10^{-5}	2.17 (1.51-3.12)	0.1905	1242
rs9471082	0.86	0.88	0.1612	0.84 (0.65-1.07)	0.88	0.87	0.4400	1.08 (0.88-1.31)	0.0006	35732
rs952880	0.24	0.23	0.6227	1.05 (0.86-1.28)	0.26	0.23	0.0312	1.17 (1.01-1.36)	0.0556	5033
rs9485478	0.87	0.87	0.9668	1.00 (0.77-1.28)	0.88	0.86	0.0743	1.25 (1.02-1.53)	0.0157	4267
rs77500196	0.87	0.87	0.9597	0.99 (0.77-1.28)	0.89	0.86	0.0583	1.25 (1.02-1.53)	0.0082	4554
rs487697	0.30	0.29	0.4812	1.07 (0.89-1.29)	0.32	0.27	0.0052	1.26 (1.09-1.45)	0.0156	2026
rs2356183	0.35	0.33	0.1162	1.14 (0.97-1.34)	0.37	0.32	0.0005	1.27 (1.11-1.45)	0.0308	1774
rs12194555	0.36	0.31	0.0401	1.21 (1.01-1.45)	0.37	0.31	6.47×10^{-5}	1.32 (1.15-1.51)	0.0938	1307
rs541442	0.46	0.42	0.0428	1.17 (1.01-1.37)	0.47	0.41	0.0002	1.28 (1.13-1.45)	0.0677	1531
rs6569026	0.40	0.38	0.2077	1.12 (0.94-1.33)	0.42	0.37	0.0018	1.25 (1.09-1.42)	0.0478	1917
rs2740761	0.06	0.06	0.6323	0.92 (0.64-1.31)	0.08	0.06	0.0384	1.26 (0.98-1.61)	0.0021	6769
rs76087603	0.04	0.03	0.2803	1.29 (0.81-2.03)	0.05	0.03	6.06×10^{-5}	1.79 (1.31-2.43)	0.0099	1674
rs16876379	0.08	0.09	0.6465	0.93 (0.68-1.27)	0.10	0.08	0.0075	1.34 (1.08-1.67)	0.0001	3156
rs62513232	0.05	0.04	0.2235	1.27 (0.86-1.88)	0.06	0.04	0.0011	1.56 (1.19-2.06)	0.0637	2368
rs10099843	0.05	0.05	0.5753	1.11 (0.76-1.62)	0.06	0.05	0.0099	1.44 (1.10-1.89)	0.0294	2993
rs4741926	0.29	0.28	0.8072	1.02 (0.85-1.23)	0.31	0.27	0.0061	1.25 (1.08-1.43)	0.0019	2177

© 2019 Zhao S-X et al. *JAMA Network Open.*

rs10756531	0.58	0.60	0.2732	0.91 (0.77-1.08)	0.62	0.60	0.1771	1.09 (0.95-1.24)	0.0004	13112
rs60798663	0.06	0.05	0.3868	1.17 (0.82-1.67)	0.08	0.05	0.0004	1.67 (1.29-2.15)	0.0031	1397
rs4841940	0.07	0.06	0.3264	1.19 (0.84-1.68)	0.08	0.06	0.0021	1.46 (1.14-1.87)	0.0401	2336
rs10906701	0.30	0.30	0.8259	0.98 (0.81-1.18)	0.32	0.28	0.0302	1.21 (1.05-1.39)	0.0016	3006
rs2056892	0.55	0.56	0.8885	0.99 (0.83-1.17)	0.59	0.55	0.0086	1.18 (1.04-1.35)	0.0002	3477
rs10500802	0.29	0.27	0.2383	1.12 (0.93-1.35)	0.32	0.27	0.0002	1.28 (1.11-1.47)	0.0065	1770
rs7927729	0.89	0.86	0.0282	1.33 (1.03-1.73)	0.90	0.85	0.0001	1.55 (1.26-1.91)	0.1109	1152
rs2186564	0.22	0.17	0.0008	1.37 (1.14-1.65)	0.23	0.17	2.80×10^{-7}	1.50 (1.29-1.74)	0.1307	855
rs2450135	0.47	0.46	0.6653	1.05 (0.88-1.24)	0.46	0.38	0.0071	1.41 (1.23-1.60)	0.0060	797
rs10892996	0.44	0.44	0.9757	1.00 (0.85-1.19)	0.48	0.44	0.0224	1.16 (1.02-1.32)	0.0038	4222
rs1629029	0.18	0.20	0.2655	0.88 (0.71-1.10)	0.22	0.19	0.0179	1.21 (1.04-1.42)	2.69×10^{-6}	3851
rs2641530	0.42	0.40	0.3262	1.09 (0.92-1.30)	0.44	0.39	0.0052	1.23 (1.08-1.40)	0.0476	2215
rs10459263	0.55	0.51	0.1323	1.14 (0.96-1.35)	0.57	0.51	0.0004	1.26 (1.10-1.44)	0.0374	1755
rs11110393	0.16	0.18	0.1261	0.84 (0.67-1.05)	0.19	0.19	0.3057	1.05 (0.89-1.24)	0.0002	61897
rs12317930	0.15	0.18	0.0290	0.77 (0.61-0.97)	0.18	0.18	0.5885	1.03 (0.87-1.22)	2.08×10^{-5}	177054
rs12430111	0.36	0.31	0.0326	1.22 (1.02-1.45)	0.38	0.31	1.45×10^{-5}	1.34 (1.17-1.54)	0.0452	1173
rs1336679	0.56	0.52	0.1068	1.16 (0.98-1.37)	0.57	0.51	0.0008	1.27 (1.12-1.45)	0.1070	1642
rs7338279	0.72	0.72	0.7423	1.03 (0.85-1.25)	0.74	0.71	0.0265	1.20 (1.03-1.39)	0.0171	3551
rs9601390	0.12	0.12	0.8926	1.02 (0.79-1.32)	0.14	0.12	0.0614	1.19 (0.99-1.44)	0.0380	6672
rs8000037	0.49	0.48	0.9388	1.01 (0.85-1.19)	0.51	0.48	0.0349	1.15 (1.01-1.31)	0.0089	4734
rs966724	0.16	0.16	0.8087	1.03 (0.81-1.29)	0.19	0.17	0.0074	1.18 (1.00-1.39)	0.0055	5597
rs9555548	0.89	0.88	0.8857	1.02 (0.78-1.33)	0.90	0.87	0.0154	1.36 (1.10-1.69)	0.0028	2498
rs8013963	0.07	0.09	0.1158	0.77 (0.56-1.07)	0.08	0.07	0.4550	1.21 (0.95-1.53)	0.0001	8832
rs917607	0.02	0.02	0.2253	0.67 (0.35-1.30)	0.03	0.02	0.0801	1.54 (1.01-2.34)	5.69×10^{-5}	4892
rs72683475	0.02	0.02	0.4212	0.77 (0.42-1.43)	0.03	0.02	0.1322	1.34 (0.90-2.01)	0.0030	11563
rs78647847	0.89	0.87	0.2253	1.18 (0.91-1.53)	0.89	0.86	0.0048	1.40 (1.14-1.73)	0.0812	1980
rs1746586	0.76	0.77	0.2759	0.90 (0.73-1.10)	0.77	0.75	0.3325	1.12 (0.96-1.31)	0.0033	10239
rs4886647	0.50	0.48	0.5743	1.05 (0.89-1.24)	0.52	0.48	0.0068	1.19 (1.05-1.36)	0.0101	3061
rs4779128	0.15	0.14	0.6601	1.06 (0.83-1.34)	0.17	0.14	0.0121	1.27 (1.06-1.51)	0.0163	3030
rs12597065	0.22	0.24	0.4508	0.93 (0.76-1.13)	0.25	0.23	0.0528	1.17 (1.00-1.35)	0.0003	5033
rs7194974	0.71	0.73	0.3932	0.92 (0.76-1.11)	0.74	0.71	0.1798	1.12 (0.97-1.30)	0.0016	9214
rs4790838	0.37	0.36	0.4739	1.07 (0.90-1.27)	0.40	0.36	0.0029	1.22 (1.07-1.39)	0.0091	2462
rs2013260	0.63	0.63	0.8355	0.98 (0.82-1.17)	0.66	0.64	0.0937	1.09 (0.95-1.25)	0.0100	13716
rs623011	0.63	0.46	5.33×10^{-15}	2.00 (1.69-2.38)	0.63	0.46	1.33×10^{-22}	1.96 (1.72-2.24)	0.7715	215
rs17714860	0.87	0.80	6.27×10^{-5}	1.63 (1.28-2.07)	0.87	0.79	2.31×10^{-8}	1.74 (1.44-2.10)	0.4434	590
rs411079	0.77	0.67	2.11×10^{-7}	1.71 (1.40-2.09)	0.77	0.67	1.26×10^{-10}	1.64 (1.40-1.91)	0.5380	497
rs7222503	0.89	0.83	2.02×10^{-5}	1.76 (1.35-2.29)	0.89	0.82	6.17×10^{-8}	1.79 (1.45-2.20)	0.9328	624
rs312729	0.65	0.47	8.75×10^{-20}	2.11 (1.79-2.48)	0.65	0.47	8.02×10^{-29}	2.08 (1.83-2.38)	0.8325	182
rs312691	0.63	0.46	1.31×10^{-14}	1.99 (1.67-2.37)	0.63	0.46	6.08×10^{-24}	2.02 (1.77-2.31)	0.7888	196
rs12451295	0.6	0.48	1.32×10^{-8}	1.63 (1.38-1.94)	0.61	0.49	2.41×10^{-13}	1.61 (1.41-1.84)	0.7743	422
rs16975694	0.76	0.69	0.0003	1.43 (1.18-1.74)	0.77	0.69	1.30×10^{-6}	1.45 (1.25-1.69)	0.6925	886
rs16975792	0.66	0.57	2.42×10^{-5}	1.46 (1.22-1.73)	0.66	0.57	4.04×10^{-8}	1.46 (1.27-1.67)	0.7892	700
rs1605750	0.75	0.69	0.0036	1.32 (1.09-1.60)	0.75	0.69	4.17×10^{-5}	1.37 (1.18-1.59)	0.4690	1212
rs62069783	0.67	0.60	0.0007	1.34 (1.13-1.60)	0.67	0.60	6.41×10^{-6}	1.37 (1.19-1.57)	0.6098	1035
rs2109051	0.9	0.87	0.0559	1.30 (0.99-1.70)	0.91	0.88	0.0024	1.33 (1.06-1.66)	0.2568	3340
rs2713969	0.38	0.38	0.8425	0.98 (0.83-1.17)	0.41	0.37	0.0532	1.14 (1.00-1.31)	0.0051	5613
rs4595868	0.22	0.20	0.1475	1.17 (0.95-1.43)	0.24	0.19	0.0004	1.35 (1.16-1.58)	0.0349	1445
rs7235371	0.47	0.43	0.0649	1.18 (0.99-1.39)	0.49	0.42	8.92E-05	1.31 (1.15-1.49)	0.0409	1275
rs2423166	0.14	0.14	0.9971	1.00 (0.78-1.28)	0.16	0.13	0.0338	1.24 (1.03-1.48)	0.0081	4010
rs4239695	0.26	0.28	0.2106	0.88 (0.73-1.07)	0.29	0.27	0.3444	1.07 (0.92-1.23)	0.0012	24586
rs1570530	0.04	0.06	0.0496	0.67 (0.44-1.01)	0.06	0.05	0.5206	1.12 (0.85-1.49)	7.00×10^{-5}	35556
rs1993668	0.05	0.05	0.9911	1.00 (0.69-1.45)	0.07	0.06	0.0293	1.27 (0.98-1.64)	0.0150	6302
rs133501	0.53	0.53	0.8983	0.99 (0.83-1.17)	0.56	0.53	0.0357	1.13 (0.99-1.28)	0.0030	6276

© 2019 Zhao S-X et al. *JAMA Network Open.*

**Continued eTable 2**								
SNP	maleTPPvsmaleControl (455 vs 1,648)				TPPvsGD (533 vs 1,404)			
	F_TPP	F_GD	*P*-value	OR (95% CI)	F_TPP	F_GD	*P*-value	OR (95% CI)
rs6667605	0.58	0.52	0.0046	1.23 (1.08-1.45)	0.57	0.55	0.3266	1.07 (0.93-1.24)
rs16831558	0.39	0.32	0.0002	1.35 (1.16-1.57)	0.38	0.35	0.0949	1.13 (0.98-1.31)
rs1007795	0.60	0.56	0.0328	1.18 (1.02-1.37)	0.60	0.57	0.1096	1.13 (0.97-1.30)
rs1146530	0.83	0.79	0.0052	1.32 (1.09-1.61)	0.83	0.80	0.0469	1.21 (1.01-1.46)
rs1144303	0.77	0.72	0.0007	1.35 (1.14-1.59)	0.77	0.73	0.0089	1.25 (1.06-1.48)
rs539799	0.13	0.12	0.5835	1.06 (0.85-1.33)	0.12	0.12	0.6328	1.05 (0.85-1.31)
rs4271175	0.12	0.09	0.0046	1.39 (1.10-1.76)	0.12	0.10	0.0224	1.30 (1.04-1.62)
rs7529077	0.03	0.02	0.0412	1.56 (1.02-2.39)	0.03	0.02	0.1357	1.39 (0.90-2.14)
rs13400534	0.68	0.63	0.0031	1.27 (1.09-1.49)	0.69	0.65	0.0119	1.21 (1.04-1.41)
rs6751710	0.64	0.59	0.0069	1.23 (1.06-1.45)	0.63	0.58	0.0045	1.23 (1.07-1.41)
rs6806226	0.16	0.12	0.0031	1.35 (1.10-1.66)	0.15	0.14	0.4401	1.08 (0.89-1.32)
rs16835753	0.43	0.41	0.2320	1.10 (0.94-1.27)	0.42	0.41	0.4950	1.05 (0.91-1.21)
rs10026043	0.92	0.90	0.0851	1.27 (0.97-1.64)	0.92	0.90	0.0647	1.26 (0.98-1.62)
rs10022828	0.05	0.03	0.0020	1.76 (1.22-2.56)	0.04	0.03	0.0067	1.68 (1.15-2.46)
rs28692179	0.35	0.31	0.0323	1.19 (1.02-1.38)	0.35	0.32	0.1015	1.13 (0.98-1.31)
rs12511622	0.34	0.37	0.1624	0.89 (0.77-1.05)	0.34	0.35	0.8252	0.98 (0.85-1.14)
rs1352714	0.20	0.13	5.48×10^{-8}	1.65 (1.38-1.99)	0.20	0.15	7.01×10^{-6}	1.46 (1.24-1.72)
rs17031489	0.61	0.56	0.0087	1.22 (1.05-1.43)	0.60	0.55	0.0025	1.24 (1.08-1.44)
rs7681004	0.08	0.05	0.0004	1.73 (1.27-2.35)	0.08	0.05	0.0002	1.64 (1.25-2.14)
rs9312559	0.73	0.72	0.6231	1.04 (0.88-1.23)	0.73	0.73	0.9978	1.00 (0.85-1.17)
rs6860373	0.04	0.02	0.0180	1.65 (1.09-2.48)	0.04	0.03	0.2352	1.27 (0.86-1.86)
rs6450743	0.03	0.02	0.0083	1.84 (1.17-2.89)	0.03	0.02	0.0282	1.63 (1.06-2.51)
rs9292371	0.04	0.03	0.5084	1.15 (0.77-1.73)	0.04	0.02	0.0061	1.83 (1.20-2.79)
rs271245	0.56	0.56	0.9629	1.00 (0.86-1.16)	0.56	0.56	0.9242	1.01 (0.87-1.16)
rs697098	0.47	0.45	0.2632	1.09 (0.94-1.26)	0.46	0.43	0.0398	1.15 (1.01-1.32)
rs7721254	0.09	0.08	0.3272	1.14 (0.88-1.49)	0.09	0.07	0.1193	1.23 (0.95-1.59)
rs6881055	0.19	0.16	0.0482	1.21 (1.00-1.47)	0.19	0.16	0.0523	1.21 (1.00-1.45)
rs59538022	0.29	0.29	0.9683	1.00 (0.85-1.17)	0.29	0.28	0.6337	1.04 (0.89-1.21)
rs2070843	0.75	0.72	0.0621	1.18 (0.99-1.39)	0.75	0.73	0.3666	1.08 (0.92-1.26)
rs10039049	0.50	0.45	0.0148	1.20 (1.04-1.39)	0.49	0.47	0.5016	1.05 (0.91-1.21)
rs12529334	0.93	0.91	0.0066	1.49 (1.12-2.00)	0.94	0.92	0.0441	1.33 (1.01-1.76)
rs61748643	0.04	0.02	8.82×10^{-5}	2.27 (1.49-3.46)	0.04	0.02	0.0057	1.74 (1.17-2.59)
rs9471082	0.89	0.87	0.3171	1.12 (0.89-1.41)	0.88	0.87	0.6445	1.05 (0.85-1.31)

© 2019 Zhao S-X et al. *JAMA Network Open.*

rs952880	0.26	0.23	0.0281	1.21 (1.02-1.43)	0.26	0.24	0.2420	1.10 (0.94-1.30)
rs9485478	0.89	0.86	0.0512	1.25 (1.00-1.56)	0.88	0.86	0.0888	1.21 (0.97-1.50)
rs77500196	0.89	0.86	0.0385	1.27 (1.01-1.61)	0.89	0.87	0.0987	1.20 (0.97-1.50)
rs487697	0.31	0.27	0.0262	1.20 (1.02-1.41)	0.32	0.28	0.0331	1.18 (1.01-1.38)
rs2356183	0.37	0.32	0.0007	1.30 (1.12-1.51)	0.37	0.32	0.0019	1.24 (1.08-1.41)
rs12194555	0.38	0.31	7.49×10^{-5}	1.37 (1.17-1.59)	0.37	0.33	0.0048	1.23 (1.06-1.43)
rs541442	0.48	0.41	0.0002	1.31 (1.14-1.52)	0.47	0.42	0.0015	1.23 (1.08-1.40)
rs6569026	0.43	0.37	0.0009	1.29 (1.11-1.51)	0.42	0.36	0.0012	1.27 (1.10-1.46)
rs2740761	0.08	0.06	0.0842	1.28 (0.97-1.69)	0.07	0.07	0.8136	1.03 (0.79-1.35)
rs76087603	0.06	0.03	4.66×10^{-5}	2.04 (1.44-2.89)	0.05	0.04	0.0372	1.41 (1.02-1.95)
rs16876379	0.11	0.08	0.0124	1.38 (1.08-1.77)	0.10	0.09	0.2517	1.15 (0.91-1.45)
rs62513232	0.07	0.04	0.0007	1.71 (1.25-2.35)	0.06	0.04	0.0160	1.46 (1.07-1.98)
rs10099843	0.07	0.04	0.0038	1.56 (1.15-2.12)	0.07	0.05	0.0513	1.34 (1.00-1.81)
rs4741926	0.32	0.28	0.0118	1.23 (1.05-1.45)	0.32	0.28	0.0245	1.19 (1.02-1.39)
rs10756531	0.62	0.61	0.3440	1.08 (0.93-1.25)	0.62	0.59	0.1831	1.10 (0.96-1.28)
rs60798663	0.08	0.05	0.0013	1.59 (1.20-2.11)	0.08	0.05	0.0002	1.68 (1.27-2.21)
rs4841940	0.08	0.06	0.0073	1.46 (1.11-1.93)	0.08	0.06	0.0081	1.42 (1.09-1.85)
rs10906701	0.32	0.29	0.0561	1.17 (0.99-1.37)	0.32	0.29	0.0500	1.17 (1.00-1.36)
rs2056892	0.60	0.56	0.0146	1.20 (1.04-1.41)	0.59	0.58	0.3954	1.06 (0.92-1.22)
rs10500802	0.32	0.27	0.0011	1.30 (1.11-1.53)	0.33	0.28	0.0064	1.23 (1.06-1.42)
rs7927729	0.90	0.86	0.0014	1.45 (1.15-1.85)	0.90	0.86	0.0030	1.39 (1.12-1.73)
rs2186564	0.22	0.18	0.0010	1.34 (1.13-1.60)	0.23	0.18	0.0005	1.32 (1.13-1.54)
rs2450135	0.47	0.41	0.0075	1.26 (1.09-1.46)	0.46	0.36	1.15×10^{-7}	1.51 (1.31-1.74)
rs10892996	0.48	0.44	0.0250	1.19 (1.02-1.37)	0.48	0.45	0.2252	1.09 (0.95-1.26)
rs1629029	0.22	0.19	0.0376	1.21 (1.01-1.45)	0.22	0.20	0.3179	1.09 (0.92-1.30)
rs2641530	0.42	0.39	0.0638	1.15 (0.99-1.34)	0.43	0.39	0.0303	1.18 (1.02-1.36)
rs10459263	0.57	0.51	0.0019	1.27 (1.09-1.47)	0.57	0.52	0.0026	1.24 (1.08-1.43)
rs11110393	0.19	0.18	0.3726	1.09 (0.90-1.31)	0.19	0.18	0.5144	1.06 (0.89-1.27)
rs12317930	0.18	0.18	0.5996	1.05 (0.87-1.28)	0.18	0.17	0.5043	1.07 (0.88-1.28)
rs12430111	0.38	0.32	0.0003	1.32 (1.14-1.54)	0.38	0.33	0.0086	1.22 (1.05-1.41)
rs1336679	0.56	0.51	0.0068	1.23 (1.06-1.43)	0.57	0.53	0.0334	1.17 (1.01-1.35)
rs7338279	0.74	0.71	0.1498	1.14 (0.95-1.33)	0.74	0.71	0.0417	1.18 (1.01-1.39)
rs9601390	0.14	0.12	0.0750	1.22 (0.98-1.51)	0.14	0.13	0.5555	1.06 (0.87-1.31)
rs8000037	0.51	0.48	0.1103	1.13 (0.97-1.31)	0.51	0.50	0.4322	1.06 (0.92-1.22)
rs966724	0.19	0.16	0.0062	1.28 (1.06-1.55)	0.19	0.19	0.9819	1.00 (0.83-1.19)
rs9555548	0.90	0.87	0.0113	1.37 (1.08-1.72)	0.90	0.87	0.0085	1.35 (1.08-1.69)

© 2019 Zhao S-X et al. *JAMA Network Open.*

rs8013963	0.08	0.07	0.7704	1.04 (0.79-1.37)	0.08	0.06	0.0356	1.33 (1.02-1.73)
rs917607	0.03	0.02	0.0944	1.49 (0.93-2.39)	0.03	0.02	0.0212	1.72 (1.09-2.73)
rs72683475	0.03	0.02	0.2845	1.28 (0.82-2.00)	0.03	0.02	0.4766	1.18 (0.76-1.82)
rs78647847	0.90	0.86	0.0030	1.43 (1.14-1.82)	0.89	0.87	0.0888	1.22 (0.97-1.53)
rs1746586	0.78	0.76	0.4242	1.08 (0.90-1.28)	0.77	0.75	0.2045	1.11 (0.94-1.32)
rs4886647	0.52	0.48	0.0145	1.20 (1.04-1.40)	0.52	0.51	0.4282	1.06 (0.92-1.22)
rs4779128	0.17	0.13	0.0017	1.38 (1.13-1.68)	0.17	0.15	0.1076	1.17 (0.96-1.42)
rs12597065	0.26	0.23	0.0833	1.16 (0.98-1.38)	0.26	0.24	0.4236	1.07 (0.91-1.26)
rs7194974	0.73	0.71	0.3960	1.08 (0.91-1.27)	0.74	0.71	0.1329	1.13 (0.96-1.33)
rs4790838	0.41	0.36	0.0070	1.23 (1.06-1.43)	0.40	0.37	0.0772	1.14 (0.99-1.32)
rs2013260	0.66	0.64	0.2363	1.10 (0.94-1.28)	0.66	0.67	0.7441	0.97 (0.84-1.13)
rs623011	0.66	0.47	9.00×10^{-22}	2.19 (1.87-2.57)	0.63	0.46	4.95×10^{-20}	1.96 (1.70-2.26)
rs17714860	0.88	0.80	6.87×10^{-8}	1.85 (1.47-2.33)	0.87	0.80	1.25×10^{-7}	1.71 (1.40-2.08)
rs411079	0.79	0.67	9.04×10^{-12}	1.89 (1.59-2.27)	0.77	0.67	5.24×10^{-11}	1.69 (1.44-1.99)
rs7222503	0.90	0.83	6.17×10^{-7}	1.85 (1.45-2.38)	0.89	0.83	8.04×10^{-7}	1.72 (1.38-2.13)
rs312729	0.68	0.48	3.12×10^{-27}	2.33 (2.00-2.70)	0.65	0.47	3.12×10^{-27}	2.10 (1.84-2.40)
rs312691	0.66	0.47	3.48×10^{-22}	2.22 (1.89-2.56)	0.63	0.45	7.02×10^{-24}	2.08 (1.81-2.39)
rs12451295	0.63	0.50	1.57×10^{-11}	1.72 (1.47-2.00)	0.61	0.48	3.91×10^{-12}	1.64 (1.43-1.89)
rs16975694	0.79	0.70	6.03×10^{-7}	1.59 (1.32-1.89)	0.77	0.69	3.59×10^{-7}	1.50 (1.28-1.76)
rs16975792	0.68	0.58	7.50×10^{-8}	1.54 (1.32-1.82)	0.66	0.56	2.81×10^{-8}	1.50 (1.30-1.73)
rs1605750	0.77	0.69	2.06×10^{-6}	1.52 (1.28-1.82)	0.75	0.68	2.16×10^{-5}	1.40 (1.19-1.64)
rs62069783	0.69	0.60	1.31×10^{-6}	1.47 (1.25-1.72)	0.67	0.60	1.33×10^{-5}	1.37 (1.19-1.59)
rs2109051	0.92	0.88	0.0056	1.45 (1.11-1.89)	0.91	0.88	0.0158	1.34 (1.06-1.70)
rs2713969	0.42	0.38	0.0385	1.17 (1.01-1.36)	0.40	0.38	0.2647	1.09 (0.94-1.26)
rs4595868	0.23	0.19	0.0025	1.32 (1.11-1.57)	0.24	0.20	0.0031	1.28 (1.09-1.51)
rs7235371	0.49	0.43	0.0013	1.28 (1.11-1.49)	0.49	0.42	0.0004	1.29 (1.12-1.48)
rs2423166	0.16	0.14	0.1622	1.16 (0.94-1.41)	0.16	0.13	0.0394	1.24 (1.01-1.51)
rs4239695	0.29	0.27	0.1834	1.12 (0.95-1.32)	0.28	0.29	0.9842	1.00 (0.85-1.17)
rs1570530	0.06	0.06	0.7659	1.05 (0.77-1.44)	0.06	0.06	0.6781	0.94 (0.69-1.27)
rs1993668	0.07	0.05	0.0280	1.39 (1.04-1.87)	0.07	0.06	0.1561	1.23 (0.92-1.63)
rs133501	0.56	0.53	0.0975	1.14 (0.98-1.32)	0.56	0.53	0.0882	1.13 (0.98-1.31)

© 2019 Zhao S-X et al. *JAMA Network Open.*

Continued eTable 2										
SNP	maleTPPvsmaleGD (455 vs 315)				GD vs Controls (1,404 vs 3,249)				F_CHB	F_EUR
	F_TPP	F_GD	*P*-value	OR (95% CI)	F_GD	F_Cons	*P*-value	OR (95% CI)		
rs6667605	0.58	0.55	0.3237	1.11 (0.90-1.37)	0.55	0.52	0.0045	1.14 (1.04-1.24)	0.52	0.50
rs16831558	0.39	0.38	0.7875	1.03 (0.83-1.27)	0.35	0.33	0.0194	1.12 (1.02-1.23)	0.33	0.19
rs1007795	0.60	0.60	0.8388	1.02 (0.83-1.27)	0.57	0.56	0.2571	1.05 (0.96-1.15)	0.56	0.52
rs1146530	0.83	0.78	0.0092	1.43 (1.10-1.85)	0.80	0.79	0.1268	1.09 (0.98-1.22)	0.79	0.91
rs1144303	0.77	0.71	0.0098	1.37 (1.09-1.75)	0.73	0.72	0.2238	1.06 (0.96-1.17)	0.72	0.74
rs539799	0.13	0.10	0.1321	1.28 (0.93-1.78)	0.12	0.12	0.9604	1.00 (0.87-1.14)	0.12	0.46
rs4271175	0.12	0.09	0.0957	1.33 (0.95-1.86)	0.10	0.09	0.2745	1.09 (0.94-1.27)	0.09	0.21
rs7529077	0.03	0.02	0.1789	1.55 (0.82-2.94)	0.02	0.02	0.5177	1.11 (0.81-1.50)	0.02	0.20
rs13400534	0.68	0.64	0.0410	1.20 (1.00-1.43)	0.65	0.62	0.0313	1.11 (1.01-1.22)	0.62	0.56
rs6751710	0.64	0.59	0.0413	1.20 (1.01-1.43)	0.58	0.58	0.9296	1.00 (0.92-1.09)	0.58	0.61
rs6806226	0.16	0.14	0.3483	1.15 (0.86-1.53)	0.14	0.12	0.0141	1.17 (1.03-1.34)	0.12	0.10
rs16835753	0.43	0.39	0.1427	1.17 (0.95-1.44)	0.41	0.41	0.7495	1.02 (0.93-1.11)	0.41	0.58
rs10026043	0.92	0.89	0.0218	1.41 (1.05-1.89)	0.90	0.90	0.6235	1.04 (0.90-1.19)	0.90	0.99
rs10022828	0.05	0.03	0.0363	1.85 (1.03-3.32)	0.03	0.02	0.6636	1.07 (0.80-1.41)	0.02	0.02
rs28692179	0.35	0.34	0.6011	1.06 (0.85-1.31)	0.32	0.32	0.5912	1.03 (0.93-1.13)	0.32	0.11
rs12511622	0.34	0.37	0.2590	0.90 (0.76-1.08)	0.35	0.35	0.6842	0.98 (0.90-1.07)	0.35	0.25
rs1352714	0.20	0.16	0.0099	1.32 (1.07-1.64)	0.15	0.14	0.0921	1.08 (0.99-1.17)	0.14	0.03
rs17031489	0.61	0.57	0.1133	1.18 (0.95-1.45)	0.55	0.56	0.4473	0.97 (0.88-1.06)	0.56	0.68
rs7681004	0.08	0.05	0.0090	1.58 (1.11-2.24)	0.05	0.05	0.7537	1.03 (0.85-1.24)	0.05	0.01
rs9312559	0.73	0.74	0.6669	0.95 (0.75-1.20)	0.73	0.71	0.1144	1.08 (0.98-1.20)	0.71	0.36
rs6860373	0.04	0.03	0.6854	1.12 (0.65-1.96)	0.03	0.02	0.0832	1.27 (0.97-1.66)	0.02	0.30
rs6450743	0.03	0.02	0.2824	1.44 (0.75-2.75)	0.02	0.02	0.5208	1.11 (0.81-1.53)	0.02	0.04
rs9292371	0.04	0.02	0.2096	1.50 (0.81-2.80)	0.02	0.02	0.1928	0.81 (0.59-1.10)	0.02	0.06
rs271245	0.56	0.58	0.5949	0.94 (0.77-1.16)	0.56	0.55	0.4910	1.03 (0.94-1.13)	0.55	0.98
rs697098	0.47	0.43	0.0484	1.18 (1.00-1.40)	0.43	0.43	0.7501	0.99 (0.91-1.07)	0.43	0.53
rs7721254	0.09	0.09	0.7949	0.95 (0.66-1.36)	0.07	0.07	0.7520	1.03 (0.87-1.22)	0.07	0.08
rs6881055	0.19	0.14	0.0111	1.44 (1.08-1.90)	0.16	0.16	0.9702	1.00 (0.89-1.13)	0.16	0.39
rs59538022	0.29	0.30	0.7086	0.96 (0.77-1.20)	0.28	0.29	0.7815	0.99 (0.89-1.09)	0.29	0.03
rs2070843	0.75	0.75	0.8924	0.98 (0.78-1.25)	0.73	0.72	0.4013	1.04 (0.95-1.15)	0.72	0.99
rs10039049	0.50	0.50	0.8633	0.98 (0.80-1.21)	0.47	0.44	0.0070	1.13 (1.03-1.23)	0.44	0.67
rs12529334	0.93	0.91	0.0614	1.45 (0.99-2.13)	0.92	0.91	0.1729	1.12 (0.96-1.31)	0.91	0.79
rs61748643	0.04	0.02	0.0105	1.90 (1.15-3.11)	0.02	0.02	0.1534	1.25 (0.92-1.71)	0.02	0.01

© 2019 Zhao S-X et al. *JAMA Network Open.*

rs9471082	0.88	0.88	0.7867	1.04 (0.76-1.43)	0.87	0.87	0.7952	1.02 (0.89-1.16)	0.87	0.81
rs952880	0.26	0.23	0.1429	1.19 (0.94-1.51)	0.24	0.23	0.2361	1.07 (0.96-1.18)	0.23	0.42
rs9485478	0.89	0.86	0.1384	1.27 (0.93-1.72)	0.86	0.86	0.6382	1.03 (0.91-1.17)	0.86	0.97
rs77500196	0.89	0.86	0.1447	1.27 (0.93-1.72)	0.87	0.86	0.6294	1.03 (0.91-1.18)	0.86	0.98
rs487697	0.31	0.33	0.4599	0.92 (0.74-1.15)	0.28	0.27	0.1951	1.07 (0.97-1.18)	0.27	0.32
rs2356183	0.37	0.30	0.0005	1.36 (1.14-1.63)	0.32	0.32	0.5151	1.02 (0.96-1.09)	0.32	0.47
rs12194555	0.37	0.30	0.0005	1.37 (1.15-1.64)	0.33	0.31	0.1832	1.07 (0.97-1.17)	0.31	0.48
rs541442	0.48	0.41	0.0006	1.34 (1.13-1.58)	0.42	0.41	0.4275	1.03 (0.96-1.09)	0.41	0.73
rs6569026	0.43	0.33	0.0002	1.50 (1.21-1.85)	0.36	0.37	0.6405	0.98 (0.89-1.07)	0.37	0.68
rs2740761	0.08	0.08	0.7936	0.95 (0.65-1.39)	0.07	0.06	0.0543	1.19 (1.00-1.42)	0.06	0.21
rs76087603	0.06	0.04	0.0317	1.55 (1.04-2.30)	0.04	0.03	0.0408	1.26 (1.01-1.58)	0.03	0.10
rs16876379	0.11	0.10	0.6554	1.08 (0.77-1.51)	0.09	0.08	0.0458	1.17 (1.00-1.37)	0.08	0.16
rs62513232	0.07	0.04	0.0134	1.84 (1.13-3.01)	0.04	0.04	0.4967	1.08 (0.87-1.34)	0.04	0.23
rs10099843	0.07	0.04	0.0163	1.77 (1.10-2.86)	0.05	0.05	0.4725	1.08 (0.88-1.32)	0.05	0.23
rs4741926	0.32	0.31	0.6683	1.05 (0.84-1.31)	0.28	0.27	0.3480	1.05 (0.95-1.16)	0.27	0.44
rs10756531	0.62	0.60	0.4392	1.09 (0.88-1.33)	0.59	0.60	0.7418	0.99 (0.90-1.08)	0.60	0.62
rs60798663	0.08	0.05	0.0006	1.84 (1.29-2.61)	0.05	0.05	0.9485	0.99 (0.82-1.21)	0.05	0.15
rs4841940	0.08	0.05	0.0073	1.58 (1.13-2.20)	0.06	0.06	0.7720	1.03 (0.86-1.23)	0.06	0.19
rs10906701	0.32	0.31	0.7364	1.04 (0.83-1.30)	0.29	0.28	0.4680	1.04 (0.94-1.14)	0.28	0.06
rs2056892	0.61	0.58	0.1934	1.12 (0.94-1.33)	0.58	0.55	0.0072	1.12 (1.03-1.22)	0.55	0.57
rs10500802	0.32	0.27	0.0038	1.31 (1.09-1.58)	0.28	0.27	0.3039	1.05 (0.96-1.15)	0.27	0.08
rs7927729	0.90	0.86	0.0086	1.43 (1.09-1.85)	0.86	0.85	0.0617	1.12 (0.99-1.26)	0.85	0.81
rs2186564	0.22	0.20	0.2521	1.13 (0.92-1.38)	0.18	0.17	0.0021	1.13 (1.05-1.22)	0.17	0.12
rs2450135	0.46	0.38	0.0017	1.44 (1.17-1.78)	0.36	0.38	0.1280	0.93 (0.85-1.02)	0.38	0.05
rs10892996	0.48	0.44	0.1039	1.19 (0.97-1.46)	0.45	0.44	0.2224	1.06 (0.97-1.16)	0.44	0.48
rs1629029	0.22	0.21	0.5096	1.09 (0.85-1.40)	0.20	0.19	0.0677	1.11 (0.99-1.24)	0.19	0.35
rs2641530	0.42	0.42	0.7602	1.03 (0.84-1.27)	0.39	0.39	0.4105	1.04 (0.95-1.14)	0.39	0.37
rs10459263	0.57	0.51	0.0198	1.28 (1.04-1.56)	0.52	0.51	0.7800	1.01 (0.93-1.11)	0.51	0.93
rs11110393	0.19	0.18	0.4208	1.11 (0.86-1.45)	0.18	0.19	0.7729	0.98 (0.88-1.10)	0.19	0.04
rs12317930	0.18	0.17	0.4804	1.10 (0.84-1.44)	0.17	0.18	0.5657	0.97 (0.86-1.09)	0.18	0.05
rs12430111	0.38	0.35	0.2361	1.11 (0.93-1.32)	0.33	0.31	0.0590	1.10 (1.00-1.21)	0.31	0.42
rs1336679	0.56	0.58	0.6201	0.94 (0.77-1.16)	0.53	0.51	0.0676	1.09 (1.00-1.19)	0.51	0.26
rs7338279	0.74	0.74	0.8860	1.02 (0.81-1.28)	0.71	0.71	0.8038	1.01 (0.92-1.12)	0.71	0.71
rs9601390	0.14	0.12	0.2259	1.21 (0.89-1.65)	0.13	0.12	0.0803	1.12 (0.99-1.28)	0.12	0.19
rs8000037	0.49	0.49	0.9264	1.01 (0.82-1.24)	0.50	0.48	0.0688	1.09 (0.99-1.19)	0.48	0.35
rs966724	0.19	0.18	0.5411	1.08 (0.83-1.41)	0.19	0.17	0.0036	1.18 (1.05-1.32)	0.17	0.38

© 2019 Zhao S-X et al. *JAMA Network Open.*

rs9555548	0.90	0.88	0.0926	1.27 (0.96-1.67)	0.87	0.87	0.8927	1.01 (0.89-1.14)	0.87	0.44
rs8013963	0.08	0.07	0.5843	1.11 (0.75-1.65)	0.06	0.07	0.3181	0.91 (0.76-1.09)	0.07	0.18
rs917607	0.03	0.01	0.0069	2.36 (1.26-4.45)	0.02	0.02	0.4917	0.89 (0.64-1.24)	0.02	0.11
rs72683475	0.03	0.01	0.0221	2.68 (1.16-6.20)	0.02	0.02	0.3666	1.15 (0.85-1.54)	0.02	0.11
rs78647847	0.90	0.87	0.1262	1.30 (0.94-1.79)	0.87	0.86	0.0415	1.15 (1.00-1.31)	0.86	0.95
rs1746586	0.78	0.75	0.2200	1.16 (0.92-1.49)	0.75	0.75	0.8859	1.01 (0.91-1.12)	0.75	0.76
rs4886647	0.48	0.50	0.5393	0.94 (0.76-1.15)	0.51	0.48	0.0120	1.12 (1.03-1.22)	0.48	0.12
rs4779128	0.17	0.14	0.0970	1.26 (0.95-1.68)	0.15	0.14	0.2794	1.07 (0.94-1.22)	0.14	0.26
rs12597065	0.26	0.22	0.0860	1.23 (0.97-1.57)	0.24	0.23	0.0801	1.10 (0.99-1.22)	0.23	0.63
rs7194974	0.73	0.70	0.3106	1.12 (0.89-1.41)	0.71	0.71	0.8132	0.99 (0.90-1.09)	0.71	0.79
rs4790838	0.41	0.39	0.6093	1.06 (0.86-1.30)	0.37	0.36	0.1841	1.06 (0.97-1.17)	0.36	0.31
rs2013260	0.66	0.66	0.9526	0.99 (0.80-1.23)	0.67	0.64	0.0192	1.12 (1.02-1.23)	0.64	0.92
rs623011	0.66	0.44	5.09×10^{-23}	2.50 (2.08-2.94)	0.46	0.46	0.9771	1.00 (0.92-1.09)	0.46	0.30
rs17714860	0.88	0.78	1.35×10^{-8}	2.04 (1.61-2.56)	0.80	0.79	0.7771	1.01 (0.92-1.12)	0.79	0.57
rs411079	0.79	0.66	3.68×10^{-12}	2.00 (1.64-2.44)	0.67	0.67	0.6475	0.98 (0.90-1.07)	0.67	0.75
rs7222503	0.90	0.82	1.86×10^{-7}	2.00 (1.56-2.63)	0.83	0.82	0.4752	1.04 (0.93-1.16)	0.82	0.63
rs312729	0.68	0.45	5.02×10^{-25}	2.50 (2.13-3.03)	0.47	0.47	0.8718	1.00 (0.94-1.06)	0.47	0.47
rs312691	0.66	0.43	1.90×10^{-25}	2.56 (2.17-3.03)	0.45	0.46	0.4918	0.97 (0.90-1.06)	0.46	0.28
rs12451295	0.63	0.48	1.57×10^{-11}	1.82 (1.52-2.13)	0.48	0.49	0.6093	0.98 (0.90-1.06)	0.49	0.35
rs16975694	0.79	0.69	4.01×10^{-7}	1.64 (1.35-2.00)	0.69	0.69	0.4574	0.97 (0.89-1.06)	0.69	0.73
rs16975792	0.68	0.56	6.68×10^{-8}	1.64 (1.37-1.96)	0.56	0.57	0.4648	0.97 (0.89-1.05)	0.57	0.31
rs1605750	0.77	0.68	1.89×10^{-6}	1.59 (1.32-1.92)	0.68	0.69	0.5737	0.98 (0.89-1.07)	0.69	0.55
rs62069783	0.69	0.59	4.51×10^{-6}	1.52 (1.27-1.82)	0.60	0.60	0.8251	0.99 (0.91-1.08)	0.60	0.22
rs2109051	0.92	0.88	0.0150	1.52 (1.09-2.13)	0.88	0.88	0.8021	0.98 (0.86-1.13)	0.88	0.69
rs2713969	0.42	0.40	0.6124	1.06 (0.86-1.30)	0.38	0.37	0.3337	1.05 (0.95-1.15)	0.37	0.71
rs4595868	0.23	0.19	0.0094	1.32 (1.08-1.62)	0.20	0.19	0.3387	1.05 (0.95-1.17)	0.19	0.22
rs7235371	0.49	0.41	0.0003	1.37 (1.16-1.63)	0.42	0.42	0.6916	1.02 (0.94-1.11)	0.42	0.52
rs2423166	0.16	0.13	0.1410	1.25 (0.93-1.67)	0.13	0.13	0.8610	0.99 (0.87-1.13)	0.13	0.19
rs4239695	0.29	0.27	0.4795	1.09 (0.87-1.36)	0.29	0.27	0.2690	1.06 (0.96-1.17)	0.27	0.53
rs1570530	0.06	0.07	0.5235	0.87 (0.57-1.32)	0.06	0.05	0.0523	1.21 (1.00-1.46)	0.05	0.52
rs1993668	0.07	0.07	0.9990	1.00 (0.67-1.48)	0.06	0.06	0.8505	1.02 (0.84-1.23)	0.06	0.51
rs133501	0.56	0.53	0.3345	1.11 (0.90-1.37)	0.53	0.53	0.7845	0.99 (0.90-1.08)	0.53	0.20

© 2019 Zhao S-X et al. *JAMA Network Open.*

eTable 3. Association results with TPP in the initial genome-wide scan and the second-stage analysis for the 34 GD risk SNPs												
Chr.	SNP	BP	Annotated Genes	Alleles	TPPvsControl Discovery (171 vs 2,160)				TPPvsControl Replication (362 vs 1,089)			
					F_TPP	F_Cons	*P*-value	OR (95% CI)	F_TPP	F_Cons	*P*-value	OR (95% CI)
1	rs2843403	2529097	*MMEL1*	C/T	0.61	0.51	0.0003	1.52 (1.21-1.90)	0.54	0.54	0.7879	1.02 (0.86-1.21)
1	rs11590198	2551900	*MMEL1*	G/A	0.64	0.53	0.0002	1.55 (1.23-1.94)	0.57	0.56	0.7116	1.03 (0.87-1.22)
1	rs4648661	2568425	*MMEL1/TTC34*	C/T	0.53	0.43	0.0001	1.53 (1.23-1.91)	0.47	0.44	0.1733	1.12 (0.95-1.33)
1	rs3761959	157669278	*FCRL3*	T/C	0.45	0.40	0.0448	1.25 (1.01-1.56)	0.47	0.41	0.0013	1.29 (1.10-1.50)
1	rs1265883	160464911	*SLAMF6*	C/A	0.13	0.11	0.2339	1.22 (0.88-1.70)	0.13	0.09	0.0061	1.38 (1.10-1.73)
2	rs1881145	12634278	*TRIB2/LPIN1*	A/T	0.68	0.60	0.0020	1.47 (1.15-1.86)	0.67	0.60	0.0006	1.35 (1.14-1.60)
2	rs1534422	12640741	*TRIB2/LPIN1*	G/A	0.27	0.24	0.2373	1.16 (0.91-1.49)	0.25	0.24	0.5654	1.06 (0.87-1.29)
2	rs4669857	12658305	*TRIB2/LPIN1*	A/T	0.62	0.58	0.1581	1.18 (0.94-1.48)	0.61	0.58	0.1606	1.12 (0.95-1.32)
2	rs1024161	204721752	*CTLA4/CD28*	T/C	0.76	0.67	0.0002	1.61 (1.25-2.09)	0.73	0.69	0.0478	1.19 (1.00-1.41)
3	rs13093110	188125120	*LPP*	C/T	0.40	0.39	0.8287	1.03 (0.82-1.28)	0.35	0.40	0.0113	0.81 (0.69-0.95)
3	rs6806091	188237765	*LPP*	C/G	0.19	0.18	0.6106	1.07 (0.81-1.42)	0.21	0.18	0.1208	1.17 (0.96-1.43)
4	rs6832151	40303633	*CHRNA9/RHOH*	G/T	0.45	0.35	0.0003	1.51 (1.21-1.88)	0.40	0.35	0.0112	1.22 (1.05-1.43)
5	rs1368408	147258162	*SCGB3A2/SPINK1*	A/G	0.21	0.20	0.5407	1.09 (0.83-1.42)	0.19	0.19	0.7122	1.04 (0.84-1.29)
6	**rs4947296**	31058178	*C6orf15/PBMUCL1*	C/T	0.27	0.15	1.99×10^{-8}	2.07 (1.61-2.66)	0.25	0.14	1.38×10^{-13}	2.05 (1.70-2.46)
6	**rs1521**	31350704	*MICA/HLA-B*	T/C	0.90	0.83	0.0013	1.80 (1.27-2.57)	0.92	0.79	4.36×10^{-16}	3.04 (2.30-4.00)
6	rs6903608	32428285	*HLA-DRA/HLA-DRB5*	C/T	0.37	0.37	0.8766	1.02 (0.81-1.28)	0.44	0.38	0.0027	1.27 (1.09-1.49)
6	**rs6457617**	32663851	*HLA-DQB1/HLA-DQA2*	T/C	0.64	0.49	1.55×10^{-7}	1.84 (1.47-2.31)	0.58	0.45	4.35×10^{-11}	1.69 (1.44-1.97)
6	**rs2281388**	33060118	*HLA-DPB1/COL11A2*	A/G	0.48	0.33	3.80×10^{-8}	1.82 (1.46-2.27)	0.46	0.33	4.00×10^{-12}	1.71 (1.47-1.99)
6	rs2474619	90880035	*BACH2*	A/C	0.66	0.62	0.1954	1.17 (0.93-1.47)	0.69	0.54	0.0119	1.88 (1.59-2.21)
6	rs9355610	167383075	*RNASET2/FGFR1OP*	G/A	0.55	0.47	0.0019	1.41 (1.13-1.76)	0.51	0.47	0.0275	1.19 (1.02-1.39)
8	rs2294025	134145512	*TG*	A/G	0.22	0.18	0.0504	1.30 (1.00-1.70)	0.22	0.20	0.1175	1.16 (0.96-1.39)
8	rs4736437	134185668	*WISP1/SLA*	G/T	0.22	0.19	0.1161	1.23 (0.95-1.61)	0.23	0.21	0.2201	1.12 (0.94-1.34)
9	rs505922	136149229	*ABO*	T/C	0.55	0.53	0.3858	1.10 (0.88-1.38)	0.59	0.54	0.0057	1.24 (1.07-1.45)
11	rs12575636	95311260	*FAM76B/SESN3*	G/T	0.12	0.09	0.1319	1.31 (0.92-1.85)	0.11	0.08	0.0158	1.38 (1.06-1.78)
11	rs4409785	95311422	*FAM76B/SESN3*	C/T	0.12	0.09	0.0855	1.35 (0.96-1.90)	0.11	0.09	0.0619	1.30 (0.99-1.72)
12	rs4768412	42869140	*PRICKLE1*	T/C	0.30	0.23	0.0092	1.39 (1.09-1.77)	0.24	0.22	0.4834	1.07 (0.88-1.31)
14	rs12101261	81451229	*TSHR*	T/C	0.66	0.64	0.3663	1.11 (0.88-1.40)	0.70	0.64	0.0009	1.32 (1.12-1.56)
14	rs1456988	98488007	*C14orf177/VRK1*	G/T	0.59	0.53	0.0283	1.28 (1.02-1.59)	0.54	0.53	0.7502	1.02 (0.88-1.19)
16	rs57348955	31185882	*FUS/PRSS36*	G/A	0.22	0.21	0.7188	1.05 (0.80-1.39)	0.23	0.20	0.1243	1.18 (0.96-1.44)
16	rs4423435	31560603	*AHSP/ZNF720*	G/A	0.54	0.52	0.3930	1.10 (0.88-1.38)	0.55	0.54	0.5818	1.05 (0.89-1.23)
20	rs1883832	44746982	*CD40*	C/T	0.67	0.64	0.2474	1.15 (0.91-1.45)	0.68	0.64	0.0499	1.18 (1.00-1.39)
22	rs229527	37581485	*C1QTNF6*	A/C	0.77	0.70	0.0102	1.40 (1.08-1.81)	0.70	0.71	0.4250	0.93 (0.79-1.10)
22	rs2284038	37635055	*RAC2*	A/G	0.65	0.63	0.6228	1.06 (0.84-1.35)	0.63	0.63	0.8236	0.98 (0.84-1.15)
23	rs5912838	78497118	*GPR174/ITM2A*	A/C	0.68	0.58	0.0025	1.50 (1.10-2.07)	0.70	0.58	1.31×10^{-5}	1.68 (1.36-2.07)

© 2019 Zhao S-X et al. *JAMA Network Open.*

Chr., chromosome; SNP, single nucleotide polymorphism; BP, base position; F, minor allele frequency; TPP, thyrotoxic hypokalemic periodic paralysis; Cons, controls; GD, Graves' disease; OR, odds ratio; CI, confidence interval. SNPs with fold met the the Bonferroni-corrected significance level in the combined populations.

© 2019 Zhao S-X et al. *JAMA Network Open.*

Continued eTable 3

SNP	TPPvsControl Combined (533 vs 3,249)						TPP VS GD (533 TPP vs 5,160 GD)			
	F_TPP	F_Cons	*P*-value	OR (95% CI)	Breslow-Day Test P-value	Power (%)	F_TPP	F_GD	*P*-value	OR (95% CI)
rs2843403	0.56	0.52	0.0138	1.20 (1.06-1.37)	0.0058	0.3	0.57	0.54	0.2241	1.09 (0.95-1.26)
rs11590198	0.59	0.54	0.0090	1.22 (1.07-1.39)	0.0053	0.6	0.59	0.58	0.4357	1.06 (0.92-1.22)
rs4648661	0.49	0.43	0.0007	1.26 (1.10-1.45)	0.0286	3.2	0.49	0.46	0.7034	1.03 (0.87-1.22)
rs3761959	0.47	0.40	0.0002	1.28 (1.12-1.45)	0.8443	4.1	0.47	0.45	0.3619	1.06 (0.93-1.20)
rs1265883	0.13	0.10	0.0031	1.33 (1.10-1.60)	0.5602	0.6	0.13	0.12	0.7397	1.03 (0.86-1.25)
rs1881145	0.68	0.60	3.25×10^{-6}	1.39 (1.21-1.60)	0.5796	23.1	0.67	0.63	0.0150	1.24 (1.08-1.43)
rs1534422	0.26	0.24	0.2412	1.10 (0.94-1.28)	0.5682	0.0	0.26	0.23	0.1063	1.14 (0.97-1.35)
rs4669857	0.61	0.58	0.0501	1.14 (1.00-1.30)	0.7215	0.0	0.61	0.61	0.7297	0.98 (0.86-1.12)
rs1024161	0.74	0.68	0.0002	1.31 (1.14-1.51)	0.0506	3.5	0.74	0.74	0.7871	0.98 (0.85-1.13)
rs13093110	0.37	0.40	0.0519	0.88 (0.77-1.00)	0.0975	0.0	0.37	0.38	0.4590	0.95 (0.83-1.09)
rs6806091	0.20	0.18	0.1187	1.14 (0.97-1.34)	0.6233	0.0	0.20	0.19	0.3341	1.10 (0.91-1.33)
rs6832151	0.42	0.35	3.07×10^{-5}	1.31 (1.15-1.49)	0.1327	7.2	0.42	0.40	0.2811	1.07 (0.94-1.22)
rs1368408	0.20	0.19	0.5022	1.06 (0.90-1.25)	0.7973	0.0	0.20	0.23	0.0482	0.85 (0.73-1.00)
**rs4947296**	0.26	0.14	3.08×10^{-22}	2.06 (1.77-2.39)	0.9366	100.0	0.26	0.21	0.0011	1.28 (1.11-1.48)
**rs1521**	0.91	0.81	3.63×10^{-18}	2.51 (2.02-3.13)	0.0222	100.0	0.91	0.88	0.0012	1.45 (1.16-1.81)
rs6903608	0.42	0.38	0.0102	1.18 (1.04-1.35)	0.1126	0.2	0.42	0.45	0.0644	0.89 (0.78-1.01)
**rs6457617**	0.60	0.47	2.37×10^{-17}	1.74 (1.53-1.97)	0.5321	99.6	0.60	0.47	5.35×10^{-5}	0.76 (0.67-0.87)
**rs2281388**	0.46	0.33	2.46×10^{-18}	1.74 (1.54-1.98)	0.6573	99.7	0.46	0.44	0.1111	1.09 (0.96-1.24)
rs2474619	0.68	0.57	0.0049	1.58 (1.38-1.81)	0.7114	4.4	0.68	0.66	0.2623	1.08 (0.94-1.23)
rs9355610	0.52	0.47	0.0004	1.26 (1.11-1.43)	0.2026	2.5	0.52	0.49	0.4178	0.95 (0.84-1.08)
rs2294025	0.22	0.19	0.0174	1.20 (1.03-1.39)	0.4660	0.0	0.22	0.22	0.7658	1.02 (0.88-1.19)
rs4736437	0.23	0.20	0.0601	1.15 (0.99-1.34)	0.5500	0.0	0.23	0.23	0.8697	0.99 (0.85-1.15)
rs505922	0.58	0.53	0.0054	1.19 (1.05-1.35)	0.3819	0.2	0.58	0.56	0.4909	1.05 (0.92-1.19)
rs12575636	0.11	0.09	0.0045	1.35 (1.10-1.66)	0.8147	0.2	0.11	0.11	0.9313	0.99 (0.77-1.27)
rs4409785	0.11	0.09	0.0115	1.32 (1.06-1.64)	0.8698	0.1	0.11	0.11	0.7157	1.04 (0.83-1.31)
rs4768412	0.26	0.23	0.0293	1.19 (1.02-1.38)	0.1100	0.0	0.26	0.23	0.0509	1.18 (1.00-1.38)
rs12101261	0.69	0.64	0.0013	1.25 (1.09-1.43)	0.2413	1.1	0.69	0.71	0.2199	0.92 (0.80-1.05)
rs1456988	0.55	0.53	0.1385	1.10 (0.97-1.24)	0.1091	0.0	0.55	0.56	0.6358	0.97 (0.86-1.10)
rs57348955	0.23	0.21	0.1378	1.13 (0.96-1.33)	0.5237	0.0	0.23	0.21	0.3113	1.11 (0.91-1.35)
rs4423435	0.55	0.53	0.3416	1.08 (0.95-1.23)	0.7097	0.0	0.55	0.54	0.5288	1.04 (0.91-1.19)
rs1883832	0.67	0.64	0.0209	1.17 (1.02-1.34)	0.8578	0.1	0.67	0.68	0.8347	0.99 (0.86-1.13)
rs229527	0.72	0.71	0.4323	1.05 (0.92-1.21)	0.0102	0.0	0.72	0.76	0.0164	0.84 (0.73-0.97)
rs2284038	0.64	0.63	0.9324	1.01 (0.88-1.15)	0.5935	0.0	0.64	0.66	0.0896	0.89 (0.78-1.02)

© 2019 Zhao S-X et al. *JAMA Network Open.*

rs5912838	0.69	0.58	5.91×10^{-8}	1.62 (1.36-1.93)	0.5791	92.4	0.69	0.64	0.4439	1.25 (1.05-1.49)

© 2019 Zhao S-X et al. *JAMA Network Open.*

eTable 4 Clinival and laboratory features of 537 TPP Patients		
**Study**	**Reference range**	**Result**
**Clinical feature**		
Gender (male/female)	NA	458 : 79
Age (years)	NA	34.8 ± 11.1
Age of onset (years)	NA	32.2 ± 11.0
BMI (kg/m2)	18.5-24.9	23.3 ± 3.5
**Plasma**		
TSH (mIU/ml)	0.35-4.94	0.3 ± 0.7
free Tri-iodothyronine (pmol/L)	2.63-5.70	15.8 ± 12.1
Free thyroxine (pmol/L)	9.01-19.04	45.9 ± 44.4
Initial K (mmol/l)	3.5-5.1	2.1 ± 0.5
TRAb (IU/L)	0-1.75	17.4 ± 26.3
TPOAb (U/mL)	0−60	462.9 ± 471.4
**No. of Seizures**	**No. of patients**	**%**
<3	322	56
3-5	199	34
>5	56	10
**Causes of Seizures**		
Yes	302	52
No	275	48
**Pattern**		
Paraplegia	339	59
Quadriplegia	230	40
Paraplegia/Quadriplegia	8	1

© 2019 Zhao S-X et al. *JAMA Network Open.*

TPP, thyrotoxic hypokalemic periodic paralysis; BMI, body mass index; TSH, thyroid stimulating hormone; K, potassium； TRAb, thyroid stimulating hormone receptor antibody; TPOAb, thyroid peroxidase antibody; TGAb, thyroglobulin antibody.

© 2019 Zhao S-X et al. *JAMA Network Open.*

eTable 5. TPP association results for the imputed and typed SNPs in the 4q31.3 region in the initial genome-wide scan						
SNP	BP	Alleles	MAF_TPP	MAF_Controls	*P*	OR (95%CI)
rs2404916	154995463	A/G	0.43	0.48	0.1067	0.83 (0.67-1.04)
rs1316990	154995958	T/C	0.38	0.34	0.1704	1.17 (0.93-1.47)
rs9307919	155000585	C/T	0.25	0.29	0.0784	0.80 (0.62-1.03)
rs56668364	155003037	T/C	0.01	0.02	0.4930	0.70 (0.26-1.94)
rs76048878	155004764	C/T	0.01	0.02	0.4930	0.70 (0.26-1.94)
rs79349629	155006701	T/C	0.01	0.02	0.4930	0.70 (0.26-1.94)
rs17372515	155007632	A/G	0.07	0.07	0.6763	1.09 (0.72-1.67)
rs79090748	155009728	A/C	0.01	0.02	0.4365	0.67 (0.24-1.85)
rs140086160	155010519	C/T	0.01	0.02	0.4365	0.67 (0.24-1.85)
rs17031138	155013194	T/C	0.36	0.34	0.4016	1.10 (0.88-1.38)
rs4328897	155014356	A/C	0.22	0.24	0.2780	0.86 (0.66-1.13)
rs6830300	155015920	A/G	0.45	0.42	0.3494	1.11 (0.89-1.39)
rs6837242	155017153	G/A	0.23	0.25	0.3105	0.87 (0.67-1.14)
rs4266272	155018810	C/T	0.46	0.43	0.2870	1.13 (0.90-1.40)
rs72737552	155019234	C/T	0.30	0.30	0.9582	0.99 (0.78-1.26)
rs4491994	155019886	T/C	0.45	0.43	0.3245	1.12 (0.90-1.39)
rs13114220	155020516	A/G	0.22	0.25	0.3059	0.87 (0.67-1.13)
rs12651074	155021115	C/T	0.46	0.43	0.2458	1.14 (0.91-1.42)
rs79971659	155021201	G/A	0.01	0.02	0.4371	0.67 (0.24-1.85)
rs74411393	155022684	A/G	0.01	0.02	0.4371	0.67 (0.24-1.85)
rs4389552	155023943	G/A	0.46	0.43	0.2877	1.13 (0.90-1.40)
rs145213675	155024432	A/G	0.30	0.30	0.9932	1.00 (0.79-1.27)
rs75098752	155025088	C/T	0.23	0.25	0.3033	0.87 (0.67-1.13)
rs6856560	155025315	A/G	0.47	0.44	0.3831	1.10 (0.88-1.37)
rs138489403	155025396	G/T	0.45	0.43	0.3065	1.12 (0.90-1.40)
rs57349224	155026052	A/G	0.30	0.30	0.9905	1.00 (0.79-1.27)
rs78639855	155026661	A/G	0.01	0.02	0.4376	0.67 (0.24-1.85)
rs143958276	155026663	A/G	0.01	0.02	0.4376	0.67 (0.24-1.85)
rs72737554	155027462	G/A	0.30	0.30	0.9876	1.00 (0.79-1.27)
rs142735742	155027546	C/T	0.01	0.02	0.4376	0.67 (0.24-1.85)
rs1878448	155027927	A/G	0.47	0.44	0.3562	1.11 (0.89-1.38)
rs184103999	155028968	A/G	0.01	0.02	0.4376	0.67 (0.24-1.85)
rs79276288	155028969	G/A	0.01	0.02	0.4183	0.66 (0.24-1.82)
rs75462484	155029113	A/G	0.09	0.08	0.6025	1.11 (0.75-1.64)
rs61296553	155029782	G/A	0.45	0.43	0.2999	1.12 (0.90-1.40)
rs147376737	155030274	G/T	0.01	0.02	0.4376	0.67 (0.24-1.85)
rs146575547	155031340	A/G	0.30	0.30	0.9947	1.00 (0.79-1.27)
rs191149851	155034593	G/A	0.01	0.02	0.4376	0.67 (0.24-1.85)
rs116684133	155035244	G/A	0.46	0.43	0.2543	1.14 (0.91-1.42)
rs148736458	155035277	G/T	0.45	0.43	0.3003	1.12 (0.90-1.40)
rs111476154	155036071	T/C	0.36	0.34	0.5378	1.07 (0.85-1.35)
rs72737556	155036550	C/T	0.30	0.30	0.9818	1.00 (0.79-1.27)
rs17031143	155036794	G/A	0.45	0.43	0.3183	1.12 (0.90-1.39)
rs78276196	155037495	A/C	0.09	0.08	0.5813	1.12 (0.75-1.65)
rs7686617	155039142	C/T	0.30	0.30	0.9186	1.01 (0.80-1.29)
rs72721306	155039628	C/T	0.30	0.30	0.9933	1.00 (0.79-1.27)
rs76832349	155039845	T/C	0.09	0.08	0.5831	1.12 (0.75-1.65)
rs79850297	155040141	T/C	0.01	0.02	0.5819	0.78 (0.31-1.93)
rs76600819	155040151	A/G	0.01	0.02	0.5819	0.78 (0.31-1.93)

© 2019 Zhao S-X et al. *JAMA Network Open.*

rs72721307	155040332	T/C	0.30	0.30	0.9176	1.01 (0.80-1.29)
rs12643111	155040416	T/G	0.30	0.30	0.9133	1.01 (0.80-1.29)
rs4484280	155047033	T/C	0.44	0.46	0.4039	0.91 (0.73-1.14)
rs2014131	155047531	T/C	0.03	0.03	0.4541	0.77 (0.39-1.53)
rs938716	155049484	T/C	0.29	0.29	0.9814	1.00 (0.79-1.28)
rs10028852	155062069	A/G	0.13	0.13	0.9692	0.99 (0.72-1.38)
rs72721320	155062328	T/C	0.13	0.13	0.9874	1.00 (0.72-1.39)
rs10029174	155062442	A/C	0.13	0.13	0.9899	1.00 (0.72-1.39)
rs10776528	155062911	T/G	0.13	0.13	0.9294	1.02 (0.73-1.41)
rs58975189	155063442	T/G	0.13	0.13	0.9170	1.02 (0.73-1.41)
rs75111051	155066262	G/T	0.13	0.13	0.8309	1.04 (0.75-1.43)
rs4349583	155071789	C/A	0.14	0.13	0.8478	1.03 (0.75-1.42)
rs4432731	155080330	T/C	0.10	0.09	0.6634	1.09 (0.75-1.57)
rs76751686	155081233	C/T	0.09	0.11	0.2857	0.81 (0.55-1.19)
rs17031190	155083771	A/G	0.09	0.11	0.2413	0.79 (0.54-1.17)
rs17031193	155084367	C/T	0.09	0.11	0.2168	0.78 (0.53-1.16)
rs2897003	155084602	C/T	0.09	0.11	0.2216	0.79 (0.53-1.16)
rs17031199	155085018	T/G	0.09	0.11	0.1635	0.76 (0.52-1.12)
rs36123238	155085107	A/G	0.10	0.09	0.7242	1.07 (0.74-1.56)
rs17031202	155086743	G/A	0.09	0.11	0.2254	0.79 (0.54-1.16)
rs11728514	155114394	C/T	0.03	0.03	0.7245	1.11 (0.61-2.03)
rs6838705	155122349	G/A	0.10	0.13	0.1371	0.76 (0.53-1.09)
rs4393979	155123244	G/A	0.10	0.13	0.1058	0.74 (0.51-1.07)
rs113561709	155124385	C/T	0.14	0.13	0.7677	1.05 (0.76-1.45)
rs11099942	155132707	T/C	0.03	0.05	0.3595	0.76 (0.42-1.38)
rs4385038	155135688	G/A	0.24	0.27	0.3315	0.88 (0.68-1.14)
rs4425362	155138400	C/T	0.26	0.26	0.9570	0.99 (0.77-1.27)
rs79225578	155139560	G/A	0.06	0.08	0.1854	0.73 (0.46-1.16)
rs72968805	155143449	C/T	0.14	0.13	0.7773	1.05 (0.76-1.44)
rs75617643	155145504	T/C	0.07	0.09	0.1413	0.72 (0.47-1.12)
rs4696538	155146500	A/G	0.06	0.08	0.1793	0.73 (0.46-1.16)
rs151312587	155149413	T/G	0.06	0.08	0.1782	0.73 (0.46-1.16)
rs17031259	155150556	A/C	0.06	0.08	0.1789	0.73 (0.46-1.16)
rs9994961	155150764	A/C	0.06	0.08	0.1750	0.72 (0.45-1.15)
rs10029359	155150910	T/C	0.06	0.08	0.1750	0.72 (0.45-1.15)
rs74915192	155151440	T/C	0.14	0.13	0.6205	1.08 (0.79-1.49)
rs4283645	155152861	T/G	0.06	0.08	0.2055	0.74 (0.47-1.17)
rs10517589	155153299	T/G	0.06	0.08	0.1666	0.72 (0.45-1.15)
rs56020351	155153789	C/A	0.14	0.13	0.6884	1.07 (0.78-1.47)
rs6852506	155154654	G/A	0.14	0.13	0.6709	1.07 (0.78-1.47)
rs17031277	155155318	A/G	0.14	0.13	0.7470	1.05 (0.77-1.45)
rs7655799	155156207	G/A	0.21	0.23	0.5241	0.92 (0.70-1.20)
rs61746132	155156412	A/G	0.14	0.13	0.7539	1.05 (0.77-1.45)
rs61741046	155156542	A/G	0.03	0.04	0.2711	0.71 (0.38-1.32)
rs61743677	155157015	C/T	0.07	0.09	0.1822	0.75 (0.49-1.15)
rs6832477	155159162	C/T	0.07	0.09	0.2416	0.78 (0.51-1.19)
rs6535989	155161666	C/T	0.21	0.23	0.5422	0.92 (0.70-1.20)
rs3811737	155162154	C/T	0.07	0.09	0.2457	0.78 (0.51-1.19)
rs3811736	155162338	A/G	0.14	0.13	0.7383	1.06 (0.77-1.45)
rs11734901	155181767	C/A	0.51	0.47	0.1789	1.17 (0.94-1.45)
rs3828478	155182784	G/A	0.51	0.47	0.1602	1.17 (0.94-1.46)
rs11099944	155184454	A/G	0.51	0.47	0.2111	1.15 (0.92-1.44)
rs78718534	155184946	G/A	0.03	0.04	0.3533	0.72 (0.37-1.43)
rs11733437	155185893	C/A	0.51	0.47	0.2380	1.14 (0.92-1.43)
rs12643125	155186191	T/C	0.51	0.47	0.2380	1.14 (0.92-1.43)

© 2019 Zhao S-X et al. *JAMA Network Open.*

rs12643173	155186399	T/C	0.51	0.47	0.2380	1.14 (0.92-1.43)
rs6832806	155186976	T/C	0.46	0.49	0.3519	0.90 (0.72-1.12)
rs11731663	155187341	C/T	0.51	0.47	0.2337	1.15 (0.92-1.43)
rs12503824	155187756	T/C	0.03	0.04	0.3541	0.72 (0.37-1.43)
rs10010151	155188381	A/G	0.46	0.49	0.3564	0.90 (0.72-1.12)
rs12509842	155189006	G/T	0.03	0.04	0.3541	0.72 (0.37-1.43)
rs28668156	155189123	T/C	0.46	0.49	0.3654	0.90 (0.72-1.13)
rs28554124	155189297	G/A	0.46	0.49	0.3564	0.90 (0.72-1.12)
rs7666541	155190818	G/A	0.51	0.47	0.2314	1.15 (0.92-1.43)
rs4386584	155191451	C/T	0.51	0.47	0.2314	1.15 (0.92-1.43)
rs11726850	155192017	T/C	0.51	0.47	0.2314	1.15 (0.92-1.43)
rs11726919	155192201	T/C	0.51	0.47	0.2314	1.15 (0.92-1.43)
rs4073438	155192424	C/A	0.03	0.04	0.3541	0.72 (0.37-1.43)
rs4461508	155192697	A/G	0.03	0.04	0.4638	0.78 (0.41-1.50)
rs4403033	155193315	A/C	0.51	0.47	0.2315	1.15 (0.92-1.43)
rs4312742	155193398	A/G	0.51	0.47	0.2273	1.15 (0.92-1.43)
rs7440122	155193767	G/A	0.51	0.47	0.1969	1.16 (0.93-1.45)
rs4327464	155193969	G/A	0.51	0.47	0.2315	1.15 (0.92-1.43)
rs35315598	155194424	G/A	0.51	0.47	0.2372	1.14 (0.92-1.43)
rs4575978	155194629	G/A	0.51	0.47	0.2372	1.14 (0.92-1.43)
rs12506449	155194975	T/C	0.03	0.04	0.5176	0.81 (0.42-1.54)
rs4235240	155195014	T/C	0.03	0.04	0.4638	0.78 (0.41-1.50)
rs4235241	155195184	T/G	0.51	0.47	0.2209	1.15 (0.92-1.43)
rs80000145	155195762	T/C	0.02	0.03	0.3971	0.73 (0.35-1.50)
rs6823540	155197891	T/G	0.23	0.23	0.7301	0.96 (0.74-1.24)
rs72617460	155207953	T/G	0.11	0.11	0.7858	0.95 (0.67-1.35)
rs12508314	155209159	G/A	0.11	0.11	0.7795	0.95 (0.67-1.35)
rs10001032	155209964	T/G	0.52	0.49	0.2310	1.15 (0.92-1.43)
rs11099948	155210418	C/T	0.52	0.49	0.2310	1.15 (0.92-1.43)
rs67916370	155212484	T/G	0.11	0.12	0.7471	0.94 (0.67-1.34)
rs67121281	155215027	G/A	0.11	0.12	0.7471	0.94 (0.67-1.34)
rs72617461	155223374	G/A	0.11	0.12	0.7344	0.94 (0.66-1.34)
rs12504514	155223519	A/G	0.11	0.12	0.7344	0.94 (0.66-1.34)
rs67518652	155224423	T/C	0.11	0.12	0.7084	0.94 (0.66-1.33)
rs4463049	155227275	A/C	0.11	0.12	0.6678	0.93 (0.65-1.32)
rs4696546	155229324	T/C	0.52	0.49	0.2814	1.13 (0.91-1.41)
rs4696547	155229503	C/T	0.52	0.49	0.2814	1.13 (0.91-1.41)
rs4696205	155232591	T/G	0.52	0.49	0.2994	1.12 (0.90-1.40)
rs6847387	155233170	G/T	0.38	0.39	0.4720	0.92 (0.73-1.15)
rs72617462	155233521	T/C	0.11	0.12	0.6543	0.92 (0.65-1.31)
rs12644248	155235392	G/A	0.11	0.12	0.6543	0.92 (0.65-1.31)
rs66571236	155236548	G/A	0.11	0.12	0.6543	0.92 (0.65-1.31)
rs12640050	155237904	T/C	0.11	0.12	0.6543	0.92 (0.65-1.31)
rs13111065	155238342	A/G	0.52	0.49	0.2815	1.13 (0.91-1.41)
rs56274873	155240057	G/A	0.30	0.35	0.0544	0.79 (0.62-1.01)
rs10049695	155240256	C/T	0.22	0.14	3.01E-05	1.75 (1.34-2.29)
rs13132851	155241173	G/A	0.28	0.28	0.9790	1.00 (0.79-1.28)
rs11935503	155241438	A/G	0.29	0.35	0.0517	0.79 (0.62-1.00)
rs11935573	155241572	A/G	0.29	0.35	0.0513	0.79 (0.62-1.00)
rs62331873	155242383	T/C	0.29	0.35	0.0517	0.79 (0.62-1.00)
rs62331874	155242505	T/G	0.29	0.35	0.0517	0.79 (0.62-1.00)
rs13109747	155243573	C/T	0.22	0.14	4.24×10^{-5}	1.73 (1.33-2.26)
rs1352714	155243604	T/C	0.22	0.14	3.69×10^{-5}	1.74 (1.33-2.27)
rs12500437	155244475	G/T	0.22	0.14	4.24×10^{-5}	1.73 (1.33-2.26)
rs1490673	155244671	T/G	0.29	0.35	0.0506	0.79 (0.62-1.00)

© 2019 Zhao S-X et al. *JAMA Network Open.*

rs6535994	155245198	C/T	0.22	0.14	4.24×10^{-5}	1.73 (1.33-2.26)
rs56214025	155245204	A/G	0.29	0.35	0.0506	0.79 (0.62-1.00)
rs1388084	155245811	C/T	0.22	0.14	4.24×10^{-5}	1.73 (1.33-2.26)
rs13131753	155246568	C/T	0.52	0.49	0.3038	1.12 (0.90-1.40)
rs9637650	155246673	C/T	0.22	0.14	3.76×10^{-5}	1.74 (1.33-2.27)
rs2896945	155247165	T/C	0.29	0.35	0.0476	0.79 (0.62-1.00)
rs2054962	155247235	G/A	0.52	0.49	0.3035	1.12 (0.90-1.40)
rs2130712	155247554	C/A	0.22	0.14	3.76×10^{-5}	1.74 (1.33-2.27)
rs7681523	155247884	C/T	0.52	0.49	0.3072	1.12 (0.90-1.40)
rs1388083	155248643	C/T	0.31	0.35	0.0858	0.81 (0.64-1.03)
rs1388082	155248664	A/G	0.21	0.14	0.0001	1.69 (1.29-2.22)
rs2054961	155248845	G/T	0.29	0.29	0.9691	1.01 (0.79-1.28)
rs2130711	155248922	T/G	0.21	0.14	0.0001	1.70 (1.29-2.23)
rs2404704	155249118	C/T	0.22	0.14	2.69×10^{-5}	1.76 (1.34-2.30)
rs12651681	155249362	G/A	0.11	0.11	0.7845	0.95 (0.67-1.35)
rs10517595	155251015	T/G	0.19	0.22	0.1915	0.83 (0.63-1.10)
rs2130710	155251785	C/T	0.31	0.35	0.0995	0.82 (0.65-1.04)
rs77300771	155253016	C/T	0.20	0.12	0.0001	1.73 (1.30-2.29)
rs118016158	155253393	A/G	0.19	0.12	0.0002	1.70 (1.28-2.26)
rs6858157	155256177	G/A	0.34	0.37	0.2334	0.87 (0.69-1.10)
rs1021381	155257709	C/T	0.34	0.38	0.2076	0.86 (0.68-1.09)
rs62331879	155258393	T/C	0.32	0.37	0.0885	0.82 (0.65-1.03)
rs6815049	155262171	G/A	0.34	0.38	0.1433	0.84 (0.67-1.06)
rs184685103	155262395	T/C	0.02	0.02	0.5605	1.24 (0.60-2.59)
rs189990891	155262450	G/A	0.02	0.02	0.5605	1.24 (0.60-2.59)
rs13112205	155266359	T/C	0.26	0.26	0.7871	0.97 (0.75-1.24)
rs13112673	155266439	A/G	0.26	0.26	0.7871	0.97 (0.75-1.24)
rs13113312	155266731	T/G	0.26	0.26	0.7909	0.97 (0.75-1.24)
rs11726534	155269311	G/A	0.33	0.37	0.1279	0.84 (0.66-1.05)
rs4696207	155269388	A/C	0.26	0.27	0.7287	0.96 (0.75-1.23)
rs7681342	155270460	T/G	0.26	0.27	0.9109	0.99 (0.77-1.26)
rs17031489	155273743	G/A	0.63	0.56	0.0058	1.37 (1.09-1.72)
rs10028929	155275122	C/A	0.13	0.12	0.4939	1.12 (0.81-1.55)
rs6844979	155275324	T/C	0.13	0.12	0.4876	1.12 (0.81-1.56)
rs1490675	155278862	T/C	0.13	0.12	0.5377	1.11 (0.80-1.54)
rs35132553	155279102	A/C	0.37	0.45	0.0053	0.73 (0.58-0.91)
rs13117053	155279294	G/A	0.13	0.12	0.5377	1.11 (0.80-1.54)
rs6536001	155281288	A/G	0.13	0.12	0.4678	1.13 (0.82-1.56)
rs7687041	155281772	G/A	0.13	0.12	0.4820	1.12 (0.81-1.55)
rs10017772	155287535	C/T	0.49	0.42	0.0117	1.32 (1.06-1.65)
rs6419274	155291868	T/C	0.10	0.09	0.5078	1.14 (0.78-1.65)
rs113280523	155292872	T/G	0.09	0.05	0.0005	1.98 (1.34-2.94)
rs4696553	155293079	G/A	0.10	0.09	0.5525	1.12 (0.77-1.62)
rs4696554	155293153	C/T	0.10	0.09	0.5004	1.13 (0.79-1.63)
rs11721758	155295076	G/A	0.39	0.36	0.3295	1.12 (0.89-1.40)
rs13111929	155296207	A/G	0.25	0.24	0.4682	1.10 (0.85-1.41)
rs6536003	155297245	T/G	0.10	0.09	0.6611	1.09 (0.75-1.58)
rs6815493	155297291	A/G	0.09	0.05	0.0016	1.86 (1.26-2.76)
rs6536004	155297559	G/A	0.27	0.28	0.5364	0.93 (0.72-1.19)
rs7688746	155297826	T/C	0.27	0.28	0.5314	0.92 (0.72-1.19)
rs72723371	155298050	T/C	0.17	0.19	0.2960	0.86 (0.64-1.15)
rs13101522	155298116	T/C	0.27	0.28	0.5321	0.92 (0.72-1.19)
rs66478552	155298773	G/T	0.17	0.19	0.3703	0.88 (0.66-1.17)
rs34503070	155299027	C/T	0.36	0.33	0.3320	1.12 (0.89-1.40)
rs4696555	155299203	T/C	0.17	0.19	0.3703	0.88 (0.66-1.17)

© 2019 Zhao S-X et al. *JAMA Network Open.*

rs6811038	155299346	T/C	0.27	0.28	0.5791	0.93 (0.73-1.20)
rs35687519	155299382	T/G	0.17	0.19	0.3621	0.87 (0.66-1.17)
rs13134088	155299483	G/A	0.17	0.19	0.3621	0.87 (0.66-1.17)
rs4696556	155300448	T/C	0.10	0.09	0.7077	1.08 (0.74-1.56)
rs17373342	155300546	A/G	0.17	0.19	0.3520	0.87 (0.65-1.16)
rs1388068	155300694	G/A	0.09	0.05	0.0017	1.86 (1.25-2.75)
rs1388069	155300870	G/A	0.17	0.19	0.3487	0.87 (0.65-1.16)
rs1388070	155300938	A/G	0.36	0.33	0.3451	1.11 (0.89-1.40)
rs4696557	155301247	G/A	0.27	0.28	0.5984	0.94 (0.73-1.20)
rs8180116	155301356	A/G	0.17	0.19	0.3487	0.87 (0.65-1.16)
rs1388071	155301686	T/C	0.10	0.09	0.6770	1.08 (0.75-1.57)
rs13125423	155301894	T/C	0.27	0.28	0.5694	0.93 (0.73-1.19)
rs8180151	155303100	C/T	0.10	0.09	0.6681	1.09 (0.75-1.58)
rs11733376	155303386	A/G	0.17	0.19	0.3811	0.88 (0.66-1.17)
rs4696558	155303802	T/C	0.17	0.19	0.3811	0.88 (0.66-1.17)
rs7674383	155304371	A/G	0.27	0.28	0.6260	0.94 (0.74-1.21)
rs35640569	155304448	T/G	0.17	0.19	0.3811	0.88 (0.66-1.17)
rs17301342	155305233	T/C	0.36	0.33	0.2899	1.13 (0.90-1.42)
rs34927139	155305491	C/T	0.36	0.33	0.2941	1.13 (0.90-1.42)
rs116156492	155306766	G/A	0.09	0.05	0.0013	1.89 (1.27-2.80)
rs12651672	155307037	T/C	0.24	0.27	0.2680	0.87 (0.67-1.12)
rs10857274	155307426	C/T	0.27	0.28	0.7746	0.97 (0.75-1.24)
rs17031541	155307651	C/T	0.09	0.05	0.0013	1.89 (1.27-2.80)
rs76410096	155309323	A/G	0.09	0.05	0.0026	1.83 (1.23-2.72)
rs60164156	155311757	A/G	0.09	0.05	0.0025	1.84 (1.23-2.74)
rs11099952	155311858	C/T	0.03	0.01	0.0328	2.07 (1.05-4.08)
rs57468943	155312212	A/G	0.09	0.05	0.0025	1.84 (1.23-2.74)
rs9992566	155312995	T/C	0.03	0.01	0.0363	2.04 (1.04-4.02)
rs17031566	155318412	G/A	0.24	0.27	0.2280	0.86 (0.66-1.11)
rs1388089	155318476	T/G	0.24	0.27	0.2250	0.85 (0.66-1.11)
rs7686473	155319043	A/G	0.24	0.27	0.2340	0.86 (0.66-1.11)
rs7693455	155320054	G/A	0.24	0.27	0.2297	0.86 (0.66-1.11)
rs76025992	155320428	C/T	0.09	0.05	0.0029	1.82 (1.22-2.71)
rs12647084	155321311	C/A	0.33	0.32	0.7444	1.04 (0.82-1.31)
rs6828604	155322112	C/T	0.23	0.27	0.2035	0.85 (0.65-1.10)
rs6811973	155322636	T/C	0.32	0.32	0.8705	1.02 (0.80-1.29)
rs147822301	155322882	T/C	0.09	0.05	0.0029	1.82 (1.22-2.71)
rs7349602	155323014	T/C	0.23	0.26	0.2051	0.85 (0.65-1.10)
rs13123721	155323951	T/C	0.24	0.27	0.2186	0.85 (0.66-1.10)
rs4696561	155324695	C/T	0.24	0.27	0.2203	0.85 (0.66-1.10)
rs6849220	155325049	A/C	0.09	0.05	0.0016	1.86 (1.26-2.76)
rs7697696	155326092	T/C	0.03	0.01	0.0322	2.07 (1.05-4.09)
rs11099953	155329567	C/T	0.23	0.26	0.2416	0.86 (0.66-1.11)
rs1490674	155330282	T/C	0.35	0.33	0.3256	1.12 (0.89-1.41)
rs59008095	155330652	A/G	0.09	0.05	0.0015	1.87 (1.26-2.78)
rs73854741	155331350	G/A	0.24	0.26	0.2648	0.87 (0.67-1.12)
rs28590354	155331351	A/G	0.03	0.01	0.0405	2.01 (1.02-3.95)
rs72968061	155331651	G/A	0.24	0.26	0.2592	0.86 (0.67-1.12)
rs28795483	155331675	T/C	0.33	0.31	0.6373	1.06 (0.84-1.33)
rs4550900	155331774	T/C	0.33	0.32	0.6558	1.05 (0.83-1.33)
rs4615159	155332046	T/C	0.24	0.26	0.2833	0.87 (0.67-1.13)
rs7681004	155332330	G/T	0.09	0.05	0.0014	1.88 (1.27-2.79)
rs6851087	155333714	T/C	0.03	0.02	0.0498	1.95 (0.99-3.83)
rs7674936	155334762	A/G	0.03	0.01	0.0361	2.04 (1.04-4.02)
rs6536008	155338890	G/A	0.08	0.09	0.5997	0.90 (0.60-1.34)

© 2019 Zhao S-X et al. *JAMA Network Open.*

rs902455	155339195	G/A	0.09	0.09	0.6639	0.92 (0.62-1.35)
rs7686357	155339902	T/G	0.08	0.09	0.5962	0.90 (0.60-1.34)
rs13123127	155340110	C/T	0.08	0.09	0.6248	0.91 (0.61-1.34)
rs2896868	155343176	T/C	0.08	0.09	0.5415	0.88 (0.59-1.32)
rs4235245	155343387	C/T	0.09	0.10	0.6093	0.90 (0.61-1.33)
rs10005270	155343913	T/C	0.08	0.09	0.5415	0.88 (0.59-1.32)
rs7659456	155344762	C/T	0.08	0.09	0.5689	0.89 (0.60-1.32)
rs4696564	155346362	T/C	0.08	0.09	0.6440	0.91 (0.61-1.36)
rs4696566	155347856	A/C	0.08	0.09	0.5613	0.89 (0.60-1.32)
rs4696569	155348012	A/G	0.08	0.09	0.5477	0.88 (0.59-1.32)
rs4696570	155348049	A/G	0.08	0.09	0.5596	0.89 (0.60-1.32)
rs7685089	155348490	A/G	0.08	0.09	0.5596	0.89 (0.60-1.32)
rs7685617	155348655	C/T	0.08	0.09	0.5596	0.89 (0.60-1.32)
rs6843389	155348683	A/C	0.08	0.09	0.5504	0.88 (0.59-1.32)
rs1907152	155349311	T/C	0.08	0.09	0.5596	0.89 (0.60-1.32)
rs1907151	155349445	A/C	0.08	0.09	0.5493	0.89 (0.60-1.31)
rs1907150	155349463	G/A	0.08	0.09	0.5596	0.89 (0.60-1.32)
rs6849849	155349560	A/G	0.08	0.09	0.5596	0.89 (0.60-1.32)
rs6828176	155350111	T/C	0.08	0.10	0.4911	0.87 (0.59-1.29)
rs9995943	155351598	G/A	0.20	0.19	0.5586	1.09 (0.82-1.43)
rs10030235	155351627	C/T	0.20	0.19	0.5942	1.08 (0.82-1.42)
rs35418965	155351937	G/A	0.20	0.19	0.5641	1.08 (0.82-1.43)
rs10000511	155351991	C/T	0.20	0.19	0.5641	1.08 (0.82-1.43)
rs7677032	155352444	G/A	0.08	0.09	0.5436	0.88 (0.59-1.32)
rs17031636	155353048	C/A	0.08	0.09	0.5429	0.88 (0.59-1.32)
rs1490656	155353882	C/T	0.20	0.19	0.5879	1.08 (0.82-1.42)
rs1388076	155354083	A/C	0.20	0.19	0.6214	1.07 (0.81-1.41)
rs4696572	155354916	T/C	0.28	0.28	0.9583	1.01 (0.79-1.28)
rs6833061	155356110	T/C	0.08	0.09	0.5173	0.88 (0.59-1.31)
rs6856249	155356127	A/G	0.20	0.19	0.5999	1.08 (0.82-1.42)
rs10013533	155356273	C/T	0.20	0.19	0.5999	1.08 (0.82-1.42)
rs12506034	155356610	C/T	0.20	0.19	0.5999	1.08 (0.82-1.42)
rs1388087	155357085	T/G	0.20	0.19	0.5988	1.08 (0.82-1.42)
rs1388088	155357218	G/A	0.20	0.19	0.6045	1.08 (0.82-1.41)
rs1388066	155357388	T/G	0.20	0.19	0.5892	1.08 (0.82-1.42)
rs1388067	155357475	T/C	0.20	0.19	0.5388	1.09 (0.83-1.44)
rs61283912	155359733	A/G	0.20	0.19	0.5696	1.08 (0.82-1.42)
rs9884952	155359761	G/A	0.20	0.19	0.5696	1.08 (0.82-1.42)
rs9884132	155359968	G/A	0.20	0.19	0.5696	1.08 (0.82-1.42)
rs9884570	155360001	A/C	0.20	0.19	0.5696	1.08 (0.82-1.42)
rs9884574	155360012	T/G	0.20	0.19	0.5696	1.08 (0.82-1.42)
rs11099954	155360144	C/T	0.20	0.19	0.5696	1.08 (0.82-1.42)
rs10014536	155360533	T/C	0.20	0.19	0.5696	1.08 (0.82-1.42)
rs10014635	155360593	A/C	0.20	0.19	0.5696	1.08 (0.82-1.42)
rs4399970	155360707	C/T	0.20	0.19	0.5696	1.08 (0.82-1.42)
rs1032336	155360919	T/C	0.20	0.19	0.5696	1.08 (0.82-1.42)
rs1032335	155361010	G/A	0.20	0.19	0.5696	1.08 (0.82-1.42)
rs1907155	155361244	G/T	0.21	0.19	0.5799	1.08 (0.82-1.42)
rs1907154	155361275	A/G	0.20	0.19	0.5696	1.08 (0.82-1.42)
rs1490684	155362154	A/C	0.20	0.19	0.5696	1.08 (0.82-1.42)
rs13146084	155364122	T/C	0.25	0.26	0.9754	1.00 (0.77-1.28)
rs4267717	155365967	T/C	0.24	0.24	0.9925	1.00 (0.77-1.29)
rs1490659	155366714	T/G	0.24	0.24	0.9837	1.00 (0.78-1.30)
rs35283999	155370024	T/C	0.24	0.24	0.9798	1.00 (0.77-1.29)
rs4696576	155371246	G/A	0.31	0.30	0.8203	1.03 (0.81-1.30)

© 2019 Zhao S-X et al. *JAMA Network Open.*

rs62330369	155379708	G/A	0.17	0.17	0.9229	0.99 (0.74-1.32)
rs11731813	155379883	A/G	0.32	0.30	0.4392	1.10 (0.87-1.39)
rs1490677	155380037	A/C	0.15	0.12	0.1848	1.23 (0.90-1.68)
rs28429061	155380185	C/T	0.17	0.17	0.9229	0.99 (0.74-1.32)
rs12643014	155381726	A/G	0.15	0.12	0.1837	1.23 (0.90-1.68)
rs13124347	155382037	T/C	0.15	0.13	0.2103	1.22 (0.89-1.66)
rs13148992	155382108	G/A	0.32	0.30	0.4546	1.09 (0.86-1.39)
rs11725265	155382697	T/C	0.15	0.13	0.2103	1.22 (0.89-1.66)
rs1907156	155384995	A/G	0.15	0.12	0.1815	1.24 (0.91-1.69)
rs12649437	155387171	G/A	0.17	0.17	0.8545	0.97 (0.73-1.30)
rs72725345	155387524	A/G	0.15	0.12	0.1848	1.23 (0.90-1.68)
rs56137436	155389000	C/A	0.15	0.12	0.1837	1.23 (0.90-1.68)
rs10857276	155391441	G/T	0.15	0.13	0.2091	1.22 (0.89-1.66)
rs11935196	155392190	G/A	0.15	0.13	0.2067	1.22 (0.90-1.66)
rs4696582	155396138	C/T	0.15	0.12	0.1948	1.23 (0.90-1.67)
rs4696583	155396176	T/C	0.15	0.13	0.2160	1.21 (0.89-1.65)
rs72725351	155397357	C/A	0.15	0.12	0.1948	1.23 (0.90-1.67)
rs4696584	155397517	A/G	0.15	0.12	0.2111	1.22 (0.89-1.66)
rs6819298	155397921	G/T	0.15	0.12	0.1804	1.24 (0.91-1.69)
rs6844895	155397991	T/C	0.15	0.12	0.1804	1.24 (0.91-1.69)
rs72725354	155399841	G/A	0.15	0.12	0.1751	1.24 (0.91-1.69)
rs4696585	155400323	A/G	0.15	0.13	0.2232	1.21 (0.89-1.65)
rs59686383	155400718	C/T	0.16	0.13	0.1673	1.24 (0.91-1.67)
rs61270934	155400949	C/T	0.16	0.13	0.1666	1.24 (0.91-1.67)
rs10517593	155400984	C/T	0.16	0.14	0.1967	1.22 (0.90-1.64)
rs1552638	155402365	A/G	0.16	0.13	0.1638	1.24 (0.92-1.67)
rs1552639	155402499	A/G	0.16	0.13	0.1638	1.24 (0.92-1.67)
rs56223712	155403065	A/G	0.16	0.13	0.1638	1.24 (0.92-1.67)
rs6536014	155403068	C/T	0.16	0.13	0.1638	1.24 (0.92-1.67)
rs1490666	155403285	G/T	0.16	0.13	0.1638	1.24 (0.92-1.67)
rs72729002	155405405	G/A	0.16	0.13	0.1508	1.25 (0.92-1.68)
rs1388081	155405579	G/A	0.16	0.13	0.1508	1.25 (0.92-1.68)
rs4696587	155405655	G/A	0.16	0.13	0.1548	1.24 (0.92-1.68)
rs4696588	155405717	C/T	0.16	0.13	0.1548	1.24 (0.92-1.68)
rs4150	155405919	C/T	0.16	0.13	0.1548	1.24 (0.92-1.68)
rs1388075	155406720	C/T	0.16	0.13	0.1423	1.25 (0.93-1.69)
rs1490652	155407089	A/G	0.16	0.13	0.1276	1.26 (0.93-1.71)
rs4235246	155419003	T/C	0.18	0.14	0.0770	1.30 (0.97-1.73)
rs10014500	155420487	A/G	0.17	0.14	0.0495	1.33 (1.00-1.79)
rs6819398	155420809	A/G	0.24	0.21	0.3189	1.14 (0.88-1.48)
rs4533754	155421442	C/A	0.06	0.07	0.6327	0.89 (0.56-1.43)
rs4345160	155421710	G/A	0.18	0.15	0.1455	1.24 (0.93-1.65)
rs6832166	155422398	A/G	0.24	0.21	0.3359	1.14 (0.88-1.48)
rs7681708	155423520	T/G	0.18	0.15	0.1290	1.25 (0.94-1.67)
rs4323084	155424231	C/T	0.24	0.21	0.2558	1.16 (0.90-1.50)
rs6846825	155424832	T/G	0.18	0.14	0.0654	1.31 (0.98-1.75)
rs4132149	155425156	T/G	0.06	0.07	0.7055	0.92 (0.58-1.45)
rs4696595	155425967	A/G	0.18	0.14	0.1000	1.27 (0.95-1.70)
rs10019863	155427505	T/G	0.18	0.14	0.1046	1.27 (0.95-1.69)
rs6536017	155430666	A/G	0.06	0.07	0.7495	0.93 (0.59-1.46)
rs4482740	155446305	A/G	0.27	0.26	0.6589	1.06 (0.83-1.35)

SNP, single nucleotide polymorphism; BP, base position; MAF, minor allele frequency; TPP, thyrotoxic hypokalemic periodic paralysis; OR, odds ratio; CI, confidence interval.

© 2019 Zhao S-X et al. *JAMA Network Open.*

eTable 6. TPP association results for the imputed and typed SNPs in the 11q14.1 region in the initial genome-wide scan						
SNP	BP	Alleles	MAF_TPP	MAF_Controls	*P*	OR (95%CI)
rs76259055	77155326	C/A	0.06	0.06	0.7407	0.92 (0.58-1.48)
rs2852394	77155851	A/C	0.19	0.19	0.7473	0.95 (0.72-1.27)
rs3015982	77157188	G/T	0.06	0.06	0.7407	0.92 (0.58-1.48)
rs7119033	77158800	C/A	0.41	0.44	0.2300	0.87 (0.70-1.09)
rs3019258	77159119	C/T	0.54	0.50	0.1782	1.17 (0.93-1.45)
rs3019236	77161420	T/C	0.06	0.06	0.7407	0.92 (0.58-1.48)
rs11237189	77161696	G/A	0.18	0.19	0.7503	0.95 (0.72-1.27)
rs7112264	77161741	G/T	0.22	0.25	0.2253	0.84 (0.65-1.10)
rs3019234	77162790	A/G	0.06	0.06	0.7188	0.92 (0.57-1.47)
rs11237190	77163263	C/T	0.21	0.24	0.2400	0.85 (0.65-1.11)
rs3019233	77163500	G/A	0.06	0.06	0.7407	0.92 (0.58-1.48)
rs57305199	77164354	A/G	0.21	0.24	0.2473	0.85 (0.65-1.11)
rs3015983	77164904	C/T	0.06	0.06	0.7582	0.93 (0.58-1.48)
rs10899378	77166652	G/A	0.19	0.19	0.7696	0.96 (0.72-1.27)
rs79930550	77166936	G/A	0.22	0.25	0.2606	0.86 (0.66-1.11)
rs17135670	77166977	C/T	0.22	0.25	0.2552	0.85 (0.66-1.11)
rs11237193	77167670	C/T	0.19	0.19	0.7696	0.96 (0.72-1.27)
rs3015985	77169102	G/T	0.06	0.06	0.7815	0.94 (0.59-1.49)
rs10899379	77169671	G/A	0.40	0.44	0.1826	0.86 (0.69-1.07)
rs11828786	77169828	C/A	0.22	0.25	0.2235	0.84 (0.65-1.10)
rs7934230	77171171	A/G	0.06	0.06	0.8038	0.94 (0.59-1.51)
rs3015988	77171649	C/T	0.06	0.06	0.8038	0.94 (0.59-1.51)
rs3015990	77172200	C/A	0.06	0.06	0.8038	0.94 (0.59-1.51)
rs947809	77172478	T/C	0.21	0.24	0.2400	0.85 (0.65-1.11)
rs11237195	77172833	C/T	0.18	0.19	0.8054	0.96 (0.73-1.28)
rs34025917	77173244	T/C	0.06	0.06	0.8797	0.96 (0.60-1.54)
rs3019249	77175444	C/T	0.27	0.31	0.1864	0.84 (0.66-1.08)
rs12271542	77175552	G/T	0.19	0.19	0.8481	0.97 (0.73-1.29)
rs11237196	77176731	A/G	0.19	0.19	0.8923	0.98 (0.74-1.30)
rs3019238	77204866	G/A	0.27	0.26	0.5863	1.07 (0.84-1.37)
rs3018480	77208493	T/C	0.27	0.26	0.5593	1.08 (0.84-1.38)
rs3019247	77214428	T/C	0.06	0.06	0.7885	0.94 (0.59-1.49)
rs3018476	77215077	T/C	0.06	0.06	0.7938	0.94 (0.60-1.49)
rs3019246	77215589	C/T	0.06	0.07	0.7545	0.93 (0.59-1.47)
rs3019245	77215675	A/G	0.06	0.07	0.8378	0.95 (0.61-1.49)
rs6592724	77224972	A/G	0.42	0.36	0.0307	1.28 (1.02-1.59)
rs7109180	77226842	G/A	0.34	0.39	0.0709	0.81 (0.64-1.02)
rs1945753	77235279	A/G	0.33	0.37	0.0976	0.82 (0.65-1.04)
rs6592725	77240152	T/C	0.44	0.48	0.1786	0.86 (0.69-1.07)
rs72943494	77253788	A/G	0.25	0.17	0.0001	1.66 (1.28-2.14)
rs11823651	77253879	A/G	0.07	0.10	0.1406	0.73 (0.48-1.10)
rs72943497	77254266	T/C	0.25	0.17	0.0001	1.65 (1.28-2.14)
rs11602520	77254720	C/T	0.07	0.10	0.1270	0.72 (0.47-1.09)
rs12797573	77255306	C/T	0.39	0.43	0.1415	0.85 (0.68-1.06)
rs7927173	77255548	G/A	0.07	0.10	0.1221	0.71 (0.47-1.09)
rs60842160	77257804	T/C	0.25	0.17	0.0001	1.65 (1.28-2.13)
rs10160677	77257805	A/G	0.07	0.10	0.1284	0.72 (0.47-1.09)
rs1540181	77259435	T/C	0.21	0.23	0.4329	0.90 (0.69-1.18)
rs1540180	77259574	C/T	0.21	0.23	0.4297	0.90 (0.69-1.18)

© 2019 Zhao S-X et al. *JAMA Network Open.*

rs11237216	77260742	A/G	0.46	0.49	0.1900	0.86 (0.69-1.08)
rs7943055	77261051	T/C	0.33	0.27	0.0297	1.30 (1.03-1.65)
rs4509797	77263203	G/A	0.07	0.10	0.1109	0.71 (0.47-1.08)
rs12290511	77266663	G/T	0.33	0.27	0.0292	1.30 (1.03-1.65)
rs10793255	77267611	G/A	0.07	0.10	0.0980	0.70 (0.46-1.06)
rs11237220	77268460	A/G	0.08	0.10	0.1388	0.73 (0.48-1.10)
rs7943742	77268856	T/C	0.33	0.27	0.0281	1.30 (1.03-1.65)
rs28451110	77269048	C/T	0.33	0.27	0.0270	1.31 (1.03-1.65)
rs6592730	77274203	C/T	0.33	0.27	0.0242	1.31 (1.04-1.66)
rs4366508	77274999	T/C	0.33	0.27	0.0242	1.31 (1.04-1.66)
rs4572159	77275337	C/T	0.21	0.23	0.3840	0.89 (0.68-1.16)
rs11512896	77275718	G/A	0.07	0.09	0.2188	0.77 (0.50-1.17)
rs4944164	77275849	A/G	0.33	0.27	0.0152	1.34 (1.06-1.70)
rs10899387	77276188	T/C	0.08	0.10	0.1322	0.73 (0.48-1.10)
rs12418261	77276462	A/G	0.25	0.17	0.0001	1.65 (1.28-2.13)
rs4945179	77278624	C/T	0.21	0.23	0.3810	0.89 (0.68-1.16)
rs68052959	77281589	G/T	0.25	0.17	0.0001	1.65 (1.28-2.14)
rs4553396	77282025	A/G	0.25	0.17	0.0002	1.65 (1.27-2.13)
rs2187547	77282081	A/G	0.25	0.17	0.0002	1.65 (1.27-2.13)
rs2156842	77282510	G/A	0.25	0.17	0.0002	1.65 (1.27-2.13)
rs4399350	77283055	T/C	0.25	0.17	0.0002	1.65 (1.27-2.13)
rs10899390	77283455	A/C	0.07	0.06	0.6029	1.12 (0.72-1.75)
rs11237226	77283606	G/A	0.07	0.06	0.6060	1.12 (0.72-1.74)
rs7950612	77283672	C/T	0.25	0.17	0.0002	1.65 (1.27-2.13)
rs7932111	77283687	A/G	0.25	0.17	0.0002	1.65 (1.27-2.13)
rs7933197	77283882	T/C	0.25	0.17	0.0002	1.65 (1.27-2.13)
rs4945182	77284886	A/G	0.25	0.17	0.0002	1.65 (1.27-2.13)
rs4945183	77285411	T/G	0.25	0.17	0.0001	1.65 (1.28-2.13)
rs12575305	77289090	T/C	0.25	0.17	0.0002	1.65 (1.27-2.13)
rs1945751	77289678	A/G	0.25	0.17	0.0002	1.65 (1.27-2.13)
rs12421695	77290439	A/G	0.25	0.17	0.0002	1.64 (1.27-2.12)
rs7947430	77290835	C/A	0.25	0.17	0.0002	1.64 (1.27-2.12)
rs67062156	77291457	C/T	0.25	0.17	0.0002	1.64 (1.27-2.11)
rs12574660	77292650	T/C	0.25	0.17	0.0002	1.64 (1.27-2.12)
rs59983642	77296055	T/C	0.25	0.17	0.0003	1.61 (1.24-2.09)
rs60849458	77296096	C/T	0.25	0.17	0.0002	1.64 (1.27-2.12)
rs67843465	77296319	T/C	0.25	0.17	0.0002	1.64 (1.27-2.12)
rs146238615	77297562	T/G	0.07	0.06	0.4953	1.16 (0.75-1.79)
rs113377845	77298886	G/A	0.25	0.17	0.0002	1.64 (1.27-2.13)
rs57588070	77299451	A/G	0.25	0.17	0.0002	1.64 (1.27-2.12)
rs11237230	77299627	T/C	0.07	0.06	0.4953	1.16 (0.75-1.79)
rs7129556	77300048	T/C	0.07	0.10	0.1597	0.74 (0.48-1.12)
rs2276415	77301341	A/G	0.25	0.17	0.0001	1.66 (1.28-2.14)
rs2276416	77301689	T/C	0.25	0.17	0.0001	1.65 (1.28-2.13)
rs59581090	77304482	C/T	0.25	0.17	0.0001	1.65 (1.28-2.13)
rs12577552	77305955	A/G	0.25	0.17	0.0001	1.66 (1.28-2.14)
rs6592736	77306254	T/C	0.25	0.17	0.0001	1.65 (1.28-2.13)
rs11237234	77309242	T/G	0.25	0.17	0.0001	1.66 (1.29-2.14)
rs10793257	77309533	C/T	0.33	0.27	0.0221	1.32 (1.04-1.67)
rs59969402	77309795	G/T	0.25	0.17	0.0001	1.66 (1.29-2.14)
rs7109398	77311154	A/G	0.25	0.17	9.92×10^{-5}	1.67 (1.29-2.15)
rs72945566	77311259	T/C	0.25	0.17	9.29×10^{-5}	1.67 (1.29-2.15)
rs11237236	77312790	A/G	0.08	0.10	0.1776	0.75 (0.50-1.13)
rs60132034	77312872	T/C	0.25	0.17	9.01×10^{-5}	1.67 (1.29-2.16)
rs713083	77313583	A/G	0.25	0.17	9.75×10^{-5}	1.67 (1.29-2.15)

© 2019 Zhao S-X et al. *JAMA Network Open.*

rs7930885	77314064	G/A	0.25	0.17	9.01×10^{-5}	1.67 (1.29-2.16)
rs7930988	77314133	G/A	0.25	0.17	9.75×10^{-5}	1.67 (1.29-2.15)
rs2032395	77314503	G/A	0.08	0.10	0.1549	0.74 (0.49-1.11)
rs9634032	77315214	C/T	0.22	0.23	0.4382	0.90 (0.69-1.18)
rs58608947	77316377	G/A	0.25	0.17	9.75×10^{-5}	1.67 (1.29-2.15)
rs66504136	77316721	A/C	0.25	0.17	9.29×10^{-5}	1.67 (1.29-2.15)
rs7106659	77317886	T/C	0.24	0.17	0.0004	1.61 (1.24-2.09)
rs4945185	77318142	A/G	0.33	0.27	0.0141	1.34 (1.06-1.70)
rs57805854	77320209	C/T	0.25	0.17	9.29×10^{-5}	1.67 (1.29-2.15)
rs4944171	77321312	C/T	0.25	0.17	9.01×10^{-5}	1.67 (1.29-2.16)
rs58720932	77322422	A/G	0.25	0.17	9.58×10^{-5}	1.67 (1.29-2.15)
rs11237240	77322805	A/G	0.07	0.06	0.3916	1.20 (0.78-1.84)
rs6592738	77323126	A/G	0.08	0.10	0.1829	0.75 (0.50-1.14)
rs3087668	77323370	A/G	0.46	0.50	0.1492	0.85 (0.68-1.06)
rs4945188	77323714	A/G	0.21	0.23	0.3726	0.89 (0.68-1.16)
rs10899394	77324261	G/A	0.08	0.10	0.2312	0.78 (0.52-1.17)
rs11822592	77325222	G/A	0.25	0.17	0.0001	1.67 (1.29-2.16)
rs61358482	77325584	G/T	0.25	0.17	9.89×10^{-5}	1.67 (1.29-2.15)
rs4945189	77331665	G/A	0.25	0.17	0.0001	1.66 (1.28-2.15)
rs4945190	77331688	C/A	0.25	0.17	0.0001	1.65 (1.28-2.14)
rs3781616	77333325	C/A	0.25	0.17	0.0001	1.65 (1.28-2.14)
rs66488360	77334083	A/C	0.25	0.17	0.0001	1.65 (1.28-2.14)
rs12225695	77334139	A/G	0.07	0.06	0.6342	1.11 (0.71-1.73)
rs11237243	77334532	A/G	0.07	0.06	0.6342	1.11 (0.71-1.73)
rs1540179	77334939	A/G	0.25	0.17	0.0001	1.65 (1.28-2.13)
rs11237244	77335336	G/A	0.08	0.10	0.2041	0.76 (0.50-1.15)
rs2013296	77335982	C/A	0.22	0.24	0.4278	0.90 (0.69-1.17)
rs7131169	77337009	C/A	0.25	0.17	0.0001	1.65 (1.28-2.13)
rs7131298	77337121	G/A	0.25	0.17	0.0001	1.65 (1.28-2.13)
rs60929773	77339667	G/A	0.25	0.17	0.0001	1.65 (1.28-2.13)
rs7946637	77339831	C/T	0.22	0.23	0.4576	0.91 (0.69-1.18)
rs67807487	77340123	T/C	0.25	0.17	0.0001	1.65 (1.28-2.13)
rs11237249	77341135	A/G	0.08	0.10	0.2174	0.77 (0.51-1.16)
rs7109527	77341306	A/G	0.25	0.17	0.0001	1.66 (1.29-2.15)
rs59459776	77341733	C/T	0.25	0.17	0.0001	1.66 (1.29-2.15)
rs61468764	77343507	A/C	0.25	0.17	0.0001	1.66 (1.29-2.15)
rs7925252	77346885	T/C	0.25	0.17	0.0001	1.65 (1.28-2.14)
rs7942838	77346961	C/T	0.25	0.17	0.0001	1.65 (1.28-2.14)
rs68013767	77349181	C/T	0.25	0.17	0.0001	1.66 (1.28-2.15)
rs59765258	77350211	T/C	0.25	0.17	0.0001	1.66 (1.29-2.15)
rs10466710	77350337	G/A	0.32	0.27	0.0195	1.33 (1.05-1.68)
rs113158303	77350759	T/C	0.25	0.17	0.0001	1.66 (1.29-2.15)
rs4945192	77350959	A/G	0.25	0.17	0.0001	1.66 (1.29-2.15)
rs12574999	77354007	C/T	0.25	0.16	8.43×10^{-5}	1.68 (1.30-2.18)
rs67881763	77355099	C/T	0.24	0.16	2.97×10^{-5}	1.75 (1.35-2.27)
rs57687790	77362010	T/C	0.24	0.16	4.57×10^{-5}	1.72 (1.33-2.24)
rs56097222	77362181	A/G	0.24	0.15	3.22×10^{-5}	1.74 (1.34-2.26)
rs7482006	77364942	G/A	0.24	0.16	4.57×10^{-5}	1.72 (1.33-2.24)
rs11604207	77367542	T/C	0.07	0.08	0.4504	0.85 (0.56-1.29)
rs72945598	77367738	T/C	0.07	0.08	0.4380	0.84 (0.55-1.28)
rs7939623	77370758	C/T	0.25	0.16	1.75×10^{-5}	1.77 (1.36-2.29)
rs7950631	77370897	T/C	0.25	0.16	1.75×10^{-5}	1.77 (1.36-2.29)
rs11237260	77372165	C/A	0.07	0.06	0.4374	1.18 (0.77-1.81)
rs2298820	77373414	G/T	0.25	0.16	1.81×10^{-5}	1.76 (1.36-2.28)
rs6592742	77376247	T/C	0.25	0.16	1.62×10^{-5}	1.77 (1.37-2.29)

© 2019 Zhao S-X et al. *JAMA Network Open.*

rs4945197	77378388	C/T	0.25	0.16	1.72×10^{-5}	1.77 (1.37-2.29)
rs28930682	77378505	G/A	0.25	0.16	1.72×10^{-5}	1.77 (1.37-2.29)
rs11237263	77378805	G/A	0.07	0.09	0.3782	0.82 (0.54-1.25)
rs11603438	77378894	A/G	0.07	0.08	0.4380	0.84 (0.55-1.28)
rs12575150	77379588	G/T	0.25	0.16	1.72×10^{-5}	1.77 (1.37-2.29)
rs7118516	77380234	T/C	0.25	0.16	1.72×10^{-5}	1.77 (1.37-2.29)
rs12418734	77380493	C/T	0.25	0.16	1.72×10^{-5}	1.77 (1.37-2.29)
rs59520338	77380865	G/A	0.25	0.16	1.72×10^{-5}	1.77 (1.37-2.29)
rs67035387	77381472	A/G	0.25	0.16	1.72×10^{-5}	1.77 (1.37-2.29)
rs11237264	77381751	C/T	0.32	0.24	0.0021	1.46 (1.15-1.85)
rs3781617	77385968	T/C	0.25	0.16	1.75×10^{-5}	1.77 (1.36-2.29)
rs12286317	77389020	C/T	0.07	0.09	0.3661	0.82 (0.54-1.25)
rs66681087	77390658	C/A	0.25	0.16	1.84×10^{-5}	1.76 (1.36-2.28)
rs17135800	77391684	A/C	0.24	0.16	2.65×10^{-5}	1.75 (1.35-2.27)
rs17135809	77395697	A/G	0.25	0.16	1.81×10^{-5}	1.76 (1.36-2.28)
rs17135811	77397059	G/A	0.25	0.16	1.75×10^{-5}	1.77 (1.36-2.29)
rs34446294	77398568	C/T	0.25	0.16	1.71×10^{-5}	1.77 (1.37-2.29)
rs12421699	77398676	C/T	0.25	0.16	1.71×10^{-5}	1.77 (1.37-2.29)
rs11237271	77399474	C/T	0.25	0.16	1.71×10^{-5}	1.77 (1.37-2.29)
rs72947625	77399602	T/G	0.07	0.08	0.4462	0.85 (0.55-1.29)
rs1544274	77402419	C/T	0.25	0.16	1.71×10^{-5}	1.77 (1.37-2.29)
rs1544272	77402734	A/C	0.25	0.16	1.71×10^{-5}	1.77 (1.37-2.29)
rs66991096	77405192	T/G	0.25	0.16	1.71×10^{-5}	1.77 (1.37-2.29)
rs66738005	77405567	C/T	0.25	0.16	1.71×10^{-5}	1.77 (1.37-2.29)
rs2186573	77405814	C/A	0.25	0.16	1.93×10^{-5}	1.76 (1.36-2.28)
rs67313689	77408279	A/G	0.25	0.16	1.91×10^{-5}	1.76 (1.36-2.28)
rs66472445	77408426	C/T	0.25	0.16	1.91×10^{-5}	1.76 (1.36-2.28)
rs67291795	77408877	T/C	0.25	0.16	1.91×10^{-5}	1.76 (1.36-2.28)
rs4945201	77409246	G/A	0.23	0.25	0.3638	0.89 (0.69-1.15)
rs4945202	77409407	T/C	0.25	0.16	1.91×10^{-5}	1.76 (1.36-2.28)
rs4945203	77409568	C/T	0.25	0.16	1.91×10^{-5}	1.76 (1.36-2.28)
rs10899399	77409832	T/C	0.07	0.08	0.4312	0.84 (0.55-1.28)
rs2212317	77410678	C/T	0.23	0.25	0.3664	0.89 (0.69-1.15)
rs7124753	77411181	G/A	0.25	0.16	1.91×10^{-5}	1.76 (1.36-2.28)
rs7950873	77412851	G/A	0.07	0.08	0.4421	0.84 (0.55-1.29)
rs67680371	77414382	C/T	0.25	0.16	1.99×10^{-5}	1.76 (1.36-2.28)
rs67555375	77415232	A/G	0.25	0.16	1.99×10^{-5}	1.76 (1.36-2.28)
rs67775318	77415542	A/G	0.25	0.16	1.99×10^{-5}	1.76 (1.36-2.28)
rs4145700	77415565	G/A	0.55	0.49	0.0503	1.25 (1.00-1.55)
rs72947653	77415574	C/T	0.25	0.16	1.99×10^{-5}	1.76 (1.36-2.28)
rs7940734	77416091	T/C	0.23	0.25	0.3721	0.89 (0.69-1.15)
rs66675536	77418702	T/C	0.25	0.16	2.02×10^{-5}	1.76 (1.36-2.28)
rs66924979	77418880	T/C	0.25	0.16	2.02×10^{-5}	1.76 (1.36-2.28)
rs496786	77420314	C/T	0.23	0.25	0.3777	0.89 (0.69-1.16)
rs11827357	77422635	T/C	0.25	0.16	1.81×10^{-5}	1.76 (1.36-2.28)
rs72947668	77428933	T/C	0.07	0.08	0.4490	0.85 (0.56-1.29)
rs7119471	77429953	G/A	0.25	0.16	2.02×10^{-5}	1.76 (1.36-2.28)
rs4531493	77430652	T/C	0.25	0.16	2.02×10^{-5}	1.76 (1.36-2.28)
rs10899401	77431334	A/G	0.32	0.24	0.0019	1.46 (1.15-1.85)
rs12418844	77431867	C/T	0.25	0.16	2.17×10^{-5}	1.75 (1.36-2.27)
rs7116671	77433527	C/T	0.25	0.16	2.17×10^{-5}	1.75 (1.36-2.27)
rs12575014	77434125	T/C	0.25	0.16	2.43×10^{-5}	1.75 (1.35-2.26)
rs66664368	77434261	C/T	0.25	0.16	2.36×10^{-5}	1.75 (1.35-2.27)
rs59404463	77434465	C/A	0.25	0.16	2.17×10^{-5}	1.75 (1.36-2.27)
rs4344516	77438059	G/A	0.07	0.08	0.4394	0.84 (0.55-1.28)

© 2019 Zhao S-X et al. *JAMA Network Open.*

rs56817084	77439495	G/A	0.25	0.16	2.17×10^{-5}	1.75 (1.36-2.27)
rs720383	77440106	G/A	0.25	0.16	2.17×10^{-5}	1.75 (1.36-2.27)
rs7109199	77441382	T/C	0.25	0.16	2.17×10^{-5}	1.75 (1.36-2.27)
rs2154988	77441591	T/C	0.25	0.16	2.17×10^{-5}	1.75 (1.36-2.27)
rs3949117	77441721	A/G	0.25	0.16	2.17×10^{-5}	1.75 (1.36-2.27)
rs75264226	77441973	G/A	0.25	0.16	2.17×10^{-5}	1.75 (1.36-2.27)
rs4945204	77443904	C/T	0.25	0.16	2.21×10^{-5}	1.75 (1.35-2.27)
rs4945205	77444469	T/C	0.25	0.16	2.17×10^{-5}	1.75 (1.36-2.27)
rs73491984	77446051	C/T	0.25	0.16	2.25×10^{-5}	1.75 (1.35-2.27)
rs77942147	77446117	T/C	0.25	0.16	2.25×10^{-5}	1.75 (1.35-2.27)
rs67769372	77446360	T/C	0.25	0.16	2.25×10^{-5}	1.75 (1.35-2.27)
rs7122968	77447054	G/T	0.06	0.08	0.1800	0.72 (0.45-1.16)
rs67724005	77447068	T/C	0.25	0.16	2.25×10^{-5}	1.75 (1.35-2.27)
rs28416524	77448616	C/T	0.23	0.25	0.4340	0.90 (0.70-1.17)
rs4945206	77452368	A/G	0.23	0.25	0.3848	0.89 (0.69-1.16)
rs11600998	77452790	C/T	0.06	0.08	0.3815	0.82 (0.52-1.27)
rs113465386	77453367	T/C	0.25	0.16	2.34×10^{-5}	1.75 (1.35-2.27)
rs12575416	77453386	A/G	0.07	0.06	0.5856	1.12 (0.74-1.72)
rs12294668	77454076	T/C	0.32	0.24	0.0018	1.47 (1.16-1.86)
rs60948958	77454257	G/A	0.25	0.16	2.34×10^{-5}	1.75 (1.35-2.27)
rs57362996	77454282	C/T	0.25	0.16	2.34×10^{-5}	1.75 (1.35-2.27)
rs10899402	77455166	T/C	0.23	0.25	0.4174	0.90 (0.69-1.17)
rs7924567	77455542	G/A	0.55	0.49	0.0443	1.25 (1.01-1.56)
rs11237279	77455583	G/A	0.32	0.24	0.0018	1.47 (1.16-1.86)
rs7927913	77455738	C/T	0.23	0.25	0.4174	0.90 (0.69-1.17)
rs10899403	77456343	C/T	0.23	0.25	0.4174	0.90 (0.69-1.17)
rs7120465	77456373	T/C	0.25	0.16	2.34×10^{-5}	1.75 (1.35-2.27)
rs17825097	77456786	C/T	0.07	0.06	0.5872	1.12 (0.73-1.72)
rs11237280	77456906	G/A	0.07	0.08	0.4744	0.85 (0.56-1.30)
rs7358471	77457616	T/C	0.23	0.25	0.5071	0.92 (0.71-1.19)
rs3819211	77457757	G/A	0.25	0.16	2.25×10^{-5}	1.75 (1.35-2.27)
rs58393579	77458907	T/G	0.25	0.16	2.25×10^{-5}	1.75 (1.35-2.27)
rs4322421	77459394	T/C	0.07	0.08	0.4504	0.85 (0.56-1.29)
rs7123204	77460614	G/A	0.25	0.16	2.25×10^{-5}	1.75 (1.35-2.27)
rs7118800	77461052	C/A	0.38	0.44	0.0260	0.78 (0.62-0.97)
rs10899404	77462792	T/C	0.07	0.08	0.4504	0.85 (0.56-1.29)
rs72949614	77463724	T/C	0.25	0.16	1.95×10^{-5}	1.76 (1.36-2.28)
rs4945209	77463991	C/T	0.25	0.16	2.25×10^{-5}	1.75 (1.35-2.27)
rs72949616	77464370	T/C	0.07	0.08	0.4744	0.85 (0.56-1.30)
rs72949618	77464530	A/G	0.07	0.08	0.4744	0.85 (0.56-1.30)
rs56218634	77464834	G/A	0.07	0.08	0.4744	0.85 (0.56-1.30)
rs7925343	77464936	T/C	0.23	0.25	0.4118	0.90 (0.69-1.16)
rs1986474	77466334	A/G	0.25	0.16	2.25×10^{-5}	1.75 (1.35-2.27)
rs537811	77468088	A/C	0.07	0.08	0.4339	0.84 (0.55-1.28)
rs11237284	77470067	C/A	0.23	0.25	0.3961	0.90 (0.69-1.16)
rs643388	77471042	T/C	0.07	0.08	0.4744	0.85 (0.56-1.30)
rs12420401	77471901	G/A	0.24	0.16	3.34×10^{-5}	1.74 (1.34-2.25)
rs10793262	77472138	T/C	0.23	0.25	0.4146	0.90 (0.69-1.17)
rs79574497	77473062	A/C	0.07	0.06	0.7028	1.09 (0.71-1.67)
rs12421391	77473209	C/T	0.25	0.16	2.34×10^{-5}	1.75 (1.35-2.27)
rs12419685	77473383	T/C	0.25	0.16	2.25×10^{-5}	1.75 (1.35-2.27)
rs1892869	77475506	T/C	0.25	0.16	2.34×10^{-5}	1.75 (1.35-2.27)
rs7951172	77476073	T/C	0.25	0.16	2.34×10^{-5}	1.75 (1.35-2.27)
rs1893901	77477828	C/T	0.25	0.16	2.82×10^{-5}	1.74 (1.34-2.25)
rs569072	77477956	A/G	0.34	0.40	0.0391	0.79 (0.63-0.99)

© 2019 Zhao S-X et al. *JAMA Network Open.*

rs7930066	77478108	A/G	0.25	0.16	2.87×10^{-5}	1.74 (1.34-2.25)
rs55881536	77478779	T/C	0.25	0.16	2.87×10^{-5}	1.74 (1.34-2.25)
rs72949656	77479090	T/C	0.24	0.16	7.40×10^{-5}	1.70 (1.31-2.21)
rs4269952	77479264	A/G	0.24	0.16	5.79×10^{-5}	1.71 (1.32-2.22)
rs4578411	77479293	C/T	0.24	0.16	4.34×10^{-5}	1.73 (1.33-2.25)
rs67230007	77480543	G/T	0.25	0.16	2.87×10^{-5}	1.74 (1.34-2.25)
rs10899406	77482773	G/A	0.23	0.25	0.3925	0.89 (0.69-1.16)
rs516529	77484735	T/C	0.38	0.44	0.0228	0.77 (0.62-0.97)
rs10899407	77487163	G/A	0.23	0.25	0.3979	0.90 (0.69-1.16)
rs2154973	77488809	T/C	0.25	0.16	2.98×10^{-5}	1.74 (1.34-2.25)
rs2154972	77488825	A/G	0.07	0.08	0.4504	0.85 (0.56-1.29)
rs12364072	77489608	G/T	0.23	0.25	0.3979	0.90 (0.69-1.16)
rs4945210	77489725	T/C	0.25	0.16	2.87×10^{-5}	1.74 (1.34-2.25)
rs11237289	77489750	G/T	0.23	0.25	0.3979	0.90 (0.69-1.16)
rs4945211	77489851	A/C	0.25	0.16	2.98×10^{-5}	1.74 (1.34-2.25)
rs585513	77490850	T/C	0.38	0.44	0.0234	0.77 (0.62-0.97)
rs10899409	77490958	A/G	0.23	0.25	0.3353	0.88 (0.68-1.14)
rs7119665	77491864	A/C	0.25	0.16	1.52×10^{-5}	1.77 (1.37-2.30)
rs484363	77492594	G/A	0.07	0.08	0.4964	0.86 (0.56-1.31)
rs74480381	77494004	C/T	0.07	0.06	0.5877	1.12 (0.73-1.72)
rs684813	77494754	A/G	0.07	0.08	0.5129	0.87 (0.57-1.32)
rs7105526	77495156	A/C	0.25	0.16	2.98×10^{-5}	1.74 (1.34-2.25)
rs7106113	77495645	T/C	0.25	0.16	2.98×10^{-5}	1.74 (1.34-2.25)
rs11825573	77495896	G/A	0.25	0.16	2.98×10^{-5}	1.74 (1.34-2.25)
rs72931818	77496172	T/C	0.24	0.16	7.90×10^{-5}	1.70 (1.31-2.21)
rs2508189	77496567	T/C	0.07	0.09	0.4229	0.84 (0.55-1.28)
rs7936904	77497747	T/C	0.25	0.16	2.98×10^{-5}	1.74 (1.34-2.25)
rs11237295	77498274	T/C	0.23	0.25	0.4055	0.90 (0.69-1.16)
rs650171	77498486	C/T	0.07	0.08	0.4504	0.85 (0.56-1.29)
rs67200285	77498608	T/C	0.25	0.16	2.98×10^{-5}	1.74 (1.34-2.25)
rs72931829	77498615	C/T	0.25	0.16	2.98×10^{-5}	1.74 (1.34-2.25)
rs648842	77498768	C/T	0.55	0.49	0.0467	1.25 (1.00-1.56)
rs7123080	77499387	T/C	0.07	0.08	0.4504	0.85 (0.56-1.29)
rs60186049	77499534	C/A	0.25	0.16	2.98×10^{-5}	1.74 (1.34-2.25)
rs7127803	77500736	T/C	0.25	0.16	2.98×10^{-5}	1.74 (1.34-2.25)
rs4944175	77501103	G/A	0.25	0.16	2.98×10^{-5}	1.74 (1.34-2.25)
rs4945212	77501983	G/A	0.25	0.16	2.98×10^{-5}	1.74 (1.34-2.25)
rs4945213	77502132	T/C	0.25	0.16	2.98×10^{-5}	1.74 (1.34-2.25)
rs4945215	77502519	C/A	0.25	0.16	2.98×10^{-5}	1.74 (1.34-2.25)
rs67241586	77502632	C/T	0.25	0.16	2.98×10^{-5}	1.74 (1.34-2.25)
rs12418152	77504709	T/C	0.25	0.16	2.98×10^{-5}	1.74 (1.34-2.25)
rs61201484	77505479	T/C	0.25	0.16	2.98×10^{-5}	1.74 (1.34-2.25)
rs7931215	77506044	T/C	0.25	0.16	2.98×10^{-5}	1.74 (1.34-2.25)
rs11828664	77507198	A/G	0.25	0.16	2.87×10^{-5}	1.74 (1.34-2.25)
rs11824150	77507211	T/C	0.25	0.16	2.98×10^{-5}	1.74 (1.34-2.25)
rs144539886	77507420	T/G	0.25	0.16	2.98×10^{-5}	1.74 (1.34-2.25)
rs151245276	77508213	T/C	0.25	0.16	2.98×10^{-5}	1.74 (1.34-2.25)
rs11518811	77508592	A/G	0.23	0.25	0.4007	0.90 (0.69-1.16)
rs12420092	77509417	T/C	0.25	0.16	1.85×10^{-5}	1.76 (1.36-2.28)
rs7947859	77510547	T/C	0.25	0.16	2.74×10^{-5}	1.74 (1.35-2.26)
rs68146267	77513388	C/T	0.25	0.16	2.98×10^{-5}	1.74 (1.34-2.25)
rs7941418	77514216	T/G	0.25	0.16	2.98×10^{-5}	1.74 (1.34-2.25)
rs11237299	77514431	A/G	0.23	0.25	0.3684	0.89 (0.69-1.15)
rs113217620	77514434	G/T	0.25	0.16	2.98×10^{-5}	1.74 (1.34-2.25)
rs681411	77515230	T/C	0.55	0.49	0.0477	1.25 (1.00-1.56)

© 2019 Zhao S-X et al. *JAMA Network Open.*

rs4469913	77517812	A/C	0.25	0.16	1.75×10^{-5}	1.77 (1.37-2.29)
rs12417488	77518680	G/A	0.25	0.16	2.98×10^{-5}	1.74 (1.34-2.25)
rs12418476	77520211	C/T	0.25	0.16	2.98×10^{-5}	1.74 (1.34-2.25)
rs7113415	77521390	G/A	0.25	0.16	2.59×10^{-5}	1.75 (1.35-2.26)
rs9988883	77522234	T/C	0.23	0.25	0.4029	0.90 (0.69-1.16)
rs650636	77522633	G/A	0.55	0.49	0.0467	1.25 (1.00-1.56)
rs59191974	77522677	T/C	0.25	0.16	2.98×10^{-5}	1.74 (1.34-2.25)
rs111692767	77522977	T/G	0.25	0.16	2.98×10^{-5}	1.74 (1.34-2.25)
rs4945217	77523084	C/T	0.25	0.16	1.85×10^{-5}	1.76 (1.36-2.28)
rs4945219	77523484	T/C	0.25	0.16	2.98×10^{-5}	1.74 (1.34-2.25)
rs58914857	77524722	G/T	0.25	0.16	2.98×10^{-5}	1.74 (1.34-2.25)
rs618535	77525203	G/A	0.55	0.49	0.0467	1.25 (1.00-1.56)
rs112873821	77525310	A/G	0.25	0.16	3.03×10^{-5}	1.74 (1.34-2.25)
rs28610388	77525654	G/A	0.25	0.16	2.98×10^{-5}	1.74 (1.34-2.25)
rs113740785	77525738	A/G	0.25	0.16	2.98×10^{-5}	1.74 (1.34-2.25)
rs637605	77525936	T/G	0.07	0.06	0.5472	1.14 (0.74-1.74)
rs12417115	77526474	A/C	0.38	0.44	0.0169	0.76 (0.61-0.95)
rs10793264	77528339	C/T	0.23	0.25	0.4029	0.90 (0.69-1.16)
rs623173	77529528	C/T	0.07	0.08	0.5038	0.86 (0.57-1.31)
rs67875187	77530953	C/T	0.25	0.16	2.98×10^{-5}	1.74 (1.34-2.25)
rs11820337	77531890	C/T	0.55	0.49	0.0467	1.25 (1.00-1.56)
rs78196556	77532306	T/C	0.03	0.04	0.5168	0.82 (0.45-1.49)
rs67606281	77532463	C/T	0.25	0.16	2.98×10^{-5}	1.74 (1.34-2.25)
rs4245453	77533652	T/G	0.23	0.25	0.3877	0.89 (0.69-1.16)
rs7937983	77534355	G/A	0.25	0.16	2.98×10^{-5}	1.74 (1.34-2.25)
rs7951033	77534444	G/A	0.07	0.08	0.4504	0.85 (0.56-1.29)
rs35711367	77536441	C/T	0.32	0.24	0.0020	1.46 (1.15-1.85)
rs12417232	77539085	A/G	0.25	0.16	2.98×10^{-5}	1.74 (1.34-2.25)
rs7126909	77540949	T/C	0.25	0.16	2.92×10^{-5}	1.74 (1.34-2.25)
rs17135905	77545870	C/A	0.25	0.16	3.03×10^{-5}	1.74 (1.34-2.25)
rs12417760	77551096	A/G	0.25	0.16	2.98×10^{-5}	1.74 (1.34-2.25)
rs12422061	77551303	G/A	0.03	0.04	0.3193	0.72 (0.38-1.38)
rs648896	77551500	T/C	0.07	0.08	0.4952	0.86 (0.56-1.31)
rs4944176	77552987	C/T	0.25	0.16	2.98×10^{-5}	1.74 (1.34-2.25)
rs621456	77553030	T/C	0.07	0.08	0.4952	0.86 (0.56-1.31)
rs585721	77553638	C/T	0.32	0.24	0.0022	1.46 (1.15-1.84)
rs601551	77554821	A/G	0.32	0.24	0.0021	1.46 (1.15-1.85)
rs7110322	77554945	T/G	0.25	0.16	2.98×10^{-5}	1.74 (1.34-2.25)
rs1790246	77555356	A/G	0.23	0.25	0.4662	0.91 (0.70-1.18)
rs634477	77559011	A/G	0.45	0.50	0.0576	0.81 (0.65-1.01)
rs1793340	77560323	A/G	0.45	0.50	0.0576	0.81 (0.65-1.01)
rs598071	77560652	T/G	0.07	0.08	0.4504	0.85 (0.56-1.29)
rs2508184	77562033	A/G	0.32	0.24	0.0019	1.46 (1.15-1.85)
rs694893	77563344	T/C	0.32	0.24	0.0019	1.46 (1.15-1.85)
rs12418551	77565877	T/C	0.25	0.16	2.77×10^{-5}	1.74 (1.35-2.25)
rs12420290	77566054	G/A	0.25	0.16	2.77×10^{-5}	1.74 (1.35-2.25)
rs647235	77566849	G/A	0.32	0.24	0.0019	1.46 (1.15-1.85)
rs24356	77567528	T/C	0.07	0.08	0.4504	0.85 (0.56-1.29)
rs3886195	77567584	A/G	0.25	0.16	2.77×10^{-5}	1.74 (1.35-2.25)
rs685474	77567681	T/C	0.32	0.24	0.0019	1.46 (1.15-1.85)
rs12285765	77569035	T/C	0.07	0.08	0.4504	0.85 (0.56-1.29)
rs4491258	77569110	T/G	0.25	0.16	2.77×10^{-5}	1.74 (1.35-2.25)
rs600968	77569784	C/T	0.32	0.24	0.0019	1.46 (1.15-1.85)
rs1783544	77572468	T/C	0.07	0.08	0.4732	0.85 (0.56-1.30)
rs636971	77574249	T/C	0.07	0.08	0.5561	0.88 (0.58-1.34)

© 2019 Zhao S-X et al. *JAMA Network Open.*

rs584732	77574605	T/C	0.32	0.24	0.0019	1.46 (1.16-1.86)
rs76393422	77574910	A/G	0.03	0.04	0.3441	0.73 (0.38-1.40)
rs599962	77575639	T/C	0.32	0.24	0.0019	1.46 (1.15-1.85)
rs12574507	77575641	A/G	0.25	0.16	2.77×10^{-5}	1.74 (1.35-2.25)
rs1793341	77575977	T/C	0.23	0.25	0.3799	0.89 (0.69-1.16)
rs628668	77577494	T/C	0.07	0.08	0.4504	0.85 (0.56-1.29)
rs603462	77577864	A/C	0.32	0.24	0.0019	1.46 (1.15-1.85)
rs694845	77579747	G/T	0.07	0.08	0.4644	0.85 (0.56-1.30)
rs667213	77580995	A/G	0.32	0.24	0.0019	1.46 (1.15-1.85)
rs4448713	77581444	A/G	0.25	0.16	2.77×10^{-5}	1.74 (1.35-2.25)
rs113853065	77581644	G/A	0.25	0.16	2.77×10^{-5}	1.74 (1.35-2.25)
rs591283	77582743	T/C	0.32	0.24	0.0019	1.46 (1.15-1.85)
rs2186564	77583266	A/G	0.25	0.16	2.40×10^{-5}	1.75 (1.35-2.27)
rs588217	77583376	A/G	0.07	0.08	0.4504	0.85 (0.56-1.29)
rs668701	77583476	A/G	0.07	0.08	0.5042	0.86 (0.57-1.32)
rs667225	77583855	C/A	0.07	0.08	0.4504	0.85 (0.56-1.29)
rs611671	77584138	T/C	0.32	0.24	0.0018	1.47 (1.16-1.86)
rs1783546	77584647	T/C	0.23	0.25	0.4191	0.90 (0.69-1.17)
rs598933	77586358	A/G	0.32	0.24	0.0018	1.47 (1.16-1.86)
rs636041	77586409	A/G	0.32	0.24	0.0018	1.47 (1.16-1.86)
rs648601	77586910	G/A	0.07	0.08	0.4490	0.85 (0.56-1.29)
rs616354	77587973	T/C	0.56	0.49	0.0235	1.29 (1.04-1.61)
rs72939439	77589649	G/A	0.24	0.16	3.26×10^{-5}	1.74 (1.34-2.25)
rs663674	77592522	G/A	0.07	0.08	0.4449	0.84 (0.55-1.29)
rs947841	77592909	A/G	0.07	0.08	0.4312	0.84 (0.55-1.28)
rs4945223	77592945	G/T	0.25	0.16	2.56×10^{-5}	1.75 (1.35-2.26)
rs4944178	77593221	T/C	0.25	0.16	2.56×10^{-5}	1.75 (1.35-2.26)
rs60208812	77593347	G/A	0.25	0.16	2.56×10^{-5}	1.75 (1.35-2.26)
rs695046	77594354	G/A	0.07	0.09	0.4179	0.84 (0.55-1.27)
rs2051484	77595080	T/C	0.24	0.16	5.94×10^{-5}	1.72 (1.32-2.23)
rs608389	77595642	G/A	0.32	0.24	0.0020	1.46 (1.15-1.85)
rs658398	77595717	T/C	0.32	0.24	0.0020	1.46 (1.15-1.85)
rs687037	77596104	A/C	0.07	0.08	0.4114	0.83 (0.54-1.28)
rs581807	77596773	A/G	0.32	0.24	0.0021	1.46 (1.15-1.85)
rs108843	77597486	T/C	0.32	0.24	0.0019	1.46 (1.16-1.86)
rs141335075	77599576	C/T	0.25	0.16	1.62×10^{-5}	1.77 (1.37-2.29)
rs10899414	77599916	A/G	0.32	0.24	0.0014	1.48 (1.17-1.87)
rs113671826	77600361	G/A	0.25	0.16	1.88×10^{-5}	1.76 (1.36-2.28)
rs670090	77602673	G/A	0.07	0.08	0.4823	0.86 (0.56-1.30)
rs4245455	77603520	C/T	0.25	0.16	1.02×10^{-5}	1.79 (1.39-2.32)
rs1790244	77617950	G/A	0.37	0.40	0.2684	0.88 (0.70-1.10)
rs1793344	77618066	T/C	0.37	0.40	0.2624	0.88 (0.70-1.10)
rs1790245	77618441	A/G	0.37	0.40	0.2678	0.88 (0.70-1.10)
rs1790256	77620340	C/T	0.37	0.40	0.2612	0.88 (0.70-1.10)
rs10899415	77623873	C/T	0.38	0.40	0.2852	0.88 (0.71-1.11)
rs1793331	77625655	C/T	0.38	0.40	0.2909	0.89 (0.71-1.11)
rs1793338	77651786	C/T	0.38	0.40	0.3234	0.89 (0.71-1.12)
rs7115801	77661518	A/G	0.12	0.14	0.3175	0.84 (0.60-1.18)
rs12789522	77661805	G/A	0.38	0.40	0.3695	0.90 (0.72-1.13)
rs112788380	77662362	T/G	0.12	0.14	0.3175	0.84 (0.60-1.18)
rs12279880	77666487	T/C	0.12	0.14	0.2908	0.83 (0.59-1.17)
rs11826938	77667682	T/C	0.50	0.45	0.1152	1.19 (0.96-1.49)
rs11559637	77667979	A/G	0.37	0.40	0.2998	0.89 (0.71-1.11)
rs11501934	77668019	T/C	0.12	0.14	0.2639	0.82 (0.59-1.15)
rs4123593	77669985	G/A	0.12	0.14	0.2375	0.81 (0.58-1.14)

© 2019 Zhao S-X et al. *JAMA Network Open.*

rs4123595	77670112	G/A	0.12	0.14	0.2375	0.81 (0.58-1.14)
rs1823507	77670237	T/C	0.12	0.14	0.2375	0.81 (0.58-1.14)
rs72939500	77671217	G/A	0.12	0.14	0.2343	0.81 (0.58-1.14)
rs12796422	77671281	G/A	0.38	0.41	0.4215	0.91 (0.73-1.14)
rs2305317	77671544	C/T	0.12	0.14	0.2331	0.81 (0.58-1.14)
rs7929510	77671868	C/T	0.50	0.45	0.0918	1.21 (0.97-1.50)
rs7950529	77672314	C/T	0.38	0.40	0.3801	0.90 (0.72-1.13)
rs7115539	77673840	T/C	0.38	0.40	0.3413	0.90 (0.72-1.12)
rs9630225	77675723	A/G	0.12	0.14	0.2396	0.81 (0.58-1.14)
rs6592751	77675945	C/T	0.38	0.40	0.3938	0.91 (0.72-1.14)
rs11237338	77678541	T/C	0.12	0.14	0.2454	0.82 (0.58-1.14)
rs12807406	77680399	T/G	0.30	0.34	0.1667	0.85 (0.67-1.07)
rs2187564	77682279	A/C	0.37	0.40	0.3142	0.89 (0.71-1.12)
rs58992562	77683319	G/A	0.26	0.20	0.0041	1.45 (1.12-1.86)
rs12799701	77684136	T/C	0.12	0.14	0.2449	0.82 (0.58-1.14)
rs72941328	77684948	T/C	0.26	0.20	0.0042	1.45 (1.12-1.86)
rs7101610	77685224	T/C	0.51	0.46	0.0735	1.22 (0.98-1.52)
rs72941332	77685936	A/G	0.12	0.14	0.2339	0.81 (0.58-1.14)
rs11823006	77685996	C/T	0.38	0.40	0.3821	0.90 (0.72-1.13)
rs10899419	77686775	A/G	0.38	0.40	0.4217	0.91 (0.73-1.14)
rs72941334	77687198	C/T	0.26	0.20	0.0042	1.45 (1.12-1.86)
rs11237341	77687737	A/G	0.12	0.14	0.2526	0.82 (0.59-1.15)
rs72941336	77688244	T/C	0.26	0.20	0.0042	1.45 (1.12-1.86)
rs72941337	77688441	A/C	0.26	0.20	0.0042	1.45 (1.12-1.86)
rs10899420	77688774	A/G	0.51	0.46	0.0727	1.22 (0.98-1.52)
rs72941339	77688940	G/T	0.12	0.14	0.2600	0.82 (0.59-1.15)
rs12289316	77689297	T/G	0.12	0.15	0.2333	0.81 (0.58-1.14)
rs3740681	77690426	C/T	0.26	0.20	0.0052	1.44 (1.11-1.85)
rs17825404	77692775	T/G	0.12	0.14	0.2383	0.81 (0.58-1.14)
rs11237342	77693174	T/C	0.07	0.06	0.3759	1.21 (0.79-1.87)
rs10793267	77693272	C/T	0.50	0.45	0.0900	1.21 (0.97-1.51)
rs11237343	77693435	T/C	0.50	0.45	0.0925	1.21 (0.97-1.50)
rs72941350	77693579	T/C	0.26	0.20	0.0042	1.45 (1.12-1.86)
rs6592754	77694362	A/G	0.50	0.45	0.0881	1.21 (0.97-1.51)
rs11601908	77694544	T/C	0.38	0.40	0.3856	0.91 (0.72-1.14)
rs72941353	77695894	T/C	0.12	0.14	0.2370	0.81 (0.58-1.14)
rs2016147	77705536	A/C	0.25	0.18	0.0043	1.46 (1.13-1.89)
rs921383	77710841	A/G	0.29	0.30	0.6945	0.95 (0.75-1.21)
rs11237353	77714746	A/C	0.31	0.25	0.0176	1.34 (1.06-1.70)
rs17136094	77715627	C/T	0.10	0.12	0.1711	0.77 (0.54-1.11)
rs56360479	77718711	T/C	0.10	0.12	0.2561	0.81 (0.56-1.16)
rs10899425	77718712	A/G	0.31	0.25	0.0268	1.32 (1.04-1.67)
rs11601778	77719708	C/T	0.32	0.26	0.0200	1.33 (1.05-1.68)
rs12577359	77723797	T/C	0.32	0.27	0.0307	1.30 (1.03-1.65)
rs7930865	77724674	T/C	0.42	0.39	0.2539	1.14 (0.91-1.42)
rs72943018	77725515	T/C	0.10	0.12	0.2118	0.79 (0.54-1.14)
rs73503585	77726979	C/T	0.10	0.12	0.1917	0.78 (0.54-1.13)
rs72943021	77727038	T/C	0.10	0.12	0.2020	0.78 (0.54-1.13)
rs3824865	77727379	C/T	0.10	0.12	0.1921	0.78 (0.54-1.13)
rs57903322	77731160	A/C	0.05	0.08	0.0352	0.59 (0.36-0.97)
rs7937238	77731416	C/T	0.51	0.49	0.6216	1.06 (0.85-1.32)
rs2034500	77731755	C/T	0.51	0.49	0.6157	1.06 (0.85-1.32)
rs11237359	77732637	C/A	0.45	0.41	0.1452	1.18 (0.94-1.47)

© 2019 Zhao S-X et al. *JAMA Network Open.*

SNP, single nucleotide polymorphism; BP, base position; MAF, minor allele frequency; TPP, thyrotoxic hypokalemic periodic paralysis; OR, odds ratio; CI, confidence interval.

© 2019 Zhao S-X et al. *JAMA Network Open.*

eTable 7. TPP association results for the imputed and typed SNPs in the 17q24.3 region in the initial genome-wide scan						
SNP	BP	Alleles	MAF_TPP	MAF_Controls	*P*	OR (95%CI)
rs11077471	68085547	T/G	0.05	0.04	0.3144	1.31 (0.77-2.22)
rs12051811	68085573	T/G	0.05	0.04	0.3253	1.30 (0.77-2.21)
rs11077472	68085673	C/T	0.05	0.04	0.3253	1.30 (0.77-2.21)
rs7209824	68085815	C/T	0.05	0.04	0.3253	1.30 (0.77-2.21)
rs2366290	68086796	C/T	0.05	0.04	0.2358	1.36 (0.82-2.28)
rs9911757	68087687	C/T	0.05	0.04	0.1780	1.41 (0.85-2.32)
rs8072570	68087909	A/G	0.05	0.04	0.1786	1.41 (0.85-2.32)
rs8072498	68088221	C/T	0.05	0.04	0.1780	1.41 (0.85-2.32)
rs8077474	68088505	T/C	0.05	0.04	0.1786	1.41 (0.85-2.32)
rs8182292	68088567	C/A	0.05	0.04	0.1798	1.40 (0.85-2.31)
rs8182294	68088612	C/T	0.05	0.04	0.1780	1.41 (0.85-2.32)
rs12709248	68088771	C/T	0.05	0.04	0.1930	1.39 (0.84-2.29)
rs12709249	68088884	A/G	0.05	0.04	0.1930	1.39 (0.84-2.29)
rs12709250	68088953	G/A	0.05	0.04	0.1937	1.39 (0.84-2.29)
rs4433847	68089273	G/A	0.05	0.04	0.1930	1.39 (0.84-2.29)
rs9892289	68089452	T/C	0.05	0.04	0.1930	1.39 (0.84-2.29)
rs2886632	68089902	T/G	0.05	0.04	0.1930	1.39 (0.84-2.29)
rs7207387	68089989	A/G	0.04	0.03	0.4901	1.23 (0.69-2.19)
rs7207528	68090053	A/G	0.05	0.04	0.1930	1.39 (0.84-2.29)
rs6501367	68090128	T/G	0.05	0.04	0.1930	1.39 (0.84-2.29)
rs6501368	68090181	T/G	0.05	0.04	0.1930	1.39 (0.84-2.29)
rs11867479	68090207	C/T	0.43	0.43	0.9199	1.01 (0.81-1.26)
rs6501369	68090269	C/T	0.05	0.04	0.1930	1.39 (0.84-2.29)
rs6501370	68090318	G/A	0.05	0.04	0.1930	1.39 (0.84-2.29)
rs8065574	68090615	A/G	0.05	0.04	0.1930	1.39 (0.84-2.29)
rs8082547	68090671	G/T	0.05	0.04	0.1930	1.39 (0.84-2.29)
rs8069797	68090674	T/C	0.05	0.04	0.1930	1.39 (0.84-2.29)
rs8069195	68090846	C/T	0.05	0.04	0.2015	1.38 (0.84-2.28)
rs8069571	68091130	C/A	0.04	0.03	0.4901	1.23 (0.69-2.19)
rs8070321	68091225	A/C	0.05	0.04	0.1930	1.39 (0.84-2.29)
rs8070915	68091561	A/G	0.05	0.04	0.1930	1.39 (0.84-2.29)
rs8069216	68091598	G/A	0.05	0.04	0.1930	1.39 (0.84-2.29)
rs8069368	68091654	G/A	0.05	0.04	0.1930	1.39 (0.84-2.29)
rs8070530	68091676	C/T	0.05	0.04	0.1930	1.39 (0.84-2.29)
rs8075045	68091932	A/C	0.05	0.04	0.1488	1.43 (0.88-2.32)
rs8074582	68091952	C/A	0.05	0.04	0.2009	1.38 (0.84-2.28)
rs12942690	68092322	C/A	0.05	0.04	0.2015	1.38 (0.84-2.28)
rs2190607	68092752	T/C	0.05	0.04	0.2009	1.38 (0.84-2.28)
rs7224070	68093044	A/G	0.05	0.04	0.2009	1.38 (0.84-2.28)
rs7222323	68093060	G/T	0.05	0.04	0.2009	1.38 (0.84-2.28)
rs7223856	68093286	C/T	0.05	0.04	0.1290	1.45 (0.89-2.37)
rs11077473	68093353	C/T	0.05	0.04	0.1295	1.45 (0.89-2.37)
rs11077474	68093376	A/G	0.04	0.03	0.3479	1.31 (0.75-2.29)
rs11077476	68093427	T/C	0.05	0.04	0.1290	1.45 (0.89-2.37)
rs10221235	68093912	T/C	0.05	0.04	0.2109	1.37 (0.83-2.26)
rs10221237	68094026	T/C	0.05	0.04	0.1290	1.45 (0.89-2.37)

© 2019 Zhao S-X et al. *JAMA Network Open.*

rs9302911	68094230	C/T	0.04	0.03	0.3479	1.31 (0.75-2.29)
rs724824	68095619	G/T	0.04	0.03	0.3479	1.31 (0.75-2.29)
rs724825	68095623	T/G	0.04	0.03	0.3479	1.31 (0.75-2.29)
rs6501375	68096658	T/C	0.04	0.03	0.3479	1.31 (0.75-2.29)
rs7226242	68096908	A/G	0.05	0.04	0.1290	1.45 (0.89-2.37)
rs7226144	68097181	C/T	0.04	0.03	0.3479	1.31 (0.75-2.29)
rs9890060	68102331	C/T	0.40	0.39	0.6835	1.05 (0.84-1.31)
rs4968796	68103347	C/T	0.38	0.38	0.9674	1.00 (0.79-1.25)
rs3785902	68108118	C/T	0.36	0.40	0.2099	0.86 (0.69-1.09)
rs12936361	68108417	C/T	0.34	0.31	0.3941	1.11 (0.88-1.40)
rs8079768	68114959	C/T	0.23	0.19	0.1203	1.23 (0.95-1.60)
rs9909905	68115346	T/C	0.23	0.19	0.0635	1.28 (0.99-1.66)
rs9897883	68116951	G/A	0.23	0.19	0.1240	1.23 (0.95-1.61)
rs9897311	68117095	T/C	0.23	0.19	0.1186	1.24 (0.95-1.61)
rs12943869	68120806	A/G	0.43	0.50	0.0140	0.75 (0.61-0.94)
rs3826428	68131048	G/T	0.14	0.11	0.1144	1.30 (0.94-1.79)
rs6788	68131526	T/C	0.13	0.09	0.0433	1.41 (1.01-1.96)
rs2366489	68133764	T/C	0.14	0.11	0.0861	1.32 (0.96-1.81)
rs2158967	68133865	T/C	0.14	0.11	0.0705	1.34 (0.97-1.84)
rs17702078	68134618	C/T	0.14	0.11	0.0709	1.33 (0.97-1.83)
rs7217293	68135294	C/T	0.14	0.11	0.0978	1.30 (0.95-1.79)
rs7217947	68135696	C/T	0.14	0.11	0.0950	1.31 (0.95-1.79)
rs4405595	68135740	T/C	0.14	0.11	0.0950	1.31 (0.95-1.79)
rs7208531	68137138	C/T	0.14	0.11	0.0967	1.30 (0.95-1.79)
rs16975251	68137167	A/G	0.31	0.27	0.1292	1.20 (0.95-1.52)
rs67891720	68137788	G/A	0.31	0.27	0.1355	1.20 (0.94-1.52)
rs71375771	68138343	T/G	0.14	0.11	0.0950	1.31 (0.95-1.79)
rs9912069	68138833	G/A	0.14	0.11	0.0950	1.31 (0.95-1.79)
rs9896573	68139044	C/T	0.14	0.11	0.0893	1.31 (0.96-1.80)
rs9788982	68139785	G/A	0.14	0.11	0.1059	1.30 (0.94-1.79)
rs6501379	68140040	C/T	0.14	0.11	0.1070	1.30 (0.94-1.78)
rs71375772	68141674	C/T	0.14	0.11	0.1026	1.30 (0.94-1.79)
rs34237612	68142113	C/T	0.14	0.11	0.1026	1.30 (0.94-1.79)
rs35391414	68142724	T/C	0.14	0.11	0.1026	1.30 (0.94-1.79)
rs28415525	68143434	G/A	0.14	0.11	0.1049	1.30 (0.94-1.79)
rs4968884	68143989	T/C	0.14	0.11	0.1049	1.30 (0.94-1.79)
rs4968798	68144096	C/T	0.14	0.11	0.1049	1.30 (0.94-1.79)
rs12451657	68146450	T/C	0.14	0.11	0.1087	1.29 (0.94-1.78)
rs34053437	68146727	T/C	0.14	0.11	0.1087	1.29 (0.94-1.78)
rs12945558	68147069	T/C	0.14	0.11	0.1094	1.29 (0.94-1.78)
rs12946552	68147438	T/C	0.14	0.11	0.1094	1.29 (0.94-1.78)
rs34136246	68147587	C/T	0.14	0.11	0.1094	1.29 (0.94-1.78)
rs8081486	68147928	T/C	0.14	0.11	0.1078	1.30 (0.94-1.78)
rs8082195	68148034	G/A	0.14	0.11	0.1094	1.29 (0.94-1.78)
rs8067870	68148119	G/T	0.14	0.11	0.1094	1.29 (0.94-1.78)
rs6501381	68148350	C/T	0.14	0.11	0.1138	1.29 (0.94-1.78)
rs12449606	68148811	A/C	0.14	0.11	0.1276	1.28 (0.93-1.76)
rs12452512	68149139	C/A	0.14	0.11	0.1250	1.28 (0.93-1.75)
rs16975266	68149253	G/A	0.14	0.11	0.1234	1.28 (0.93-1.76)
rs12453808	68150722	C/T	0.14	0.11	0.1226	1.28 (0.93-1.76)
rs9915368	68151112	G/A	0.14	0.11	0.1226	1.28 (0.93-1.76)
rs77388117	68151469	T/C	0.14	0.11	0.1458	1.26 (0.92-1.74)

© 2019 Zhao S-X et al. *JAMA Network Open.*

rs12952229	68151736	T/C	0.14	0.11	0.1226	1.28 (0.93-1.76)
rs1558100	68152714	T/C	0.14	0.11	0.1226	1.28 (0.93-1.76)
rs7215060	68153664	T/C	0.14	0.11	0.1173	1.29 (0.94-1.76)
rs3944089	68155155	T/C	0.14	0.11	0.1226	1.28 (0.93-1.76)
rs28688305	68157325	A/G	0.14	0.11	0.1226	1.28 (0.93-1.76)
rs75653467	68157524	C/T	0.14	0.11	0.1458	1.26 (0.92-1.74)
rs2058063	68157840	C/T	0.14	0.11	0.1226	1.28 (0.93-1.76)
rs10512536	68158248	C/T	0.14	0.11	0.1488	1.26 (0.92-1.74)
rs4968800	68158877	C/T	0.14	0.11	0.1458	1.26 (0.92-1.74)
rs4968886	68159501	C/T	0.14	0.11	0.1386	1.27 (0.92-1.75)
rs4968801	68159800	G/A	0.14	0.11	0.1226	1.28 (0.93-1.76)
rs76368932	68160311	T/C	0.14	0.11	0.1226	1.28 (0.93-1.76)
rs8066863	68160901	G/A	0.14	0.11	0.1226	1.28 (0.93-1.76)
rs34366352	68161384	C/T	0.14	0.11	0.1175	1.28 (0.94-1.76)
rs12452852	68161876	C/A	0.14	0.11	0.1175	1.28 (0.94-1.76)
rs9896657	68177717	A/G	0.47	0.43	0.1661	1.17 (0.94-1.45)
rs4328485	68177765	A/C	0.47	0.43	0.1949	1.15 (0.93-1.44)
rs236519	68179786	C/T	0.01	0.04	0.0260	0.37 (0.15-0.91)
rs8079702	68190826	G/A	0.39	0.41	0.4112	0.91 (0.73-1.14)
rs1514239	68196045	T/C	0.46	0.45	0.9521	1.01 (0.81-1.26)
rs12601653	68198835	A/G	0.06	0.06	0.6994	1.09 (0.70-1.71)
rs1468473	68201359	T/C	0.40	0.41	0.6430	0.95 (0.76-1.19)
rs236585	68202513	T/C	0.45	0.45	0.9783	1.00 (0.81-1.25)
rs11871843	68202722	T/C	0.02	0.02	0.6769	0.84 (0.36-1.93)
rs236587	68203915	T/C	0.45	0.45	0.9515	0.99 (0.80-1.24)
rs62069106	68206592	G/A	0.02	0.02	0.6088	0.80 (0.35-1.85)
rs236593	68207310	T/C	0.45	0.45	0.9272	1.01 (0.81-1.26)
rs236594	68207627	A/G	0.45	0.45	0.8909	1.02 (0.81-1.27)
rs8075014	68208200	G/A	0.02	0.02	0.6088	0.80 (0.35-1.85)
rs62069108	68208414	A/G	0.16	0.15	0.6706	1.07 (0.79-1.45)
rs62069109	68208690	A/G	0.16	0.15	0.6706	1.07 (0.79-1.45)
rs8076345	68212550	A/G	0.16	0.15	0.5155	1.10 (0.82-1.48)
rs236523	68212642	C/A	0.38	0.39	0.4964	0.93 (0.74-1.16)
rs236528	68215590	C/T	0.42	0.45	0.2748	0.89 (0.71-1.11)
rs638538	68216128	C/A	0.44	0.47	0.2072	0.87 (0.70-1.09)
rs236530	68217471	T/C	0.43	0.47	0.2425	0.88 (0.70-1.10)
rs236531	68217860	C/T	0.30	0.32	0.4340	0.91 (0.72-1.16)
rs236532	68219001	T/C	0.30	0.32	0.4323	0.91 (0.72-1.16)
rs236533	68219033	G/A	0.44	0.47	0.2031	0.87 (0.70-1.08)
rs236534	68219222	T/C	0.28	0.30	0.5446	0.93 (0.73-1.18)
rs600741	68220218	G/A	0.29	0.32	0.3651	0.90 (0.70-1.14)
rs669319	68220226	T/C	0.30	0.32	0.4002	0.90 (0.71-1.15)
rs79139025	68220868	G/A	0.14	0.15	0.5989	0.92 (0.67-1.26)
rs656367	68220870	C/T	0.29	0.32	0.3272	0.89 (0.70-1.13)
rs2366491	68222235	T/C	0.14	0.15	0.7388	0.95 (0.69-1.30)
rs12150382	68222772	A/G	0.15	0.18	0.2529	0.84 (0.62-1.14)
rs9909591	68224379	G/A	0.14	0.15	0.3382	0.86 (0.62-1.18)
rs9896666	68226321	T/C	0.14	0.17	0.1570	0.80 (0.59-1.10)
rs236540	68226346	G/A	0.29	0.30	0.6983	0.95 (0.75-1.22)
rs236541	68226876	T/C	0.29	0.30	0.7143	0.96 (0.75-1.22)
rs236542	68227020	C/T	0.28	0.30	0.6249	0.94 (0.74-1.20)
rs79833792	68227600	C/T	0.14	0.16	0.3175	0.85 (0.62-1.17)

© 2019 Zhao S-X et al. *JAMA Network Open.*

rs236543	68228766	C/T	0.29	0.30	0.6493	0.95 (0.74-1.21)
rs73388078	68229222	A/G	0.14	0.16	0.3053	0.85 (0.62-1.17)
rs73388081	68229302	A/G	0.14	0.16	0.3053	0.85 (0.62-1.17)
rs236544	68229691	A/G	0.28	0.29	0.6279	0.94 (0.74-1.20)
rs236545	68229709	C/T	0.29	0.30	0.7426	0.96 (0.76-1.22)
rs9908272	68229826	C/A	0.14	0.16	0.3053	0.85 (0.62-1.17)
rs236547	68229995	G/A	0.29	0.30	0.6560	0.95 (0.74-1.21)
rs9915538	68230544	G/A	0.14	0.17	0.1733	0.81 (0.59-1.11)
rs236548	68231252	A/G	0.43	0.47	0.1650	0.86 (0.69-1.07)
rs2886687	68231807	C/T	0.14	0.15	0.3629	0.87 (0.63-1.19)
rs17703427	68232008	G/A	0.14	0.15	0.3629	0.87 (0.63-1.19)
rs145338408	68233558	C/T	0.14	0.15	0.3720	0.87 (0.63-1.19)
rs236596	68233954	G/A	0.27	0.29	0.5273	0.92 (0.72-1.18)
rs10512538	68237670	C/T	0.01	0.02	0.2393	0.59 (0.24-1.45)
rs59180775	68241227	A/G	0.14	0.15	0.3723	0.87 (0.63-1.19)
rs999978	68242091	T/C	0.13	0.15	0.3054	0.85 (0.62-1.17)
rs79929224	68244064	A/C	0.14	0.15	0.3956	0.87 (0.64-1.20)
rs9903628	68257788	A/G	0.26	0.32	0.0127	0.73 (0.57-0.94)
rs623011	68259446	A/G	0.63	0.47	1.19×10^{-8}	1.92 (1.53-2.41)
rs189428509	68261928	A/G	0.02	0.03	0.2531	0.64 (0.30-1.38)
rs4968887	68264545	T/C	0.12	0.21	9.67×10^{-5}	0.53 (0.38-0.73)
rs236499	68266506	G/A	0.34	0.49	2.99×10^{-8}	0.53 (0.42-0.66)
rs236501	68267059	A/G	0.34	0.49	3.22×10^{-8}	0.53 (0.42-0.67)
rs12453707	68267496	T/C	0.10	0.17	0.0007	0.55 (0.39-0.78)
rs7221161	68267720	T/G	0.34	0.49	4.78×10^{-8}	0.53 (0.42-0.67)
rs12453584	68267733	A/G	0.10	0.17	0.0007	0.55 (0.39-0.78)
rs236504	68268736	C/T	0.22	0.29	0.0048	0.69 (0.53-0.90)
rs8079210	68269140	A/C	0.34	0.49	2.00×10^{-8}	0.52 (0.42-0.66)
rs7223705	68269712	A/C	0.34	0.49	1.71×10^{-8}	0.52 (0.41-0.66)
rs180505	68269838	G/A	0.34	0.49	1.71×10^{-8}	0.52 (0.41-0.66)
rs9894659	68270649	A/G	0.22	0.28	0.0057	0.70 (0.53-0.91)
rs1399185	68270955	T/G	0.34	0.49	1.92×10^{-8}	0.52 (0.42-0.66)
rs4389177	68271193	T/C	0.34	0.49	1.92×10^{-8}	0.52 (0.42-0.66)
rs4583288	68271196	A/G	0.22	0.29	0.0048	0.69 (0.53-0.90)
rs1399184	68271273	T/G	0.34	0.49	1.92×10^{-8}	0.52 (0.42-0.66)
rs8075271	68272060	A/G	0.12	0.21	8.65×10^{-5}	0.52 (0.38-0.73)
rs8076950	68272078	C/A	0.12	0.21	8.65×10^{-5}	0.52 (0.38-0.73)
rs17714860	68272354	G/A	0.88	0.79	8.65×10^{-5}	1.91 (1.37-2.66)
rs62070060	68272544	G/A	0.12	0.21	9.00×10^{-5}	0.52 (0.38-0.73)
rs9892944	68273720	A/G	0.64	0.46	1.40×10^{-10}	2.07 (1.65-2.60)
rs11650230	68273753	T/C	0.12	0.21	9.32×10^{-5}	0.52 (0.38-0.73)
rs180506	68274205	G/A	0.64	0.46	8.42×10^{-11}	2.10 (1.67-2.64)
rs723498	68275532	A/C	0.24	0.33	0.0005	0.64 (0.50-0.83)
rs723499	68275605	C/T	0.64	0.46	1.38×10^{-10}	2.07 (1.65-2.60)
rs723500	68275623	T/C	0.64	0.46	1.38×10^{-10}	2.07 (1.65-2.60)
rs8074548	68275724	T/C	0.10	0.18	0.0005	0.54 (0.38-0.77)
rs723501	68275847	A/G	0.64	0.46	1.38×10^{-10}	2.07 (1.65-2.60)
rs7222891	68276108	A/G	0.64	0.46	1.38×10^{-10}	2.07 (1.65-2.60)
rs7221253	68276217	G/A	0.64	0.46	1.38×10^{-10}	2.07 (1.65-2.60)
rs28617256	68276235	A/G	0.24	0.33	0.0006	0.65 (0.50-0.83)
rs78676535	68276485	A/G	0.02	0.03	0.1207	0.53 (0.23-1.20)
rs4968888	68276731	A/G	0.64	0.46	1.38×10^{-10}	2.07 (1.65-2.60)

© 2019 Zhao S-X et al. *JAMA Network Open.*

rs236535	68277316	C/T	0.64	0.46	8.60×10^{-11}	2.10 (1.67-2.64)
rs6501383	68277932	T/C	0.64	0.46	1.38×10^{-10}	2.07 (1.65-2.60)
rs1828813	68278194	A/G	0.64	0.46	1.38×10^{-10}	2.07 (1.65-2.60)
rs1828814	68278250	G/A	0.64	0.47	3.24×10^{-10}	2.05 (1.63-2.57)
rs16975455	68278586	T/C	0.10	0.18	0.0005	0.54 (0.38-0.77)
rs79347292	68278886	T/G	0.02	0.03	0.1207	0.53 (0.23-1.20)
rs7208109	68278903	G/A	0.64	0.47	2.18×10^{-10}	2.05 (1.63-2.58)
rs2215027	68279437	A/G	0.10	0.18	0.0005	0.54 (0.38-0.77)
rs12938938	68280430	A/G	0.64	0.46	1.38×10^{-10}	2.07 (1.65-2.60)
rs12939213	68280502	A/G	0.64	0.46	1.38×10^{-10}	2.07 (1.65-2.60)
rs75068344	68280737	G/A	0.02	0.03	0.1204	0.53 (0.23-1.20)
rs312709	68281054	G/A	0.64	0.46	1.38×10^{-10}	2.07 (1.65-2.60)
rs312710	68281101	C/T	0.24	0.33	0.0005	0.64 (0.50-0.83)
rs72631344	68281571	T/C	0.10	0.17	0.0007	0.55 (0.39-0.78)
rs312712	68281835	G/T	0.64	0.46	1.40×10^{-10}	2.07 (1.65-2.60)
rs312713	68282064	A/G	0.64	0.46	1.40×10^{-10}	2.07 (1.65-2.60)
rs76244652	68282566	T/C	0.02	0.03	0.1207	0.53 (0.23-1.20)
rs312715	68282845	G/A	0.64	0.46	1.40×10^{-10}	2.07 (1.65-2.60)
rs312718	68284376	C/T	0.64	0.46	1.40×10^{-10}	2.07 (1.65-2.60)
rs116189418	68285185	G/A	0.02	0.03	0.1207	0.53 (0.23-1.20)
rs312719	68285294	T/C	0.64	0.46	1.40×10^{-10}	2.07 (1.65-2.60)
rs4968890	68286268	T/C	0.10	0.17	0.0007	0.55 (0.39-0.78)
rs312721	68286361	C/T	0.64	0.46	1.40×10^{-10}	2.07 (1.65-2.60)
rs312723	68286979	T/C	0.24	0.33	0.0005	0.64 (0.50-0.83)
rs312725	68288205	T/C	0.64	0.46	1.40×10^{-10}	2.07 (1.65-2.60)
rs312726	68288328	C/A	0.64	0.46	1.45×10^{-10}	2.07 (1.65-2.60)
rs77358052	68288607	A/G	0.02	0.03	0.1207	0.53 (0.23-1.20)
rs312727	68288737	T/C	0.64	0.46	1.40×10^{-10}	2.07 (1.65-2.60)
rs312728	68289703	A/G	0.64	0.46	1.40×10^{-10}	2.07 (1.65-2.60)
rs11077488	68290082	T/C	0.10	0.17	0.0007	0.55 (0.39-0.78)
rs112493021	68290533	T/G	0.02	0.03	0.1207	0.53 (0.23-1.20)
rs4968891	68290551	A/G	0.10	0.17	0.0007	0.55 (0.39-0.78)
rs407414	68290982	G/T	0.64	0.46	1.40×10^{-10}	2.07 (1.65-2.60)
rs6501384	68291133	T/C	0.10	0.18	0.0005	0.54 (0.38-0.77)
rs411079	68291371	C/A	0.76	0.67	0.0005	1.56 (1.21-2.01)
rs432241	68291674	T/C	0.64	0.46	1.40×10^{-10}	2.07 (1.65-2.60)
rs401070	68292119	T/C	0.64	0.46	1.40×10^{-10}	2.07 (1.65-2.60)
rs393743	68292157	T/C	0.64	0.46	1.40×10^{-10}	2.07 (1.65-2.60)
rs427309	68292713	G/A	0.24	0.32	0.0007	0.65 (0.50-0.83)
rs427986	68292929	A/C	0.64	0.46	1.40×10^{-10}	2.08 (1.66-2.62)
rs683896	68293560	T/C	0.64	0.46	1.19×10^{-10}	2.09 (1.66-2.62)
rs655016	68294034	C/T	0.64	0.46	1.20×10^{-10}	2.09 (1.66-2.62)
rs7223796	68294560	G/A	0.64	0.46	1.21×10^{-10}	2.09 (1.66-2.62)
rs2573030	68294755	T/C	0.64	0.46	7.19×10^{-11}	2.10 (1.67-2.65)
rs2529682	68294976	G/A	0.64	0.46	7.19×10^{-11}	2.10 (1.67-2.65)
rs2529683	68294991	T/C	0.34	0.51	2.81×10^{-9}	0.50 (0.40-0.63)
rs6501385	68295062	T/C	0.64	0.46	6.58×10^{-11}	2.11 (1.68-2.65)
rs427373	68295085	C/T	0.64	0.46	6.58×10^{-11}	2.11 (1.68-2.65)
rs312692	68295241	T/G	0.64	0.46	6.58×10^{-11}	2.11 (1.68-2.65)
rs312698	68296575	T/C	0.64	0.46	9.99×10^{-11}	2.09 (1.66-2.63)
rs78231362	68296605	C/T	0.02	0.03	0.1077	0.52 (0.23-1.18)
rs1606655	68296614	G/T	0.12	0.21	0.0001	0.53 (0.38-0.74)

© 2019 Zhao S-X et al. *JAMA Network Open.*

rs1606656	68296785	T/C	0.13	0.21	0.0001	0.53 (0.38-0.74)
rs420572	68297360	T/C	0.64	0.46	1.07×10^{-10}	2.09 (1.66-2.63)
rs186362	68297408	C/A	0.23	0.33	0.0003	0.62 (0.48-0.81)
rs188744	68297454	G/A	0.23	0.33	0.0003	0.62 (0.48-0.81)
rs312699	68297537	T/C	0.23	0.33	0.0004	0.63 (0.49-0.82)
rs312700	68297567	T/C	0.64	0.46	1.15×10^{-10}	2.09 (1.66-2.63)
rs76386494	68297750	G/T	0.02	0.03	0.1877	0.60 (0.28-1.30)
rs60646596	68298093	C/T	0.11	0.18	0.0007	0.55 (0.39-0.79)
rs192853	68298292	G/A	0.23	0.33	0.0003	0.62 (0.48-0.81)
rs312703	68298992	C/T	0.64	0.46	1.37×10^{-10}	2.08 (1.66-2.62)
rs8078034	68299198	A/G	0.11	0.18	0.0007	0.55 (0.39-0.79)
rs312704	68299201	C/T	0.64	0.46	1.15×10^{-10}	2.09 (1.66-2.63)
rs7222503	68299785	G/T	0.89	0.82	0.0007	1.81 (1.27-2.56)
rs312707	68300636	A/C	0.23	0.33	0.0003	0.62 (0.48-0.81)
rs72631347	68302616	T/C	0.10	0.17	0.0008	0.55 (0.39-0.79)
rs78804406	68303195	C/T	0.02	0.03	0.1162	0.52 (0.23-1.19)
rs7225313	68303286	A/C	0.10	0.18	0.0006	0.55 (0.38-0.78)
rs2573032	68306092	T/C	0.65	0.47	1.96×10^{-10}	2.06 (1.64-2.59)
rs10163526	68306707	G/A	0.64	0.46	8.68×10^{-11}	2.09 (1.66-2.63)
rs312729	68306837	A/G	0.64	0.47	2.54×10^{-10}	2.05 (1.63-2.57)
rs2048677	68308227	C/T	0.64	0.46	1.29×10^{-10}	2.08 (1.65-2.61)
rs312730	68308334	T/C	0.64	0.47	1.86×10^{-10}	2.06 (1.64-2.59)
rs312731	68308709	T/G	0.64	0.47	1.96×10^{-10}	2.06 (1.64-2.59)
rs80076208	68309009	T/C	0.02	0.03	0.1074	0.52 (0.23-1.18)
rs79488343	68309158	T/C	0.02	0.03	0.0995	0.51 (0.22-1.16)
rs312732	68309320	T/G	0.64	0.47	1.96×10^{-10}	2.06 (1.64-2.59)
rs312733	68310541	T/C	0.23	0.32	0.0004	0.64 (0.49-0.82)
rs312735	68311236	C/T	0.24	0.33	0.0006	0.64 (0.50-0.83)
rs312736	68311680	T/C	0.64	0.47	3.14×10^{-10}	2.04 (1.62-2.56)
rs925650	68314145	G/A	0.10	0.17	0.0012	0.57 (0.40-0.81)
rs17715903	68314432	T/C	0.02	0.03	0.1111	0.52 (0.23-1.18)
rs312739	68315792	T/C	0.64	0.46	3.43×10^{-10}	2.04 (1.62-2.56)
rs16975551	68316459	T/C	0.10	0.17	0.0012	0.57 (0.40-0.81)
rs11653587	68316678	A/G	0.10	0.17	0.0011	0.56 (0.40-0.80)
rs312740	68316925	G/A	0.64	0.46	3.58×10^{-10}	2.04 (1.62-2.56)
rs17716015	68318051	A/G	0.02	0.03	0.1151	0.52 (0.23-1.19)
rs312742	68318486	T/C	0.24	0.33	0.0005	0.64 (0.50-0.83)
rs312743	68318637	C/T	0.64	0.46	3.17×10^{-10}	2.03 (1.62-2.55)
rs219937	68318758	T/C	0.64	0.46	2.03×10^{-10}	2.05 (1.64-2.57)
rs312744	68319100	T/C	0.24	0.33	0.0005	0.64 (0.50-0.83)
rs4459603	68319400	T/C	0.10	0.17	0.0011	0.56 (0.39-0.80)
rs72631348	68320345	G/T	0.10	0.17	0.0011	0.56 (0.40-0.80)
rs312745	68321056	G/A	0.24	0.33	0.0005	0.64 (0.50-0.83)
rs2671311	68321740	T/C	0.24	0.33	0.0006	0.65 (0.50-0.83)
rs312686	68322130	C/T	0.24	0.33	0.0005	0.64 (0.50-0.83)
rs312687	68322769	C/T	0.24	0.33	0.0006	0.64 (0.50-0.83)
rs17778418	68322828	A/G	0.12	0.20	0.0002	0.54 (0.39-0.75)
rs79197669	68322998	T/G	0.02	0.03	0.1192	0.53 (0.23-1.20)
rs16975575	68323746	G/T	0.64	0.46	7.70×10^{-11}	2.09 (1.66-2.62)
rs312688	68324335	T/C	0.64	0.46	1.64×10^{-10}	2.06 (1.64-2.58)
rs312689	68324795	G/A	0.64	0.46	1.49×10^{-10}	2.06 (1.64-2.59)
rs10852724	68325744	C/T	0.12	0.20	0.0002	0.54 (0.39-0.75)

© 2019 Zhao S-X et al. *JAMA Network Open.*

rs10775360	68325868	T/C	0.12	0.20	0.0002	0.54 (0.39-0.75)
rs312691	68326338	C/T	0.64	0.46	1.49×10^{-10}	2.06 (1.64-2.59)
rs312676	68328772	C/T	0.24	0.33	0.0005	0.64 (0.50-0.82)
rs312677	68329984	C/T	0.24	0.33	0.0005	0.64 (0.50-0.82)
rs312678	68330936	C/T	0.64	0.46	2.81×10^{-10}	2.04 (1.62-2.56)
rs312679	68331080	T/C	0.24	0.33	0.0005	0.64 (0.50-0.82)
rs312680	68331405	G/A	0.24	0.33	0.0005	0.64 (0.50-0.83)
rs72631349	68332120	T/C	0.10	0.17	0.0013	0.57 (0.40-0.81)
rs56130836	68335510	G/A	0.10	0.17	0.0013	0.57 (0.40-0.81)
rs17763595	68335669	G/A	0.01	0.03	0.1605	0.53 (0.22-1.31)
rs75210094	68337657	A/G	0.01	0.03	0.1605	0.53 (0.22-1.31)
rs10512574	68337964	C/T	0.10	0.17	0.0011	0.56 (0.40-0.80)
rs727402	68338097	G/A	0.01	0.03	0.1316	0.51 (0.21-1.25)
rs76042964	68338119	T/C	0.01	0.03	0.1605	0.53 (0.22-1.31)
rs74390260	68338132	A/C	0.01	0.03	0.1605	0.53 (0.22-1.31)
rs17175927	68339334	C/T	0.01	0.03	0.1174	0.50 (0.20-1.22)
rs16975596	68341545	G/A	0.01	0.03	0.1918	0.55 (0.22-1.37)
rs312750	68343539	G/A	0.22	0.31	0.0004	0.63 (0.48-0.82)
rs11650777	68344483	G/T	0.38	0.51	1.32×10^{-5}	0.61 (0.49-0.76)
rs11658102	68345127	C/T	0.61	0.48	1.80×10^{-6}	1.72 (1.37-2.15)
rs312752	68346332	G/A	0.22	0.30	0.0008	0.64 (0.49-0.84)
rs34819678	68346412	C/A	0.37	0.50	6.48×10^{-6}	0.60 (0.47-0.75)
rs141912644	68346672	T/C	0.37	0.50	6.48×10^{-6}	0.60 (0.47-0.75)
rs34432049	68346827	C/T	0.37	0.50	6.48×10^{-6}	0.60 (0.47-0.75)
rs312756	68347453	T/C	0.22	0.31	0.0006	0.64 (0.49-0.83)
rs312759	68347915	A/G	0.22	0.31	0.0007	0.64 (0.50-0.84)
rs11658839	68348654	C/A	0.37	0.50	5.31×10^{-6}	0.59 (0.47-0.74)
rs3915641	68351258	T/C	0.22	0.31	0.0004	0.63 (0.48-0.82)
rs9911531	68351574	A/G	0.37	0.50	5.70×10^{-6}	0.59 (0.47-0.75)
rs16975652	68353088	G/T	0.15	0.19	0.0625	0.75 (0.55-1.02)
rs312774	68353267	C/T	0.15	0.19	0.0625	0.75 (0.55-1.02)
rs16975656	68353308	C/A	0.15	0.19	0.0631	0.75 (0.55-1.02)
rs141155	68353592	C/A	0.22	0.31	0.0008	0.64 (0.50-0.84)
rs7216514	68353830	T/C	0.37	0.50	5.79×10^{-6}	0.59 (0.47-0.75)
rs118013304	68354716	G/A	0.15	0.19	0.0632	0.75 (0.55-1.02)
rs9915405	68355681	G/A	0.02	0.03	0.4471	0.74 (0.34-1.60)
rs3930668	68356715	A/G	0.15	0.19	0.1028	0.78 (0.57-1.06)
rs117005762	68358199	A/G	0.61	0.48	2.50×10^{-6}	1.70 (1.36-2.12)
rs8067429	68358543	C/A	0.02	0.03	0.4602	0.75 (0.35-1.62)
rs3848450	68358571	A/C	0.22	0.30	0.0008	0.64 (0.50-0.84)
rs56234797	68359332	A/G	0.02	0.03	0.4471	0.74 (0.34-1.60)
rs3848451	68359507	C/A	0.17	0.22	0.0471	0.75 (0.56-1.00)
rs9911312	68360298	T/C	0.02	0.03	0.4464	0.74 (0.34-1.60)
rs12453350	68360361	T/G	0.15	0.19	0.0945	0.77 (0.57-1.05)
rs3848452	68361325	C/A	0.15	0.19	0.0729	0.76 (0.56-1.03)
rs34387866	68361649	T/C	0.60	0.48	5.32×10^{-6}	1.67 (1.34-2.09)
rs3848453	68362094	C/T	0.24	0.33	0.0003	0.63 (0.49-0.82)
rs3891526	68362228	G/A	0.02	0.03	0.4464	0.74 (0.34-1.60)
rs17777464	68363962	G/A	0.02	0.03	0.4464	0.74 (0.34-1.60)
rs34397323	68364642	T/C	0.60	0.48	5.03×10^{-6}	1.67 (1.34-2.09)
rs4108132	68365103	A/G	0.17	0.22	0.0332	0.73 (0.55-0.98)
rs3848454	68365456	G/A	0.02	0.03	0.4464	0.74 (0.34-1.60)

© 2019 Zhao S-X et al. *JAMA Network Open.*

rs62070139	68366487	A/G	0.22	0.30	0.0011	0.65 (0.50-0.85)
rs16975680	68368974	C/T	0.02	0.03	0.4336	0.74 (0.34-1.59)
rs55728576	68371110	A/G	0.02	0.03	0.4464	0.74 (0.34-1.60)
rs78702439	68373950	A/G	0.02	0.03	0.4456	0.74 (0.34-1.60)
rs4485410	68375946	C/T	0.61	0.47	8.26×10^{-7}	1.75 (1.40-2.19)
rs12451295	68376823	C/T	0.63	0.50	3.36×10^{-6}	1.70 (1.36-2.13)
rs112573029	68378114	A/G	0.02	0.03	0.4456	0.74 (0.34-1.60)
rs16975694	68380657	T/C	0.78	0.70	0.0012	1.53 (1.18-1.99)
rs72631351	68380886	C/A	0.15	0.18	0.1109	0.78 (0.57-1.06)
rs1972558	68381113	G/A	0.61	0.48	2.32×10^{-6}	1.70 (1.36-2.13)
rs6501388	68381555	G/A	0.37	0.50	5.65×10^{-6}	0.60 (0.47-0.75)
rs12451506	68382303	A/G	0.13	0.18	0.0437	0.72 (0.52-0.99)
rs112365958	68385169	T/C	0.02	0.02	0.4290	0.71 (0.31-1.64)
rs12601221	68408395	T/C	0.24	0.33	0.0006	0.64 (0.50-0.83)
rs9891615	68410240	G/A	0.10	0.12	0.2877	0.83 (0.58-1.18)
rs17778406	68415535	A/G	0.10	0.11	0.5283	0.89 (0.62-1.28)
rs11077494	68416552	G/T	0.23	0.31	0.0014	0.66 (0.51-0.86)
rs16975758	68419330	G/A	0.33	0.43	0.0003	0.65 (0.52-0.82)
rs1396513	68420598	C/T	0.23	0.31	0.0008	0.65 (0.50-0.84)
rs7217259	68421684	G/A	0.33	0.43	0.0005	0.66 (0.53-0.84)
rs1396517	68421803	T/C	0.23	0.31	0.0008	0.65 (0.50-0.84)
rs9916380	68422273	A/G	0.23	0.31	0.0008	0.65 (0.50-0.84)
rs17778568	68423470	C/T	0.10	0.12	0.4797	0.88 (0.61-1.26)
rs766752	68424292	G/A	0.23	0.31	0.0008	0.65 (0.50-0.84)
rs62071763	68424806	A/G	0.33	0.43	0.0005	0.66 (0.53-0.84)
rs9898685	68428858	A/G	0.10	0.12	0.4038	0.86 (0.60-1.23)
rs2366790	68430939	T/G	0.33	0.43	0.0004	0.66 (0.53-0.84)
rs1396514	68431026	C/T	0.23	0.31	0.0008	0.65 (0.50-0.84)
rs9897538	68432122	A/C	0.23	0.31	0.0008	0.65 (0.50-0.84)
rs16975792	68433725	G/A	0.67	0.57	0.0004	1.51 (1.20-1.91)
rs6501392	68434200	A/G	0.23	0.31	0.0008	0.65 (0.50-0.84)
rs16975800	68436385	G/A	0.33	0.43	0.0003	0.66 (0.52-0.83)
rs2159437	68439660	A/G	0.22	0.31	0.0004	0.63 (0.48-0.82)
rs62071764	68439864	G/A	0.33	0.43	0.0004	0.66 (0.52-0.83)
rs1120297	68441938	C/T	0.23	0.31	0.0008	0.65 (0.50-0.84)
rs10491172	68447186	T/G	0.10	0.11	0.4423	0.87 (0.60-1.25)
rs9890686	68447566	T/C	0.10	0.11	0.4423	0.87 (0.60-1.25)
rs76647912	68448669	T/C	0.10	0.11	0.4423	0.87 (0.60-1.25)
rs11658767	68451507	C/T	0.10	0.12	0.4157	0.86 (0.60-1.23)
rs12941598	68453327	A/G	0.10	0.12	0.4865	0.88 (0.62-1.26)
rs71375785	68453496	T/C	0.10	0.12	0.4161	0.86 (0.60-1.24)
rs4603601	68470335	G/A	0.23	0.31	0.0027	0.68 (0.52-0.88)
rs1605750	68471073	A/G	0.77	0.69	0.0022	1.49 (1.15-1.93)
rs981106	68472639	A/G	0.24	0.32	0.0020	0.67 (0.52-0.87)
rs9913332	68473804	G/A	0.24	0.31	0.0024	0.68 (0.53-0.88)
rs9899840	68473933	T/C	0.24	0.31	0.0022	0.68 (0.52-0.88)
rs12103465	68474616	G/T	0.24	0.31	0.0024	0.68 (0.53-0.88)
rs12103757	68474833	A/G	0.24	0.31	0.0024	0.68 (0.53-0.88)
rs1605749	68475145	A/G	0.24	0.31	0.0024	0.68 (0.53-0.88)
rs4399570	68479345	A/G	0.10	0.12	0.3963	0.86 (0.60-1.22)
rs11658719	68481508	T/G	0.31	0.40	0.0020	0.69 (0.55-0.88)
rs60237700	68481901	G/A	0.31	0.40	0.0020	0.69 (0.55-0.88)

© 2019 Zhao S-X et al. *JAMA Network Open.*

rs16975820	68482625	A/G	0.31	0.40	0.0020	0.69 (0.55-0.88)
rs1013506	68483505	T/G	0.31	0.40	0.0019	0.69 (0.55-0.88)
rs7213968	68483944	C/T	0.25	0.32	0.0065	0.71 (0.55-0.91)
rs16975824	68484050	G/A	0.31	0.40	0.0020	0.69 (0.55-0.88)
rs59075614	68484199	C/T	0.31	0.40	0.0018	0.69 (0.55-0.88)
rs16975825	68484861	T/C	0.31	0.40	0.0016	0.69 (0.54-0.87)
rs16975827	68484911	T/C	0.31	0.40	0.0018	0.69 (0.55-0.88)
rs16975828	68485195	T/C	0.31	0.40	0.0020	0.69 (0.55-0.88)
rs57066942	68485521	C/A	0.31	0.40	0.0018	0.69 (0.55-0.88)
rs9302917	68485688	G/A	0.25	0.32	0.0062	0.71 (0.55-0.91)
rs57618720	68486241	G/A	0.31	0.40	0.0018	0.69 (0.55-0.88)
rs72631353	68486295	T/C	0.31	0.40	0.0018	0.69 (0.55-0.88)
rs61363360	68486334	G/A	0.31	0.40	0.0018	0.69 (0.55-0.88)
rs9896425	68486927	G/A	0.25	0.32	0.0075	0.71 (0.56-0.92)
rs12051831	68487515	T/C	0.31	0.40	0.0018	0.69 (0.55-0.88)
rs4793375	68488063	A/G	0.31	0.40	0.0018	0.69 (0.55-0.88)
rs4793266	68488089	G/A	0.31	0.40	0.0018	0.69 (0.55-0.88)
rs72631354	68488588	G/A	0.31	0.40	0.0018	0.69 (0.55-0.88)
rs59385779	68488603	T/C	0.31	0.40	0.0018	0.69 (0.55-0.88)
rs59310654	68488708	A/G	0.31	0.40	0.0018	0.69 (0.55-0.88)
rs60324625	68488765	G/A	0.31	0.40	0.0018	0.69 (0.55-0.88)
rs59556916	68488778	A/G	0.31	0.40	0.0018	0.69 (0.55-0.88)
rs12450317	68489384	A/G	0.31	0.40	0.0018	0.69 (0.55-0.88)
rs11652006	68489725	A/G	0.31	0.40	0.0018	0.69 (0.55-0.88)
rs72631356	68490514	C/T	0.31	0.40	0.0018	0.69 (0.55-0.88)
rs8080138	68490662	T/C	0.31	0.40	0.0019	0.69 (0.55-0.88)
rs8080507	68491130	A/G	0.31	0.40	0.0018	0.69 (0.55-0.88)
rs8066148	68491223	T/C	0.31	0.40	0.0018	0.69 (0.55-0.88)
rs62069780	68491289	G/A	0.31	0.40	0.0018	0.69 (0.55-0.88)
rs62069781	68491329	T/C	0.31	0.40	0.0018	0.69 (0.55-0.88)
rs72631358	68491516	C/A	0.31	0.40	0.0018	0.69 (0.55-0.88)
rs72631359	68491765	C/T	0.31	0.40	0.0018	0.69 (0.55-0.88)
rs62069782	68491982	A/G	0.31	0.40	0.0019	0.69 (0.55-0.88)
rs9897071	68492083	A/G	0.25	0.32	0.0074	0.71 (0.56-0.92)
rs62069783	68492256	C/T	0.69	0.60	0.0016	1.45 (1.15-1.84)
rs62069784	68492272	A/G	0.31	0.40	0.0018	0.69 (0.55-0.88)
rs62069785	68492352	A/G	0.31	0.40	0.0018	0.69 (0.55-0.88)
rs8072275	68492918	A/G	0.25	0.32	0.0064	0.71 (0.55-0.91)
rs10512539	68494308	T/C	0.31	0.40	0.0018	0.69 (0.55-0.88)
rs17779747	68494992	T/G	0.06	0.07	0.3503	0.80 (0.51-1.27)
rs62069786	68495335	T/C	0.31	0.40	0.0018	0.69 (0.55-0.88)
rs9899335	68495920	C/A	0.25	0.32	0.0063	0.71 (0.55-0.91)
rs16975850	68496503	C/T	0.31	0.40	0.0018	0.69 (0.55-0.88)
rs34249873	68496999	C/T	0.31	0.40	0.0018	0.69 (0.55-0.88)
rs72631360	68497005	C/T	0.31	0.40	0.0024	0.70 (0.55-0.88)
rs12451006	68497656	A/G	0.31	0.40	0.0018	0.69 (0.55-0.88)
rs980248	68498025	A/G	0.31	0.40	0.0019	0.69 (0.55-0.88)
rs12453924	68499065	T/G	0.31	0.40	0.0017	0.69 (0.55-0.87)
rs7210840	68500929	A/G	0.31	0.40	0.0017	0.69 (0.55-0.87)
rs11655221	68504007	A/G	0.31	0.40	0.0017	0.69 (0.55-0.87)
rs1860452	68505159	T/C	0.06	0.08	0.3254	0.79 (0.50-1.25)
rs6501398	68506237	G/A	0.31	0.40	0.0017	0.69 (0.55-0.87)

© 2019 Zhao S-X et al. *JAMA Network Open.*

rs7218368	68506682	A/G	0.06	0.08	0.3183	0.79 (0.50-1.25)
rs1594477	68507436	T/C	0.25	0.32	0.0072	0.71 (0.56-0.92)
rs173269	68508992	A/G	0.25	0.32	0.0072	0.71 (0.56-0.92)
rs7216543	68509129	G/T	0.31	0.40	0.0016	0.69 (0.54-0.87)
rs1594476	68509474	G/A	0.31	0.40	0.0016	0.69 (0.54-0.87)
rs1594475	68509649	C/T	0.32	0.40	0.0025	0.70 (0.55-0.89)
rs4793394	68509868	G/A	0.25	0.32	0.0054	0.70 (0.55-0.91)
rs4793395	68509948	T/C	0.25	0.32	0.0054	0.70 (0.55-0.91)
rs9894256	68511145	G/A	0.25	0.32	0.0055	0.70 (0.55-0.91)
rs11077499	68511177	G/A	0.31	0.40	0.0016	0.69 (0.55-0.87)
rs8070940	68512290	G/A	0.31	0.40	0.0014	0.68 (0.54-0.87)
rs62069815	68512665	T/C	0.31	0.40	0.0018	0.69 (0.55-0.88)
rs12103678	68513822	G/A	0.06	0.08	0.2535	0.76 (0.48-1.21)
rs8073016	68516092	G/T	0.32	0.39	0.0065	0.72 (0.57-0.92)
rs11650835	68532795	T/C	0.06	0.09	0.0381	0.62 (0.39-0.98)
rs350604	68532894	T/G	0.18	0.19	0.5537	0.92 (0.69-1.22)
rs11657696	68532901	C/T	0.06	0.09	0.0381	0.62 (0.39-0.98)
rs11651554	68533019	A/G	0.06	0.09	0.0555	0.65 (0.41-1.02)
rs350603	68533650	T/G	0.24	0.28	0.0800	0.80 (0.62-1.03)
rs72855908	68533703	T/C	0.06	0.09	0.0639	0.65 (0.41-1.03)
rs11653245	68534127	T/C	0.06	0.09	0.0375	0.62 (0.39-0.98)
rs11653355	68534353	T/C	0.06	0.09	0.0384	0.62 (0.39-0.98)
rs11655558	68535985	A/G	0.05	0.08	0.0700	0.64 (0.39-1.04)
rs11655611	68536105	A/G	0.05	0.08	0.0700	0.64 (0.39-1.04)
rs1825672	68536144	A/G	0.05	0.08	0.0700	0.64 (0.39-1.04)
rs7220610	68536855	C/T	0.05	0.08	0.0706	0.64 (0.39-1.04)
rs16913	68537347	T/C	0.06	0.08	0.0972	0.67 (0.42-1.08)
rs72855914	68538107	T/C	0.06	0.08	0.0972	0.67 (0.42-1.08)
rs420762	68538121	A/G	0.23	0.27	0.1490	0.83 (0.64-1.07)
rs72855918	68538413	A/G	0.06	0.08	0.0972	0.67 (0.42-1.08)
rs2630640	68538485	A/C	0.23	0.27	0.1595	0.83 (0.64-1.08)
rs34793380	68538857	T/C	0.06	0.08	0.0972	0.67 (0.42-1.08)
rs1817630	68539112	A/G	0.06	0.08	0.1147	0.68 (0.43-1.10)
rs7218450	68539320	C/A	0.06	0.08	0.0972	0.67 (0.42-1.08)
rs16975882	68540264	C/T	0.06	0.08	0.1363	0.70 (0.44-1.12)
rs72855927	68540281	G/T	0.06	0.08	0.1363	0.70 (0.44-1.12)
rs10512540	68540752	T/C	0.06	0.08	0.1400	0.71 (0.44-1.13)
rs72855936	68547220	A/G	0.06	0.08	0.1363	0.70 (0.44-1.12)
rs8080393	68548577	C/T	0.06	0.08	0.1363	0.70 (0.44-1.12)
rs8075249	68548806	A/G	0.06	0.08	0.1363	0.70 (0.44-1.12)
rs8080759	68548808	G/T	0.06	0.08	0.1363	0.70 (0.44-1.12)
rs350607	68549573	G/A	0.17	0.19	0.6042	0.93 (0.69-1.24)
rs8081864	68549692	G/A	0.06	0.08	0.1363	0.70 (0.44-1.12)
rs8066762	68549840	A/C	0.06	0.08	0.1363	0.70 (0.44-1.12)
rs55936715	68550541	T/C	0.06	0.08	0.1363	0.70 (0.44-1.12)
rs4793451	68553696	T/C	0.23	0.27	0.1754	0.84 (0.65-1.08)
rs350613	68554203	G/A	0.29	0.32	0.2198	0.86 (0.67-1.10)
rs350614	68554841	G/A	0.18	0.19	0.6286	0.93 (0.70-1.24)
rs350615	68555708	G/A	0.29	0.32	0.2119	0.86 (0.67-1.09)
rs350616	68556104	A/G	0.23	0.27	0.1695	0.84 (0.65-1.08)
rs73391629	68557427	T/G	0.06	0.08	0.1269	0.70 (0.44-1.11)
rs8081551	68558285	T/C	0.05	0.05	0.7741	1.08 (0.66-1.77)

© 2019 Zhao S-X et al. *JAMA Network Open.*

rs8073324	68560126	T/C	0.05	0.05	0.7741	1.08 (0.66-1.77)
rs1431455	68560681	A/G	0.06	0.08	0.1329	0.70 (0.44-1.12)
rs350618	68561417	T/C	0.18	0.19	0.6210	0.93 (0.70-1.24)
rs1991679	68561705	T/C	0.05	0.05	0.7741	1.08 (0.66-1.77)
rs73998863	68562393	A/G	0.05	0.05	0.7741	1.08 (0.66-1.77)
rs11655478	68562405	G/A	0.06	0.08	0.1394	0.71 (0.44-1.12)
rs7224554	68564543	C/T	0.06	0.08	0.1388	0.71 (0.44-1.12)
rs79369382	68564988	G/A	0.05	0.05	0.7501	1.09 (0.66-1.78)
rs7210525	68565508	C/T	0.05	0.05	0.7338	1.09 (0.67-1.79)
rs350620	68566532	A/G	0.16	0.18	0.5209	0.91 (0.68-1.22)
rs66975013	68566959	G/A	0.06	0.08	0.1017	0.67 (0.42-1.08)
rs75721533	68567449	T/C	0.05	0.05	0.7352	1.09 (0.67-1.79)
rs350623	68568374	G/A	0.18	0.19	0.6210	0.93 (0.70-1.24)
rs350625	68569775	C/A	0.18	0.19	0.6115	0.93 (0.70-1.24)
rs350626	68570105	C/T	0.18	0.19	0.6096	0.93 (0.70-1.24)
rs67365307	68571407	A/G	0.05	0.08	0.0638	0.63 (0.38-1.03)
rs61339122	68571916	G/A	0.06	0.08	0.0997	0.67 (0.42-1.08)
rs66695132	68572695	A/G	0.06	0.08	0.0997	0.67 (0.42-1.08)
rs78135447	68574241	C/T	0.08	0.07	0.2335	1.28 (0.85-1.92)
rs718950	68576399	T/C	0.06	0.08	0.1638	0.72 (0.45-1.15)
rs74964366	68576732	T/G	0.08	0.06	0.2237	1.29 (0.86-1.93)
rs72855756	68577077	T/C	0.06	0.08	0.1432	0.71 (0.44-1.13)
rs72855757	68577079	A/C	0.06	0.08	0.1432	0.71 (0.44-1.13)
rs1431454	68578940	T/C	0.21	0.26	0.0481	0.76 (0.59-1.00)
rs9302918	68580317	A/G	0.15	0.18	0.2248	0.83 (0.61-1.12)
rs9302919	68580485	G/T	0.21	0.26	0.0500	0.77 (0.59-1.00)
rs67720976	68580589	A/G	0.06	0.08	0.1375	0.70 (0.44-1.12)
rs66940395	68580926	G/A	0.06	0.08	0.1375	0.70 (0.44-1.12)
rs11652089	68581309	T/G	0.06	0.08	0.1375	0.70 (0.44-1.12)
rs12938026	68582006	C/T	0.15	0.18	0.2429	0.84 (0.62-1.13)
rs7220885	68582827	A/G	0.15	0.18	0.2429	0.84 (0.62-1.13)
rs16975944	68585573	T/G	0.06	0.08	0.1334	0.70 (0.44-1.12)
rs9915992	68586165	T/C	0.15	0.18	0.2414	0.84 (0.62-1.13)
rs9902449	68587002	C/T	0.07	0.11	0.0098	0.57 (0.37-0.88)
rs17779190	68588005	T/G	0.05	0.08	0.0653	0.64 (0.39-1.04)
rs11650015	68588221	G/A	0.06	0.08	0.1369	0.70 (0.44-1.12)
rs2109051	68588806	A/C	0.93	0.89	0.0107	1.74 (1.13-2.69)
rs76935250	68588900	G/A	0.08	0.07	0.2497	1.27 (0.85-1.90)
rs66985256	68589254	A/C	0.05	0.08	0.0653	0.64 (0.39-1.04)
rs9905624	68590070	T/C	0.07	0.11	0.0096	0.57 (0.37-0.88)
rs12949221	68590626	C/T	0.07	0.11	0.0093	0.57 (0.37-0.88)
rs1911969	68590720	C/T	0.27	0.32	0.1053	0.82 (0.64-1.04)
rs11658215	68590996	A/G	0.07	0.11	0.0216	0.62 (0.41-0.94)
rs9914225	68591432	T/C	0.15	0.18	0.2362	0.83 (0.62-1.13)
rs9894021	68591641	C/T	0.07	0.11	0.0227	0.62 (0.41-0.94)
rs9891523	68591900	A/G	0.07	0.11	0.0452	0.65 (0.43-0.99)
rs55776997	68592069	G/A	0.08	0.06	0.2327	1.28 (0.85-1.92)
rs720876	68592328	G/A	0.15	0.18	0.2377	0.83 (0.62-1.13)
rs56122571	68593494	G/A	0.08	0.07	0.2604	1.26 (0.84-1.89)
rs7218838	68594820	A/G	0.27	0.32	0.1072	0.82 (0.64-1.05)
rs7209535	68595027	C/T	0.15	0.18	0.2377	0.83 (0.62-1.13)
rs9896809	68595316	C/T	0.07	0.11	0.0235	0.62 (0.41-0.94)

© 2019 Zhao S-X et al. *JAMA Network Open.*

rs111881537	68595444	T/C	0.06	0.08	0.1288	0.70 (0.44-1.11)
rs8073162	68596950	G/A	0.21	0.26	0.0506	0.77 (0.59-1.00)
rs8073114	68597310	T/C	0.21	0.26	0.0506	0.77 (0.59-1.00)
rs8072003	68597329	A/G	0.06	0.08	0.1426	0.71 (0.45-1.13)
rs113756630	68597642	A/G	0.06	0.08	0.1426	0.71 (0.45-1.13)
rs11656223	68597862	G/A	0.06	0.08	0.1426	0.71 (0.45-1.13)
rs6501400	68598714	A/G	0.08	0.07	0.2829	1.25 (0.83-1.87)
rs8074266	68598912	G/A	0.08	0.07	0.2829	1.25 (0.83-1.87)
rs12601471	68599543	A/G	0.08	0.07	0.2528	1.27 (0.84-1.90)
rs12603574	68599740	G/A	0.08	0.07	0.2528	1.27 (0.84-1.90)
rs11077507	68599873	A/G	0.20	0.20	0.9729	1.00 (0.76-1.31)
rs11077508	68599950	A/G	0.14	0.15	0.7201	0.94 (0.69-1.29)
rs74628097	68599971	A/G	0.08	0.07	0.2528	1.27 (0.84-1.90)
rs7224183	68600610	C/A	0.21	0.26	0.0512	0.77 (0.59-1.00)
rs66937233	68600927	C/T	0.05	0.08	0.0679	0.64 (0.39-1.04)
rs79392721	68601245	A/G	0.08	0.07	0.2604	1.26 (0.84-1.89)
rs12449913	68601428	G/A	0.14	0.15	0.7176	0.94 (0.69-1.29)
rs76700882	68601434	G/A	0.08	0.07	0.2604	1.26 (0.84-1.89)
rs7220084	68603263	T/C	0.21	0.26	0.0506	0.77 (0.59-1.00)
rs7224857	68603331	C/T	0.21	0.26	0.0506	0.77 (0.59-1.00)
rs7221545	68603776	C/A	0.21	0.25	0.0464	0.76 (0.58-1.00)
rs1979538	68604240	G/A	0.21	0.26	0.0520	0.77 (0.59-1.00)
rs75508227	68604404	A/G	0.08	0.07	0.2528	1.27 (0.84-1.90)
rs67286965	68604814	G/A	0.06	0.08	0.1414	0.71 (0.44-1.13)
rs12949351	68605285	G/A	0.07	0.11	0.0243	0.62 (0.41-0.95)
rs78123369	68605963	G/T	0.08	0.07	0.2528	1.27 (0.84-1.90)
rs9913650	68606308	C/T	0.07	0.11	0.0204	0.61 (0.40-0.93)
rs1860316	68606316	A/G	0.28	0.32	0.1109	0.82 (0.64-1.05)
rs9914115	68606491	C/T	0.15	0.18	0.2234	0.83 (0.61-1.12)
rs9908443	68606605	C/A	0.07	0.11	0.0202	0.61 (0.40-0.93)
rs8079029	68606890	G/A	0.28	0.32	0.1142	0.82 (0.64-1.05)
rs9895773	68608047	A/G	0.14	0.15	0.8000	0.96 (0.70-1.31)
rs41459950	68609232	C/T	0.06	0.06	0.6685	1.10 (0.70-1.73)
rs12940023	68610278	C/T	0.07	0.11	0.0226	0.62 (0.41-0.94)
rs4019476	68610482	A/G	0.28	0.32	0.1305	0.83 (0.65-1.06)
rs78028239	68610605	G/A	0.08	0.07	0.2574	1.26 (0.84-1.89)
rs7211934	68610913	C/T	0.21	0.26	0.0625	0.78 (0.60-1.01)
rs4793496	68611747	C/T	0.15	0.18	0.2425	0.84 (0.62-1.13)
rs41528454	68612087	T/C	0.06	0.06	0.6173	1.12 (0.72-1.76)
rs41381246	68612119	G/A	0.14	0.15	0.8000	0.96 (0.70-1.31)
rs9908077	68613494	A/G	0.14	0.15	0.8000	0.96 (0.70-1.31)
rs7209850	68614398	C/T	0.21	0.26	0.0632	0.78 (0.60-1.02)
rs2035581	68615171	G/A	0.06	0.08	0.1751	0.73 (0.46-1.15)
rs16975961	68615246	G/A	0.14	0.15	0.7937	0.96 (0.70-1.31)
rs9904090	68616596	T/G	0.14	0.15	0.7875	0.96 (0.70-1.31)
rs16975968	68616709	A/G	0.06	0.08	0.1721	0.73 (0.46-1.15)
rs56096308	68616910	T/C	0.08	0.07	0.2594	1.26 (0.84-1.89)
rs9911708	68617532	A/G	0.14	0.15	0.7771	0.96 (0.70-1.31)
rs9907685	68618615	T/C	0.08	0.07	0.3126	1.23 (0.82-1.85)
rs16975970	68620316	A/G	0.06	0.06	0.5786	1.14 (0.72-1.78)
rs1981646	68620400	C/T	0.22	0.26	0.0693	0.78 (0.60-1.02)
rs11656723	68620561	C/T	0.06	0.08	0.1758	0.73 (0.46-1.15)

© 2019 Zhao S-X et al. *JAMA Network Open.*

rs1981647	68621193	C/T	0.21	0.24	0.1140	0.81 (0.61-1.06)
rs16975979	68621437	G/T	0.06	0.08	0.1726	0.73 (0.46-1.15)
rs9909661	68622636	A/G	0.15	0.18	0.2450	0.84 (0.62-1.13)
rs9890554	68623236	T/C	0.22	0.26	0.0654	0.78 (0.60-1.02)
rs72861479	68629947	A/C	0.06	0.08	0.0967	0.67 (0.42-1.08)
rs55792555	68633143	A/G	0.06	0.08	0.2499	0.77 (0.49-1.20)
rs16975985	68634065	G/A	0.06	0.08	0.1906	0.74 (0.47-1.17)
rs2109053	68635359	T/C	0.06	0.08	0.0785	0.66 (0.41-1.06)
rs79616191	68635756	C/A	0.05	0.05	0.9530	1.02 (0.63-1.64)
rs2240749	68636318	G/A	0.12	0.14	0.2663	0.83 (0.59-1.16)
rs16975998	68636365	A/C	0.12	0.14	0.2663	0.83 (0.59-1.16)
rs16976000	68636583	G/A	0.05	0.05	0.9530	1.02 (0.63-1.64)
rs16976002	68636917	G/T	0.05	0.05	0.9676	1.01 (0.62-1.63)
rs1843622	68637507	A/G	0.27	0.32	0.0814	0.81 (0.63-1.03)
rs11656782	68637662	A/G	0.12	0.14	0.3391	0.85 (0.61-1.19)
rs4584866	68638264	A/C	0.12	0.14	0.3289	0.85 (0.61-1.18)
rs16976008	68638688	G/A	0.12	0.14	0.3216	0.85 (0.61-1.18)
rs16976009	68638765	A/G	0.06	0.05	0.9127	1.03 (0.63-1.66)
rs16976011	68638916	G/T	0.12	0.14	0.3257	0.85 (0.61-1.18)
rs11652209	68639314	A/G	0.07	0.09	0.2012	0.76 (0.49-1.17)
rs16976024	68641368	G/A	0.12	0.14	0.2671	0.83 (0.59-1.16)
rs9891997	68641403	A/G	0.15	0.18	0.1991	0.82 (0.60-1.11)
rs16976031	68642461	A/G	0.12	0.14	0.2713	0.83 (0.59-1.16)
rs55829401	68643240	A/C	0.06	0.08	0.1487	0.72 (0.46-1.13)
rs16976038	68646652	A/G	0.11	0.14	0.1713	0.79 (0.56-1.11)
rs2191113	68647821	A/G	0.27	0.32	0.0754	0.80 (0.63-1.03)
rs8072436	68648042	A/G	0.11	0.14	0.1545	0.78 (0.55-1.10)
rs2215270	68648169	A/G	0.11	0.14	0.1843	0.79 (0.56-1.12)
rs11657599	68648697	A/C	0.06	0.08	0.1200	0.70 (0.44-1.11)
rs11654012	68648897	G/A	0.06	0.08	0.1195	0.70 (0.44-1.11)
rs58459968	68656064	A/G	0.16	0.16	0.8680	1.03 (0.76-1.38)
rs9890889	68661880	C/A	0.49	0.44	0.0631	1.24 (0.99-1.54)
rs9889243	68665634	G/A	0.28	0.29	0.6747	0.95 (0.74-1.21)
rs2367005	68666455	G/T	0.28	0.29	0.6359	0.94 (0.74-1.20)
rs17792069	68666983	C/T	0.08	0.08	0.7862	1.06 (0.71-1.57)
rs2159313	68670002	A/G	0.06	0.06	0.6298	1.12 (0.71-1.75)
rs17792098	68670039	G/A	0.18	0.19	0.5287	0.91 (0.69-1.21)
rs2109054	68670273	A/C	0.16	0.17	0.7813	0.96 (0.71-1.29)
rs7213431	68672150	G/A	0.17	0.17	0.9015	0.98 (0.73-1.32)
rs17792120	68672312	A/G	0.08	0.08	0.8866	1.03 (0.68-1.55)
rs12150650	68674056	A/G	0.13	0.12	0.7651	1.05 (0.76-1.46)
rs11077513	68675159	C/T	0.13	0.12	0.7324	1.06 (0.76-1.47)
rs57315136	68675650	A/C	0.06	0.06	0.9267	0.98 (0.63-1.53)
rs7221340	68677116	G/A	0.14	0.13	0.5634	1.10 (0.80-1.51)
rs9302921	68678695	G/A	0.12	0.12	0.8803	1.03 (0.73-1.44)
rs7222597	68679407	T/C	0.13	0.12	0.7796	1.05 (0.75-1.46)
rs7221764	68679628	A/G	0.23	0.23	0.8343	1.03 (0.79-1.33)
rs7208235	68679633	C/T	0.13	0.12	0.7693	1.05 (0.76-1.46)
rs7223156	68679731	T/C	0.13	0.12	0.8042	1.04 (0.75-1.45)
rs9894956	68680109	G/A	0.13	0.12	0.7867	1.05 (0.75-1.45)
rs8070448	68680421	C/T	0.23	0.23	0.8597	1.02 (0.79-1.33)
rs8065888	68680439	A/C	0.13	0.12	0.7768	1.05 (0.75-1.46)

© 2019 Zhao S-X et al. *JAMA Network Open.*

rs8065892	68680453	T/C	0.13	0.12	0.7768	1.05 (0.75-1.46)
rs8069361	68681016	A/G	0.13	0.12	0.7768	1.05 (0.75-1.46)
rs8071080	68681019	G/A	0.13	0.12	0.7768	1.05 (0.75-1.46)
rs9903405	68681409	G/A	0.13	0.12	0.7768	1.05 (0.75-1.46)
rs9901640	68681713	T/G	0.13	0.12	0.7768	1.05 (0.75-1.46)
rs9904269	68681749	G/A	0.13	0.12	0.7768	1.05 (0.75-1.46)
rs111247606	68681807	A/G	0.06	0.05	0.1747	1.37 (0.86-2.18)
rs9916137	68681952	C/T	0.13	0.12	0.4993	1.12 (0.80-1.56)
rs9908007	68682106	T/G	0.13	0.12	0.7768	1.05 (0.75-1.46)
rs4493104	68682335	G/A	0.13	0.12	0.7593	1.05 (0.76-1.46)
rs1486293	68682555	C/T	0.13	0.12	0.7649	1.05 (0.76-1.46)
rs1486294	68682720	C/T	0.13	0.12	0.7847	1.05 (0.75-1.45)
rs7220283	68682767	T/C	0.13	0.12	0.7743	1.05 (0.75-1.46)
rs1486295	68682937	T/C	0.13	0.12	0.7847	1.05 (0.75-1.45)
rs7210142	68683203	C/T	0.13	0.12	0.5611	1.10 (0.79-1.53)
rs1486296	68683408	T/C	0.13	0.12	0.7847	1.05 (0.75-1.45)
rs2041265	68684282	C/A	0.23	0.23	0.7341	1.05 (0.81-1.36)
rs8073288	68684927	G/A	0.13	0.12	0.7743	1.05 (0.75-1.46)
rs11870958	68685255	A/G	0.13	0.12	0.7348	1.06 (0.76-1.48)
rs16976154	68686416	G/A	0.12	0.12	0.9284	1.02 (0.73-1.42)
rs4793500	68686549	C/A	0.13	0.16	0.1574	0.79 (0.57-1.10)
rs28653114	68687916	T/C	0.13	0.12	0.7593	1.05 (0.76-1.46)
rs8066396	68688689	G/T	0.13	0.12	0.7671	1.05 (0.76-1.46)
rs8066016	68689007	T/C	0.23	0.23	0.8251	1.03 (0.79-1.34)
rs9896299	68689090	G/A	0.13	0.12	0.7746	1.05 (0.75-1.46)
rs11868346	68689387	G/T	0.13	0.12	0.7642	1.05 (0.76-1.46)
rs6501402	68689403	T/C	0.23	0.23	0.8251	1.03 (0.79-1.34)
rs11868388	68689555	C/T	0.13	0.12	0.8193	1.04 (0.75-1.45)
rs11868390	68689587	C/T	0.13	0.12	0.7746	1.05 (0.75-1.46)
rs11652213	68689838	C/T	0.13	0.12	0.7642	1.05 (0.76-1.46)
rs7214351	68689945	T/C	0.13	0.12	0.7746	1.05 (0.75-1.46)
rs2367006	68690256	C/T	0.06	0.06	0.9887	1.00 (0.64-1.56)
rs4324177	68690653	T/C	0.06	0.07	0.3764	0.82 (0.52-1.29)
rs9899334	68692854	G/A	0.13	0.12	0.7666	1.05 (0.76-1.46)
rs72863206	68695425	A/G	0.10	0.10	0.9428	1.01 (0.71-1.45)
rs7208743	68695559	C/T	0.13	0.12	0.7896	1.05 (0.75-1.45)
rs7223502	68695586	T/C	0.13	0.12	0.7821	1.05 (0.75-1.46)
rs7209270	68696131	G/A	0.13	0.12	0.7896	1.05 (0.75-1.45)
rs9807096	68699516	C/T	0.10	0.10	0.9948	1.00 (0.70-1.43)
rs112453206	68699822	A/G	0.10	0.10	0.9732	1.01 (0.70-1.44)
rs62070574	68700098	C/T	0.06	0.06	0.9922	1.00 (0.64-1.56)
rs9914343	68700594	G/A	0.13	0.12	0.7942	1.04 (0.75-1.45)
rs2159311	68700733	A/C	0.13	0.12	0.7942	1.04 (0.75-1.45)
rs2109048	68700871	G/A	0.13	0.12	0.7942	1.04 (0.75-1.45)
rs2109049	68700994	C/T	0.13	0.12	0.8069	1.04 (0.75-1.45)
rs72863209	68702080	G/A	0.10	0.10	0.9732	1.01 (0.70-1.44)
rs28589646	68702653	A/G	0.13	0.12	0.7942	1.04 (0.75-1.45)
rs11654276	68703598	G/T	0.13	0.12	0.7942	1.04 (0.75-1.45)
rs17763873	68703689	G/A	0.06	0.05	0.2054	1.34 (0.84-2.13)
rs17176195	68704203	C/T	0.13	0.12	0.7942	1.04 (0.75-1.45)
rs7208937	68705414	A/G	0.13	0.12	0.8046	1.04 (0.75-1.45)
rs10512541	68706023	A/G	0.13	0.12	0.8046	1.04 (0.75-1.45)

© 2019 Zhao S-X et al. *JAMA Network Open.*

rs9905058	68706617	T/C	0.13	0.12	0.7968	1.04 (0.75-1.45)
rs9906397	68706827	C/A	0.13	0.12	0.7741	1.05 (0.75-1.46)
rs17763885	68707044	G/A	0.10	0.10	0.8387	1.04 (0.72-1.49)
rs139865712	68707162	T/C	0.06	0.05	0.1774	1.37 (0.86-2.17)
rs35799893	68707301	T/C	0.13	0.12	0.7642	1.05 (0.76-1.46)
rs9893967	68708402	A/G	0.13	0.12	0.7963	1.04 (0.75-1.46)
rs113887955	68709483	T/C	0.06	0.05	0.1911	1.35 (0.85-2.15)
rs7226235	68711379	C/A	0.13	0.12	0.7746	1.05 (0.75-1.46)
rs12939443	68711729	A/C	0.13	0.12	0.7717	1.05 (0.76-1.46)
rs7211118	68711999	T/C	0.13	0.12	0.7717	1.05 (0.76-1.46)
rs740674	68714096	G/A	0.12	0.12	0.8658	1.03 (0.74-1.43)
rs62070600	68714214	G/A	0.06	0.06	0.8448	0.96 (0.60-1.51)
rs78107100	68715242	C/T	0.14	0.15	0.6221	0.92 (0.67-1.27)
rs8065001	68715520	T/G	0.22	0.21	0.7338	1.05 (0.80-1.36)
rs8065129	68715575	A/G	0.22	0.22	0.7461	1.04 (0.80-1.36)
rs8065449	68715808	A/G	0.12	0.11	0.8072	1.04 (0.74-1.47)
rs72863217	68715837	G/T	0.06	0.05	0.1695	1.37 (0.86-2.19)
rs8069523	68715988	A/G	0.22	0.21	0.7373	1.05 (0.80-1.36)
rs8070698	68716005	T/C	0.12	0.11	0.7916	1.05 (0.75-1.47)
rs9907805	68716927	A/G	0.22	0.22	0.7440	1.04 (0.80-1.36)
rs113689784	68717241	T/C	0.10	0.10	0.7881	1.05 (0.73-1.51)
rs717419	68718188	C/T	0.36	0.37	0.7105	0.96 (0.76-1.20)
rs4793501	68718734	T/C	0.36	0.37	0.7018	0.96 (0.76-1.20)
rs9911558	68718967	T/C	0.14	0.15	0.6364	0.93 (0.67-1.27)
rs9912421	68719000	G/A	0.24	0.25	0.7801	0.96 (0.75-1.25)
rs8082019	68719293	C/T	0.24	0.25	0.7801	0.96 (0.75-1.25)
rs73992467	68719858	G/A	0.14	0.15	0.6411	0.93 (0.67-1.28)
rs143342961	68720220	G/A	0.14	0.15	0.6411	0.93 (0.67-1.28)
rs1024642	68722140	T/C	0.22	0.21	0.7384	1.05 (0.80-1.36)
rs76156487	68722484	C/T	0.12	0.11	0.8034	1.04 (0.74-1.47)
rs1024644	68723037	C/T	0.12	0.11	0.8346	1.04 (0.74-1.46)
rs72863225	68723531	A/G	0.10	0.10	0.7860	1.05 (0.73-1.51)
rs16976178	68723707	A/G	0.22	0.22	0.7472	1.04 (0.80-1.36)
rs929474	68724036	A/G	0.35	0.34	0.9113	1.01 (0.80-1.28)
rs7213980	68724110	C/T	0.22	0.22	0.7472	1.04 (0.80-1.36)
rs1963803	68724366	T/G	0.10	0.10	0.7946	1.05 (0.73-1.50)
rs9896415	68724374	T/C	0.12	0.12	0.8581	1.03 (0.74-1.45)
rs9903552	68724590	G/T	0.12	0.12	0.8581	1.03 (0.74-1.45)
rs9903165	68724984	T/C	0.14	0.15	0.6029	0.92 (0.67-1.26)
rs1029754	68725104	G/A	0.10	0.10	0.8033	1.05 (0.73-1.50)
rs1029755	68725134	A/G	0.24	0.25	0.7836	0.97 (0.75-1.25)
rs1029756	68725240	C/T	0.10	0.10	0.8033	1.05 (0.73-1.50)
rs72863230	68726163	A/G	0.10	0.10	0.8119	1.05 (0.73-1.50)
rs74785857	68726651	T/C	0.10	0.10	0.8119	1.05 (0.73-1.50)
rs9910995	68726686	A/G	0.14	0.15	0.5869	0.92 (0.67-1.26)
rs9889316	68726935	A/G	0.14	0.15	0.5869	0.92 (0.67-1.26)
rs9898253	68727070	C/T	0.24	0.25	0.7648	0.96 (0.74-1.24)
rs9892374	68727469	T/C	0.14	0.15	0.5813	0.91 (0.66-1.26)
rs16976201	68728148	G/A	0.06	0.07	0.5513	0.87 (0.55-1.38)
rs4141188	68728238	T/C	0.10	0.10	0.8119	1.05 (0.73-1.50)
rs4141187	68728276	T/G	0.24	0.25	0.7502	0.96 (0.74-1.24)
rs8069427	68729064	G/T	0.24	0.25	0.7502	0.96 (0.74-1.24)

© 2019 Zhao S-X et al. *JAMA Network Open.*

rs72863238	68730119	A/G	0.10	0.10	0.8119	1.05 (0.73-1.50)
rs56859431	68730321	A/C	0.10	0.10	0.8119	1.05 (0.73-1.50)
rs72863242	68730618	T/C	0.06	0.05	0.1835	1.36 (0.86-2.16)
rs16976203	68730794	C/T	0.10	0.10	0.8119	1.05 (0.73-1.50)
rs9907514	68731219	A/G	0.36	0.37	0.6955	0.96 (0.76-1.20)
rs9907787	68731335	A/G	0.12	0.12	0.8787	1.03 (0.73-1.44)
rs9916370	68731347	C/T	0.14	0.15	0.5830	0.91 (0.66-1.26)
rs16976206	68732090	C/T	0.24	0.25	0.7540	0.96 (0.74-1.24)
rs16976207	68732562	T/C	0.06	0.07	0.5318	0.87 (0.55-1.37)
rs16976209	68732672	A/G	0.10	0.10	0.8119	1.05 (0.73-1.50)
rs72869790	68733304	C/T	0.06	0.05	0.1768	1.37 (0.86-2.17)
rs11868394	68734173	A/G	0.06	0.08	0.1753	0.73 (0.46-1.16)
rs72869791	68734557	G/T	0.06	0.05	0.1702	1.37 (0.86-2.19)
rs8077765	68735106	T/C	0.24	0.25	0.7540	0.96 (0.74-1.24)
rs9891034	68736046	A/G	0.24	0.25	0.7585	0.96 (0.74-1.24)
rs72869794	68736315	C/T	0.06	0.05	0.1708	1.37 (0.86-2.18)
rs62070604	68736480	C/T	0.06	0.07	0.5422	0.87 (0.55-1.37)
rs72869795	68736575	T/C	0.06	0.05	0.1715	1.37 (0.86-2.18)
rs2058177	68737418	G/A	0.24	0.25	0.6970	0.95 (0.73-1.23)
rs146631216	68740577	T/C	0.03	0.02	0.5796	1.21 (0.62-2.33)
rs16976213	68741024	G/A	0.03	0.03	0.7318	1.12 (0.58-2.16)
rs117902866	68742058	A/C	0.03	0.03	0.7501	1.11 (0.58-2.15)
rs114484479	68743570	G/A	0.03	0.03	0.9464	1.02 (0.53-1.97)
rs34730313	68743595	A/G	0.03	0.03	0.8286	1.08 (0.56-2.07)
rs116605004	68744021	T/G	0.03	0.03	0.8286	1.08 (0.56-2.07)
rs74768968	68744620	T/C	0.03	0.03	0.8275	1.08 (0.56-2.07)
rs116723200	68745437	C/T	0.03	0.03	0.8286	1.08 (0.56-2.07)
rs8069222	68745923	G/A	0.45	0.47	0.5402	0.93 (0.75-1.16)
rs16976223	68746950	C/T	0.04	0.03	0.3615	1.31 (0.73-2.35)
rs62070608	68747425	T/G	0.01	0.02	0.2507	0.59 (0.24-1.47)
rs62070609	68747590	A/C	0.01	0.02	0.2469	0.59 (0.24-1.46)
rs78156304	68748344	C/A	0.03	0.03	0.5179	1.23 (0.66-2.31)
rs62070610	68748458	C/T	0.01	0.02	0.2411	0.59 (0.24-1.45)
rs16976224	68748462	G/A	0.03	0.03	0.5179	1.23 (0.66-2.31)
rs62070611	68748657	A/G	0.01	0.02	0.2415	0.59 (0.24-1.45)
rs11869764	68749159	G/T	0.05	0.05	0.5758	0.86 (0.51-1.45)
rs11869301	68749376	G/A	0.05	0.05	0.6533	0.89 (0.53-1.49)

SNP, single nucleotide polymorphism; BP, base position; MAF, minor allele frequency; TPP, thyrotoxic hypokalemic periodic paralysis; OR, odds ratio; CI, confidence interval.

© 2019 Zhao S-X et al. *JAMA Network Open.*

eTable 8. Logistic regression analysis for SNPs in the two regions associated with TPP in the combined population

Chr.	SNP	Chr. position	Annotated genes	OR	Single-locus test *P*	r^2 with SNP A	Conditional regression *P* values	
							other SNP, conditional on SNP A	SNP A, conditional on other SNP
4q31.3	**rs1352714**	**155243604**	***DCHS2***	**1.58**	$\mathbf{1.24 \times 10^{-8}}$	/	/	/
	rs7681004	155332330	*DCHS2*	1.68	5.56×10^{-5}	0.16	0.0202	0.0005
17q24.3	rs623011	68259446	*KCNJ2/SOX9*	1.96	1.33×10^{-22}	0.89	0.6850	0.0035
	rs17714860	68272354	*KCNJ2/SOX9*	1.74	2.31×10^{-8}	0.20	0.0750	1.39×10^{-16}
	rs411079	68291371	*KCNJ2/SOX9*	1.64	1.26×10^{-10}	0.42	0.5739	2.01×10^{-14}
	rs7222503	68299785	*KCNJ2/SOX9*	1.79	6.17×10^{-8}	0.13	0.0455	1.43×10^{-18}
	rs312729	68306837	*KCNJ2/SOX9*	2.08	8.02×10^{-29}	0.92	0.2675	0.0697
	**rs312691**	**68326338**	***KCNJ2/SOX9***	**2.02**	$\mathbf{6.08 \times 10^{-24}}$	/	/	/
	rs12451295	68376823	*KCNJ2/SOX9*	1.61	2.41×10^{-13}	0.46	0.6154	3.86×10^{-13}
	rs16975694	68380657	*KCNJ2/SOX9*	1.45	1.30×10^{-6}	0.27	0.4012	4.01×10^{-19}
	rs16975792	68433725	*KCNJ2/SOX9*	1.46	4.04×10^{-8}	0.26	0.9421	3.61×10^{-18}
	rs1605750	68471073	*KCNJ2/SOX9*	1.37	4.17×10^{-5}	0.19	0.5151	1.13×10^{-20}
	rs62069783	68492256	*KCNJ2/SOX9*	1.37	6.41×10^{-6}	0.24	0.8831	2.41×10^{-20}

SNP: single nucleotide polymorphism, OR: odds ratio for the minor allele. SNP A meaned SNP with the most significance and could model the entire association signal within each susceptibility region and was shown in bold letters.

© 2019 Zhao S-X et al. *JAMA Network Open.*

eTable 9. The Association of Each Prediction Model With TPP in Total GD Participants

Markers	wGRS	P Value	OR (95%CI)	AUC (95%CI)	Threshold	Sensitivity (%)	Specificity (%)	Group	wGRS	GD No. (%)	TPP No. (%)	OR (95%CI)	P value
3 SNPs	3.44 ± 1.34	5.71×10^{-55}	1.99 (1.83-2.17)	0.74 (0.72-0.76)	4.78	66	72	Group 1	<3.44	1162 (72)	177 (34)	Reference	
								Group 2	3.44-4.78	329 (20)	181 (35)	3.61 (2.84-4.59)	2.43×10^{-27}
								Group 3	4.78-6.12	100 (6)	115 (22)	7.55 (5.53-10.31)	1.01×10^{-44}
								Group 4	>6.12	34 (2)	49 (9)	9.46 (5.94-15.07)	1.59×10^{-28}
11 SNPs	9.60 ± 2.60	7.93×10^{-73}	1.62 (1.54-1.71)	0.80 (0.78-0.82)	12.2	82	68	Group 1	<9.60	1037 (63)	76 (15)	Reference	
								Group 2	9.60-12.20	422 (26)	187 (38)	6.05 (4.53-8.08)	1.32×10^{-39}
								Group 3	12.20-14.80	182 (11)	217 (44)	16.27 (11.99-22.07)	1.8×10^{-94}
								Group 4	>14.80	8 (0.5)	11 (2)	18.76 (7.33-48.03)	1.17×10^{-16}

wGRS, weighted genetic risk score; GD, Graves' disease; TPP, thyrotoxic hypokalemic periodic paralysis; Freq, frequency; OR, odds ratio; CI, confidence interval.

3 SNPs: rs1352714 + rs2186564 + rs312691

11 SNPs: rs13400534+rs1352714 +rs7681004+ rs61748643+rs12194555+rs541442+rs7927729+rs2186564+rs12430111+rs7222503+ rs312691

© 2019 Zhao S-X et al. *JAMA Network Open.*

**eTable 10. Primers of three TPP candidate genes and for real-time reverse transcription PCR.**		
Gene Segment	Primers Sequence (5`→3`)	Product Size (bp)
DCHS2-E0-1F	5`-ACTGAAGAGGCTCCTGGATT-3`	511
DCHS2-E0-1R	5`-GCGACGAAGCTGTAGTGGT-3`	
DCHS2-E0-2F	5`-TCACCCTTTCCGTAGATGAG-3`	502
DCHS2-E0-2R	5`-AAAAGCGGTGACGGTAGTG-3`	
DCHS2-E0-3F	5`-TTCAGCACTCAGGGCTACAC-3`	522
DCHS2-E0-3R	5`-GTCCAGAGGTCTCCACACTC-3`	
DCHS2-E0-4F	5`-CTTTGAGCAGGACGAGTACC-3`	532
DCHS2-E0-4R	5`-TTCCAAGGACAGAGAGATGC-3`	
DCHS2-E0-5F	5`-TGACTGGGAGAAGGAAGATG-3`	493
DCHS2-E0-5R	5`-GGAATCAATGGCAAAAGATG-3`	
DCHS2-E0-6F	5`-AGCATTACAAGGCCTCAGTG-3`	502
DCHS2-E0-6R	5`-TTCCTAGGCCAACTCACAAC-3`	
DCHS2-E1F	5`-TTAGTCACTGGGCTCTCTGG-3`	496
DCHS2-E1R	5`-CAGCAACAGCAGCAACTAGA-3`	
DCHS2-E2F	5`-TGCCAGAGACATGAAGTTGA-3`	513
DCHS2-E2R	5`-AAGCAATCCATGACCGAATA-3`	
DCHS2-E3F	5`-CTGTCGGTGTTCCTTGACTT-3`	505
DCHS2-E3R	5`-AGCCGTTCACACAGCTAGTC-3`	
DCHS2-E4F	5`-GGGGGAATTATCACAGAAGG-3`	500
DCHS2-E4R	5`-AACTTCAGCAAAGGAAAGAGG-3`	
DCHS2-E5F	5`-TGTCTGCTGTCCACTGTGAT-3`	497
DCHS2-E5R	5`-AGATGGAGAAGGTGTGCAAG-3`	
DCHS2-E6F	5`-TTCAGCAAAAACCCATTTGT-3`	510
DCHS2-E6R	5`-GGCCAATAAAGACCACAAGA-3`	
DCHS2-E7F	5`-TGCTAAAGAAGGCTTTGTGC-3`	507
DCHS2-E7R	5`-GAGAGAGGCCCAGAAATGTT-3`	
DCHS2-E8F	5`-CAAAGCCTAATCCAGAGCAA-3`	503
DCHS2-E8R	5`-CTGTTTGTCATCCAGGCTCT-3`	
DCHS2-E9F	5`-TGTGGCCCAGTTAACACATA-3`	505
DCHS2-E9R	5`-AAAGAGCAAGCCACAGTTTG-3`	
DCHS2-E10-1F	5`-GTGGTTGTCCCCTCTCTCTT-3`	535
DCHS2-E10-1R	5`-CTCGATAACGACTGTCAGCA-3`	
DCHS2-E10-2F	5`-GTGCGGAAGACAGAGACAGT-3`	512
DCHS2-E10-2R	5`-ATTTTGCAAGAATCCGTCCT-3`	
DCHS2-E10-3F	5`-GCCTCGCCTCTTAGGTACTC-3`	503
DCHS2-E10-3R	5`-CGACTTGAGTTTTCTCAATCCT-3`	

© 2019 Zhao S-X et al. *JAMA Network Open.*

DCHS2-E11F	5`-ATACCCGGGAAAACTGAAAC-3`	497
DCHS2-E11R	5`-TGGACACCTCGTTTTCTTGT-3`	
DCHS2-E12F	5`-GCCTGTGTCTTGTTCTGCTC-3`	499
DCHS2-E12R	5`-GCATTAACCTGAAGCACCAT-3`	
DCHS2-E13F	5`-CCATAGCAACCACCTTTCAA-3`	537
DCHS2-E13R	5`-CCCAGAAGGGGATAGTTGAT-3`	
DCHS2-E14F	5`-TTTTGACTGCAATCCATCAG-3`	499
DCHS2-E14R	5`-CCTTAAATTGGGAGGCAGAT-3`	
DCHS2-E15F	5`-AGGTGATGGACTCGTGAAAG-3`	550
DCHS2-E15R	5`-GCTATGTGCAATGCTCTCAA-3`	
DCHS2-E16-1F	5`-TCACATACATGGAAGGAATTTG-3`	507
DCHS2-E16-1R	5`-TCCTATGGGAACATTCTCCTC-3`	
DCHS2-E16-2F	5`-TAGACAGCTCAACCGGAGAC-3`	497
DCHS2-E16-2R	5`-AGTGGGGTTGTGGTCATTTA-3`	
DCHS2-E16-3F	5`-TCCCGTCTTGACAGAGAAAG-3`	497
DCHS2-E16-3R	5`-GAAGGGAAAATGGGAGAAAC-3`	
DCHS2-E17F	5`-TGTTGCTTTTCTTCCATTTTC-3`	425
DCHS2-E17R	5`-GTGTTTCTGTGTGGATGCAA-3`	
DCHS2-E18F	5`-AGCAAGAACAGCCAAAAGAG-3`	500
DCHS2-E18R	5`-GGTATGAATTGTGGCCTTTG-3`	
DCHS2-E19F	5`-GAGCCAGTGCTACCTGGTTA-3`	507
DCHS2-E19R	5`-ACTCAGTCAGCATCCTGGAA-3`	
DCHS2-E20-1F	5`-GAGGGCAGAGCCTTTATCAT-3`	510
DCHS2-E20-1R	5`-ACCAGAAGCCTCATCAGTCA-3`	
DCHS2-E20-2F	5`-AGATGCTGAAGTGGGAACAG-3`	500
DCHS2-E20-2R	5`-TTAACTGCAGCCAGATAGGG-3`	
DCHS2-E20-3F	5`-CGCCTACAAACCAGACAACT-3`	502
DCHS2-E20-3R	5`-CTGTCTTCTGCCCTGTCATT-3`	
DCHS2-E21F	5`-GGATCTGGCTCATTGAAGAC-3`	498
DCHS2-E21R	5`-TTTGAAGGGCAGGATAGACA-3`	
DCHS2-E22F	5`-AGATTTCACCACTGCACTCC-3`	493
DCHS2-E22R	5`-AAAATGTACATGGCCTGGAA-3`	
DCHS2-E23F	5`-AAACAGTCGGTTCCTCACAG-3`	507
DCHS2-E23R	5`-GCAGAGGGCTCTCTAGTTCA-3`	
DCHS2-E24F	5`-TACAGTCTGGCTCTCCGAAA-3`	523
DCHS2-E24R	5`-TTGCTTGGTTGTTGAGGATT-3`	
DCHS2-E25F	5`-AATCAAGCCAAGAAAGAGCA-3`	492
DCHS2-E25R	5`-TATCGGCGATGGAAGACTTA-3`	
DCHS2-E26F	5`-CCTTCAGCCTTTAAGCACAA-3`	502

© 2019 Zhao S-X et al. *JAMA Network Open.*

DCHS2-E26R	5`-TGGGATAATTGCCTGAACTG-3`	
DCHS2-E27-1F	5`-ACTGCCGATTAAATGGATCA-3`	496
DCHS2-E27-1R	5`-CACTGTCAGATGCCAGAATG-3`	
DCHS2-E27-2F	5`-TCGGTTATCTTGTGTTGCTTC-3`	515
DCHS2-E27-2R	5`-GGAAATAGGCAGATTTTCTGG-3`	
DCHS2-E27-3F	5`-GTCCAAGCTTCAGATGCAGA-3`	493
DCHS2-E27-3R	5`-CTTCCACTCTGCCAATCAAC-3`	
DCHS2-E27-4F	5`-GTGGTCTGGGTGGATATTGA-3`	536
DCHS2-E27-4R	5`-GTCACTCTCAAGTCCGCATC-3`	
DCHS2-E27-5F	5`-TTTCTCCTCTGAAGGAACACC-3`	502
DCHS2-E27-5R	5`-AGATAGGCAGGAGAGCTGGT-3`	
DCHS2-E27-6F	5`-GCATCCCTACAGAAAGTGCT-3`	500
DCHS2-E27-6R	5`-GGGGTGGAAAAACAAAAGAT-3`	
DCHS2-E27-7F	5`-GGTGTGCAGCTCTTTCAACT-3`	519
DCHS2-E27-7R	5`-ATTCATGACCAATGGTGAGC-3`	
KCNJ2+E0-1F	5`-CTGTTTTCCAAAGCAGAAGC-3`	499
KCNJ2+E0-1R	5`-CATCCGTGACACATCTGAAA-3`	
KCNJ2+E0-2F	5`-TTCATCAATGTGGGTGAGAA-3`	494
KCNJ2+E0-2R	5`-AAGTGGCTTTTCCGAAGATT-3`	
KCNJ2+E0-3F	5`-GCCAAAGAAGAGAAACGAGA-3`	500
KCNJ2+E0-3R	5`-TGGGGACTTCGTAAGTTTTG-3`	
KCNJ2+E0-4F	5`-CATTGACAACGCAGACTTTG-3`	496
KCNJ2+E0-4R	5`-TAACTGTTTTGGGCCTCTGA-3`	
CTD-2378E21.1-E2-1F	5`-CAATGGATGAGGATGAGAGG-3`	503
CTD-2378E21.1-E2-1R	5`-AGCTCCGAAAGAAACAGTCA-3`	
CTD-2378E21.1-E2-2F	5`-AAACATTATGAGCCGCATTC-3`	491
CTD-2378E21.1-E2-2R	5`-TCTCTTTGCTCCCATCATTC-3`	
CTD-2378E21.1-E1F	5`-AGTTGGGCTAATCAATGCTG-3`	503
CTD-2378E21.1-E1R	5`-TAATGCCTTGCTCCTTGTTC-3`	
CTD-2378E21.1-E0F	5`-GAGAACCAGGAGCTCTGACA-3`	504
CTD-2378E21.1-E0R	5`-CACCATCCAATTCTTTGAGG-3`	
KCNJ2 real-time PCR F	5`-GCAACAGGACATGTTCTCTGGAT-3`	151
KCNJ2 real-time PCR R	5`-CAGTTTTGAATGTTCGGTGAAGAC-3`	
F, forward primer; R, reverse primer; bp, base pairs.		

© 2019 Zhao S-X et al. *JAMA Network Open.*

eFigure 1 The flowchart for quality filtering in the two-stage study for genome-wide association study. In the initial genome-wide association study (GWAS) stage, DNA samples from 158 thyrotoxic periodic paralysis (TPP) cases and 803 controls were genotyped using Illumina HumanOmniZhonghua-8 BeadChips (called TPP GWAS section), and those from 17 Graves' disease (GD) cases with TPP, 1,519 GD cases without TPP, and 1,516 controls were genotyped using Illumina Human660-Quad BeadChips (called GD GWAS section). Quality filtering was separately performed on SNPs and samples before analysis to ensure robust association tests for the two above-mentioned sections.

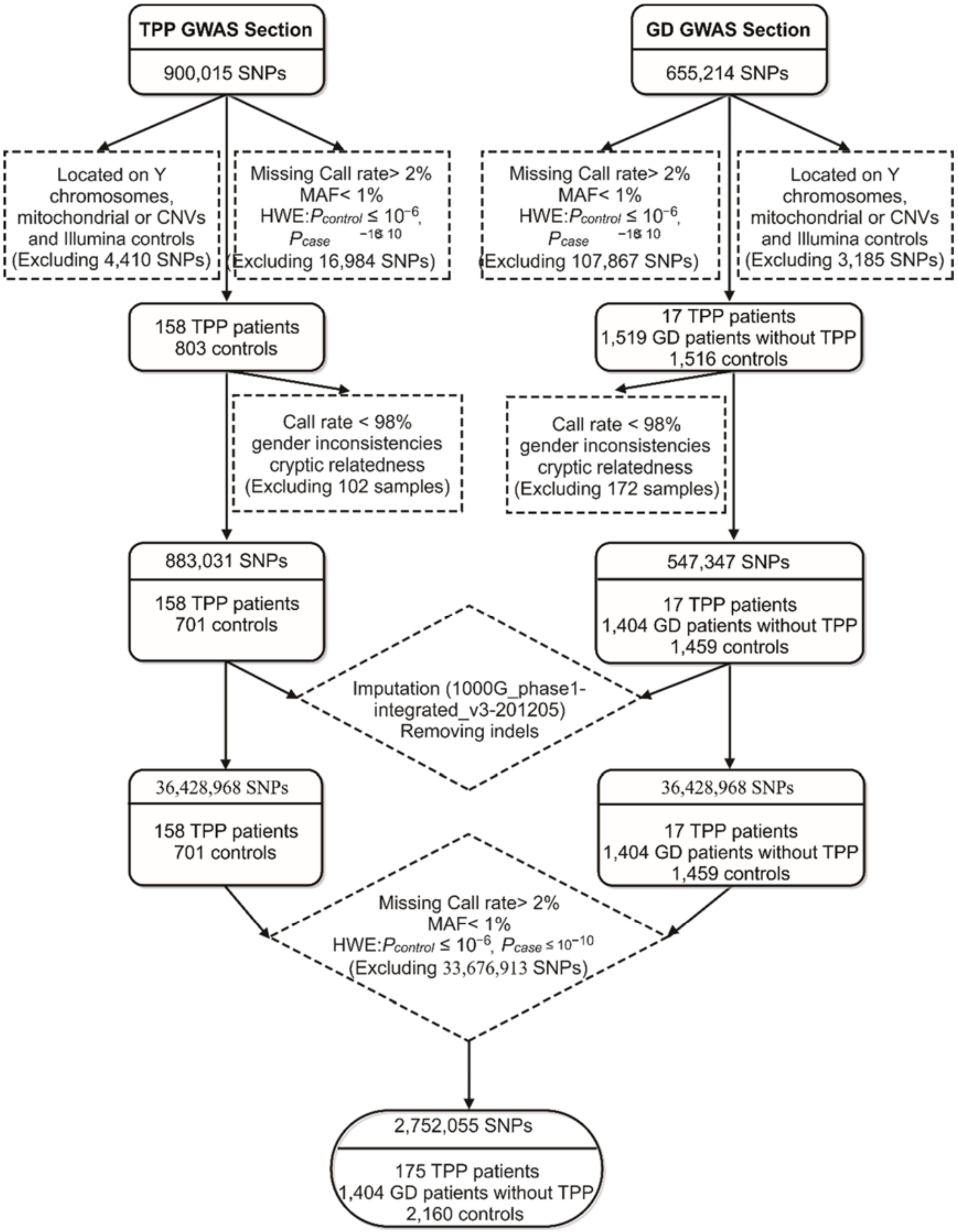

© 2019 Zhao S-X et al. *JAMA Network Open.*

eFigure 2 Summary of association results from the genome-wide scan. Genome-wide association results of TPP from the initial scan. Manhattan plot shows the genome-wide P values (-log10 P values) of the Cochran-Armitage trend test from the SNPs with discovery stage P ≤ 0.01 in 175 TPP cases and 2,160 controls (a), and in 175 TPP cases and 1,404 GD cases without TPP (b). Each chromosome is depicted as a di□erent color. The red and blue horizontal line represents P=5×10-8 and P=1×10-4.

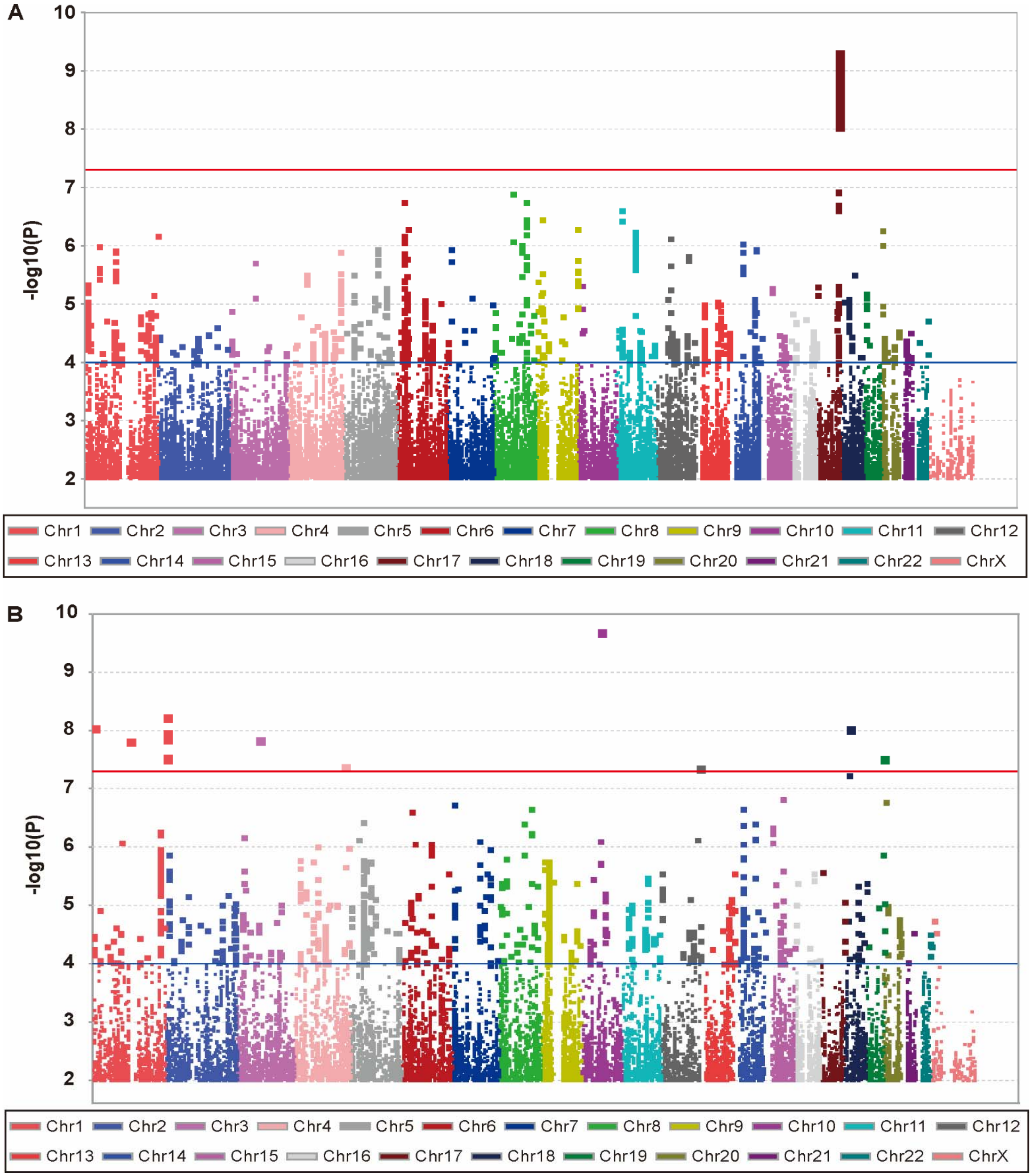

© 2019 Zhao S-X et al. *JAMA Network Open.*

eFigure 3 Plots of principal component analysis (PCA) and multidimensional scaling (MDS) analysis in our cohorts and the HapMap samples. a, Case-control cohorts (175 TPP cases and 2,160 controls) and 270 individuals from HapMap data were plotted using the first two eigenvectors produced by the EIGENSOFT software package. b, Case-control cohorts, and the 270 HapMap samples were plotted by their first and second dimension values. MDS analysis was done using the PLINK1 (http://pngu.mgh.harvard.edu/purcell/plink/) and R statistics packages (http://r-project.org). The identity-by-descent (IBD) pairwise distances among all the case-control cohorts and HapMap groups were used to construct dimensions. In both A and B, individuals who were TPP cases, controls, Chinese (CHB), Japanese (JPT), Caucasian (CEU), and Yoruban (YRI) groups were plotted in red circle, blue diamond, purple triangle point up, orange triangle point down, green cross, and black plus, respectively. The insets shown in the boxes on panel A and B were the magnification of the current cohort, CHB and JPT population, suggesting the current cohorts clearly cluster together with CHB and JPT components of the HapMap. No significant structures were observed in our current cohorts.

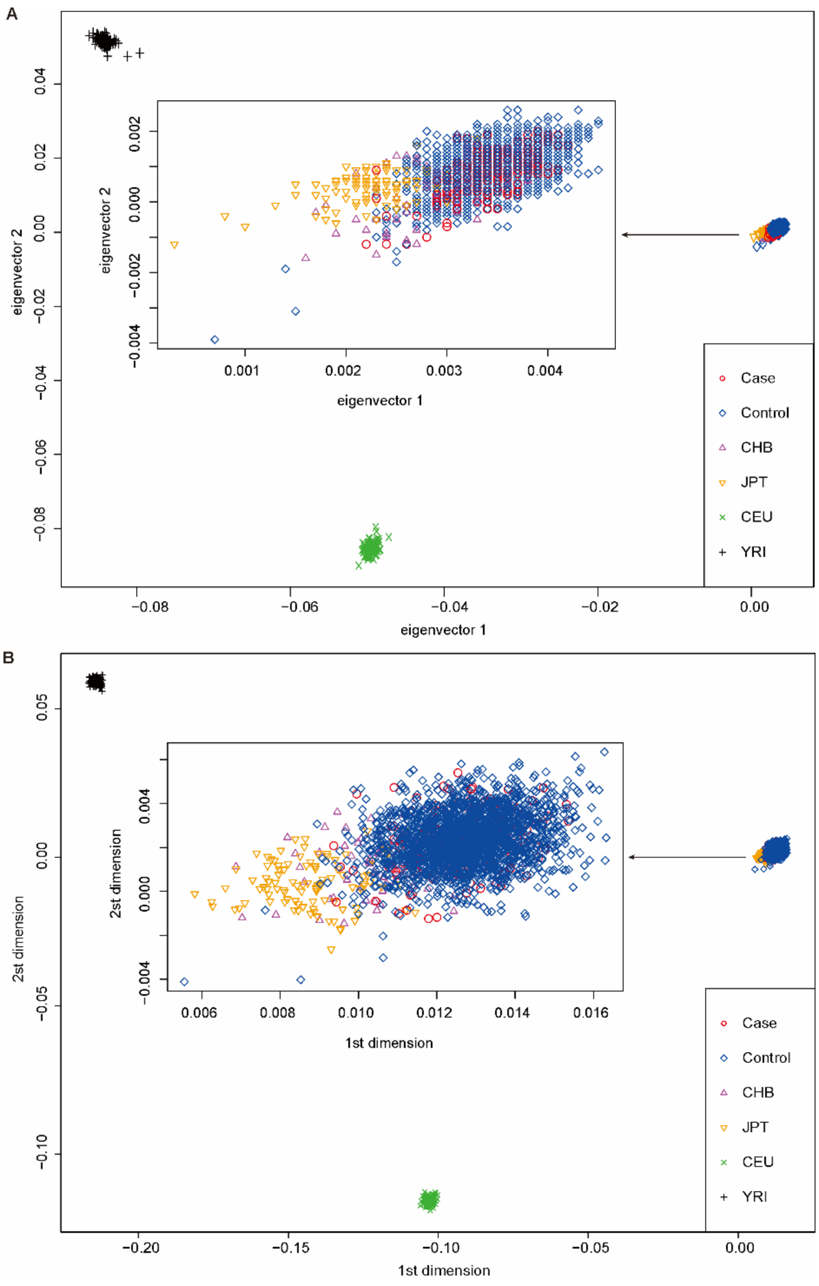

© 2019 Zhao S-X et al. *JAMA Network Open.*

eFigure 4 Quantile-quantile plot for detection of population stratification Quantile-quantile plot is shown for comparing the genome-wide distribution of the test statistic with the expected null distribution in 175 TPP cases compared to 2,160 control subjects. Since λ represents the genomic inflation factor and scales with sample sizes, we rescaled and adjusted the λ based on the samples size of 1000 cases and 1000 controls and the λ value was 0.9998.

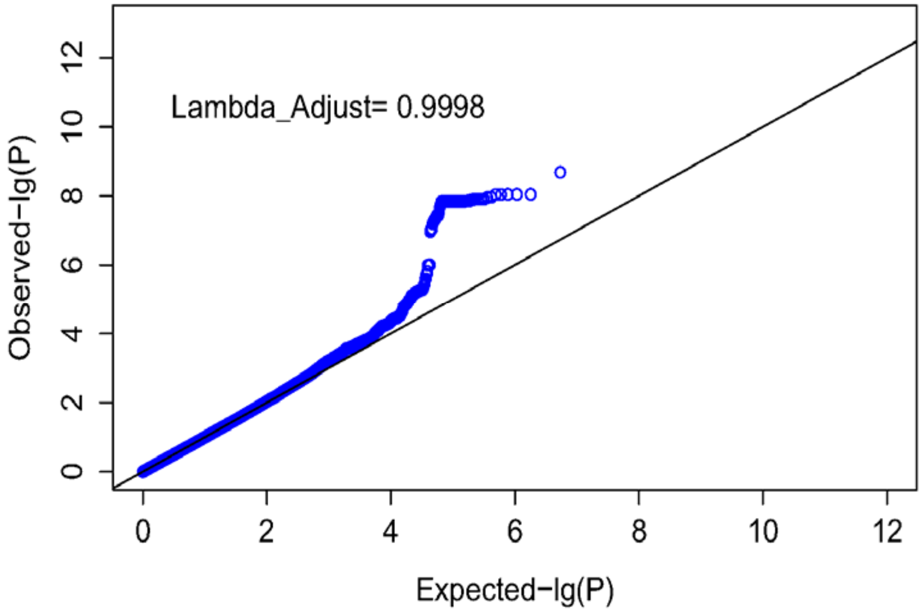

eFigure 5 The flowchart for SNPs selection of the second stage study.

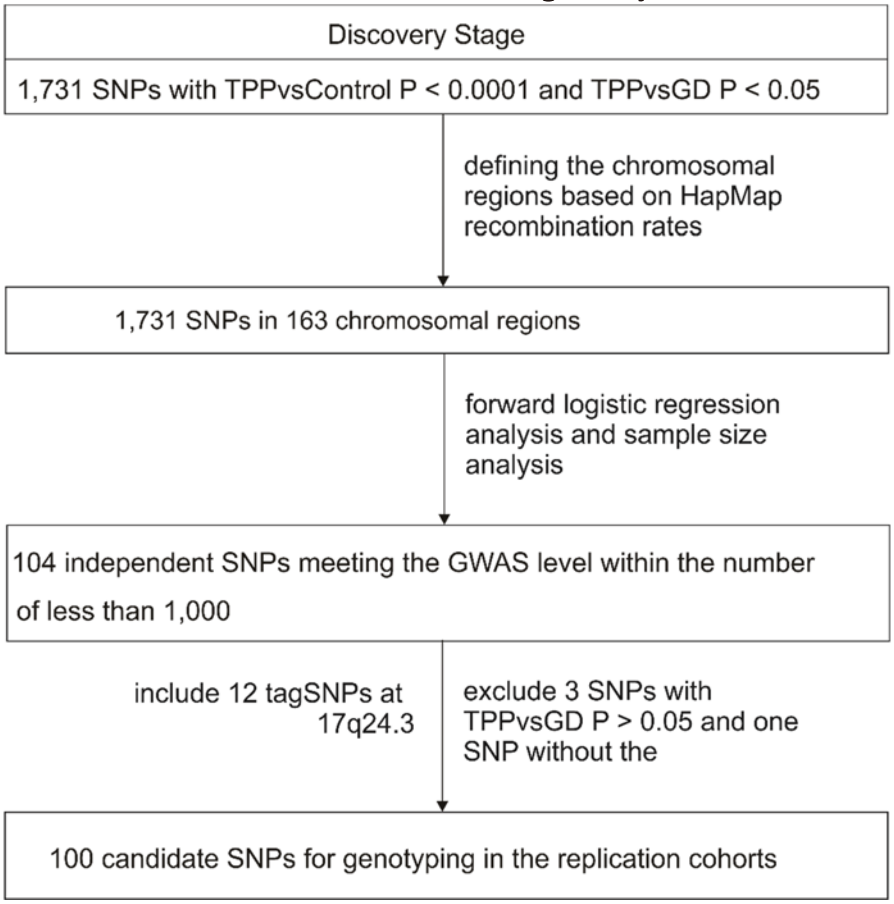

© 2019 Zhao S-X et al. *JAMA Network Open.*

eFigure 6 The logistic regression results and linkage disequilibrium block analysis at 4q31.3 (A) The two- locus conditional logistic regression results accounting for rs1352714 from the GWAS scan. The SNPs at 4q31.3 were improved by adding rs1352714 were shown in red points; whileas, the SNPs improved the model with rs1352714 were showed in blue triangles. The results showed that no other SNPs at 4q31.3 was independently associated with TPP after accounting for rs1352714. (B) The linkage disequilibrium block analysis for the 28 SNPs with discovery stage P < 0.01, which can be tagged by three replicated SNPs in the combined population by using haploview software 42.

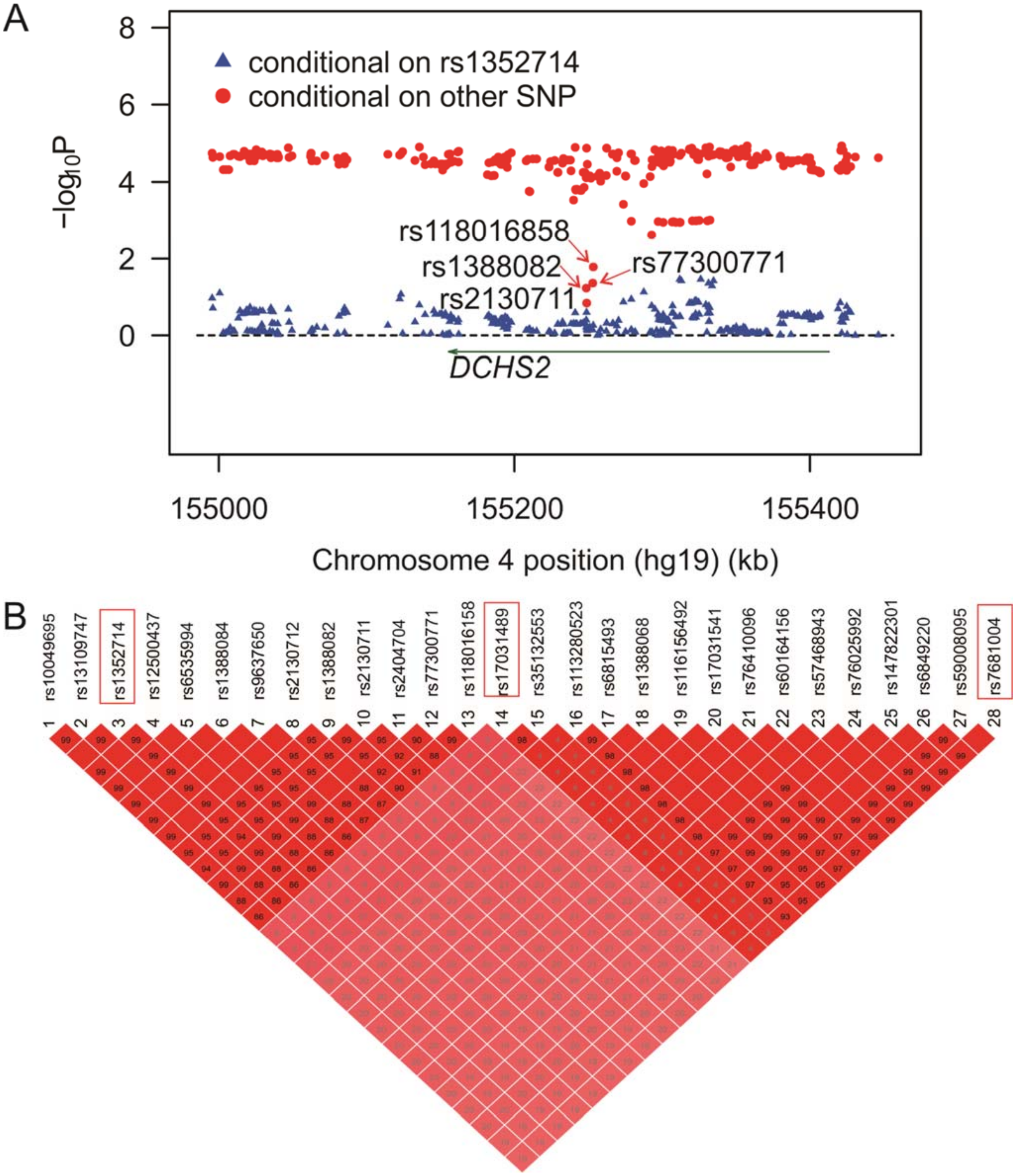

© 2019 Zhao S-X et al. *JAMA Network Open.*

eFigure 7 The cis-eQTL analyses results at 11q14.1 (A, C, E) The plots of the cis-eQTL analyses results of 128, 22, and 260 SNPs with discovery stage P <0.05 at 11q14.1 and with the significant cis-eQTLs in purified CD4+ T cells from 213 EU healthy individuals, in human naïve monocytes from 432 European volunteers, and in human skeletal muscle samples from the GTEx database. The most strongly TPP-associated signal (rs2186564) at 11q14.1 is shown in large symbols purple diamonds indicate the TPP association results. (B, D, F) The LD structures were analyzed by Haploview software version 4.2 based on the our TPP GWAS data. The LD color scheme is stratified according to the logarithm of the odds (LOD) score and D': LOD <2 (white for D'<1 and blue for D' = 1) or LOD >2 (shades of pink/red for D'<1 and bright red for D' = 1). Panel B, D, and F show the number of SNPs correlated with expression level of genes at 11q14.1 in purified CD4+ T cells, human naïve monocytes, and skeletal muscles from GTEx, is 128, 22, and 260, respectively. The most strongly TPP associated signal (rs2186564) at 11q14.1 is marked with the red rectangle. TPP = thyrotoxic periodic paralysis; eQTL = expression quantitative trait loci.

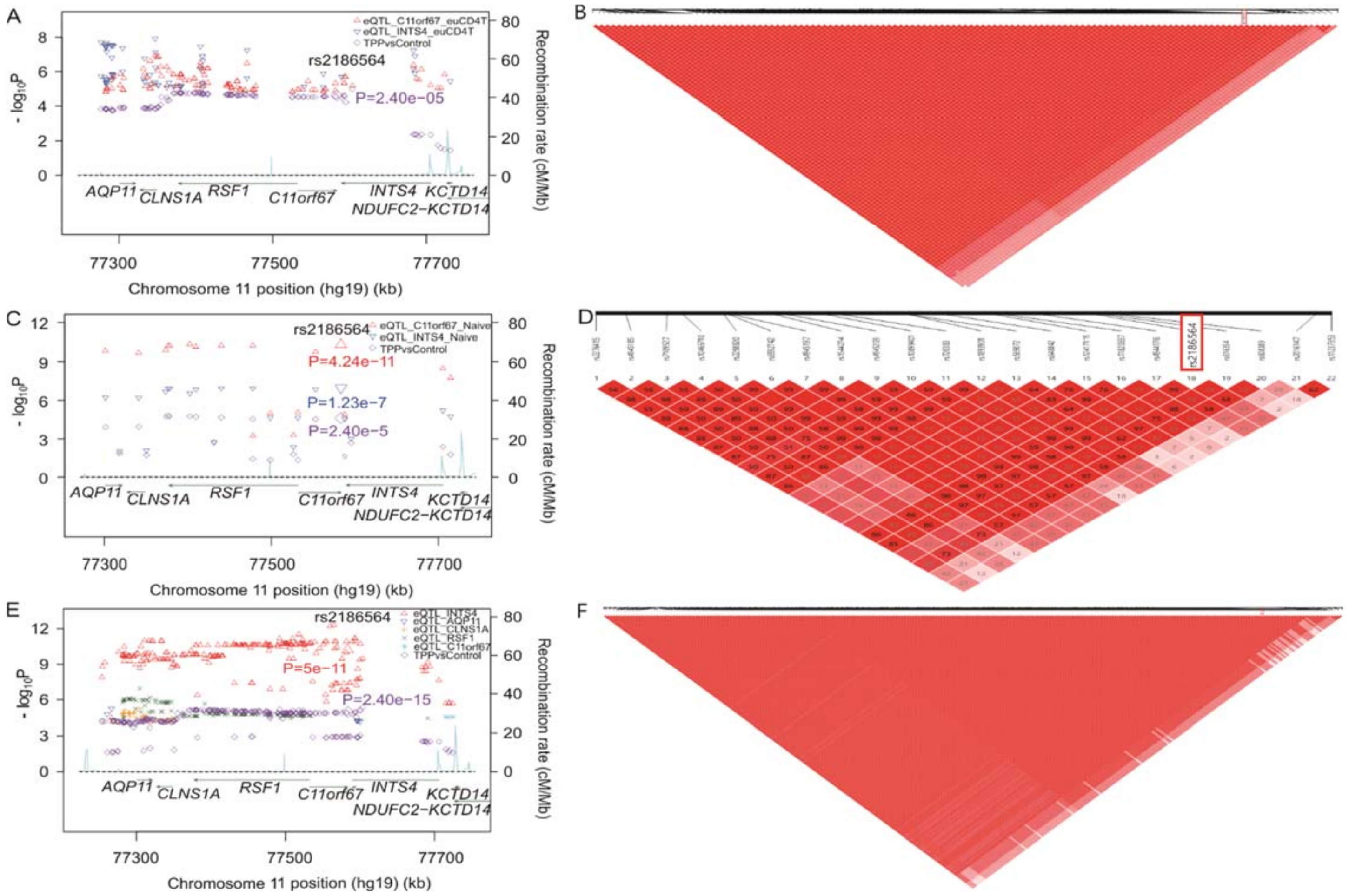

eFigure 8 The logistic regression results at 17q24.3. Panel a shows the the two-locus conditional logistic regression results at 17q24.3 accounting for rs312691 from the GWAS scan. The SNPs at 17q24.3 were improved by adding rs312691 were shown in red points; whileas, the SNPs improved the model with rs312691 were showed in blue triangles. Panel b shows the 52 SNPs highly linked with rs312691, the top SNP at 17q24.3. The linkage disequilibrium block analysis for the 52 SNPs which are highly linked with rs312691 are shown using haploview software 4.2. Conditional on each of the 52 SNPs, rs312691 (the top SNP at 17q24.3), was never associated with TPP. Panel c shows the association and stepwise logistic regression results for 12 SNPs at 17q24.3 in the combined population. Two independent SNPs (rs312691 and rs7222503) and their association P values in the combined populations are shown in red filled diamonds. The association results for 12 SNPs using

© 2019 Zhao S-X et al. *JAMA Network Open.*

the Cochran-Mantel-Hanezel (CMH) stratification analysis are shown in red triangle point up. The P values of the stepwise logistic regression of each of 11 other SNPs after accounting for rs312691 are shown in blue triangle point down, and the P values of the stepwise logistic regression of each of 10 other SNPs after accounting for rs312691 and rs7222503 are shown in black diamond.

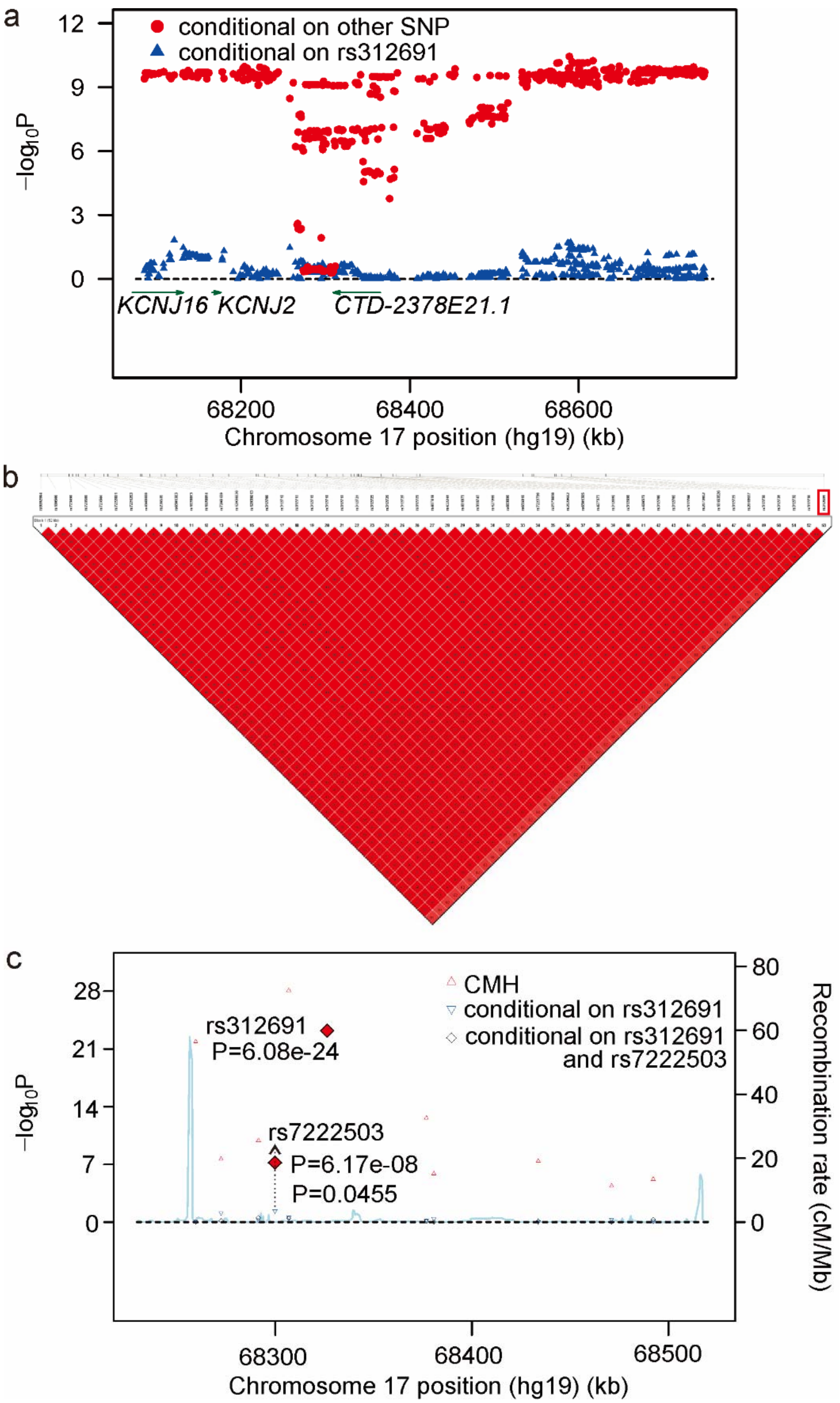

© 2019 Zhao S-X et al. *JAMA Network Open.*

eFigure 9 cis-eQTL analysis and transcript abundances of KCNJ2 at 17q24.3. Panel a shows the linkage disequilibrium (LD) structures at 17q24.3 and correlation of SNPs to transcript abundances of KCNJ2 at 17q24.3. There are 78 SNPs with P <0.05 in the initial GWAS scan of TPP and with the significant cis-eQTLs of KCNJ2 expression in the skeletal muscle from GTEx. Red triangles point up and purple diamonds indicate the correlation results of 78 SNPs to the expression level of KCNJ2 in the skeletal muscles from GTEx and the TPP association results of these 78 SNPs. And the most strongly associated signals (rs312691) are shown in large red triangle point up and large purple diamond, respectively. Panel b shows the cis-eQTL analysis of rs312691 in skeletal muscle according to GTEx single tissue eQTL database. The individuals with homogenous alternative CC genotype showed the lower expression of KCNJ2 than those with heterogenous CT genotype and those with homogenous reference TT genotypes in skeletal muscle tissues (P =1.19×10-5, β=-0.23). Panel c shows the relative mRNA expression levels of KCNJ2 in the subset of PBMCs (N=5).Panel d shows the relative mRNA expression levels of KCNJ2 for di□erent genotypes of rs312691 in PBMCs from 188 individuals (TT, N = 45; TC, N =102; and CC, N =41).

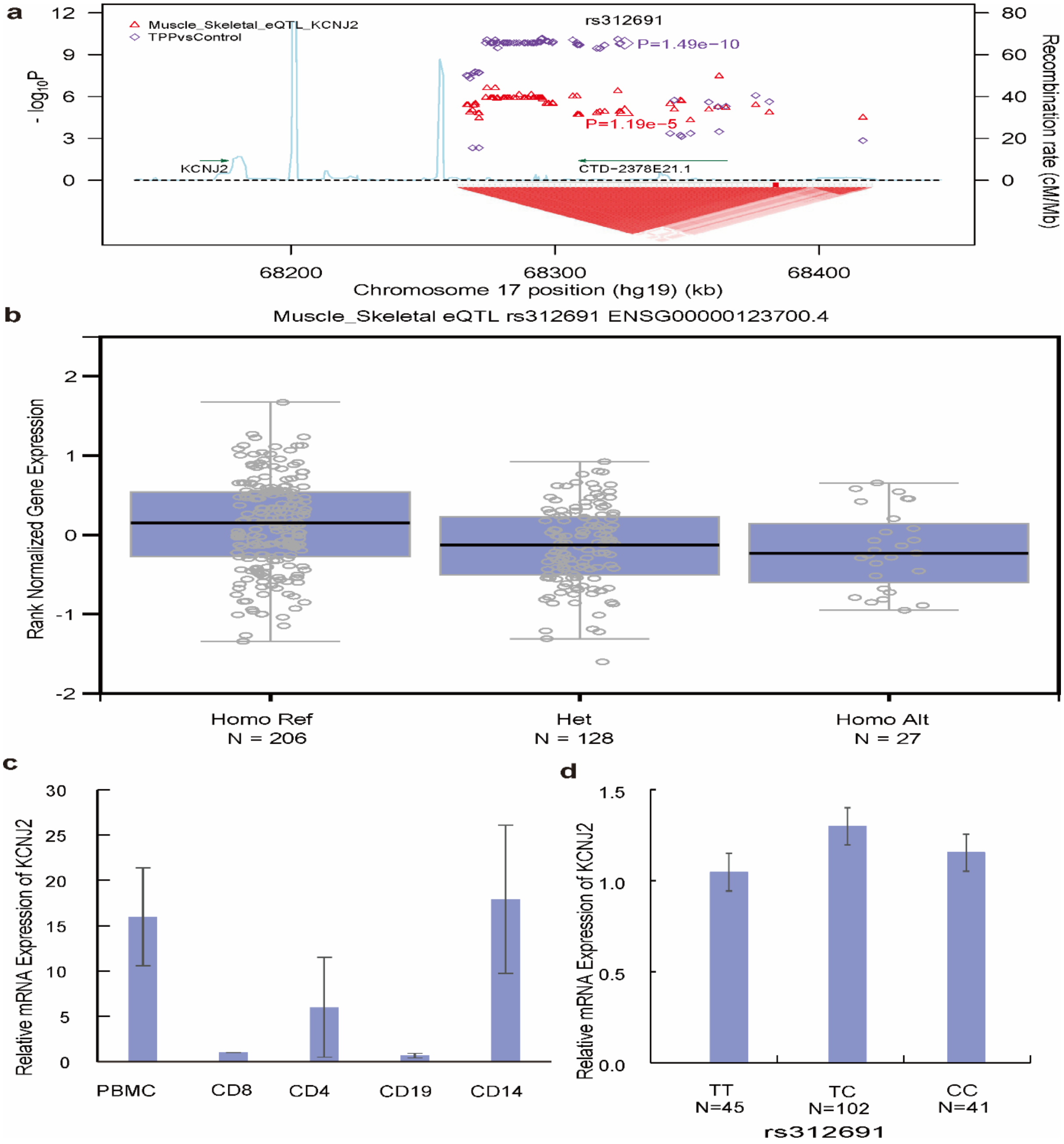

© 2019 Zhao S-X et al. *JAMA Network Open.*
